# Supplementary material for: Multimodal dynamic and unclonable anti-counterfeiting using robust diamond microparticles on heterogeneous substrate
Source: Nat Commun. 2023 May 2;14:2507. doi: 10.1038/s41467-023-38178-1 (PMC10154296; doi:10.1038/s41467-023-38178-1)
Supplement: Supplementary file 1 — Supplementary Information [file 41467_2023_38178_MOESM1_ESM.pdf]

Supplementary Information

Multimodal dynamic and unclonable anti-counterfeiting using robust diamond microparticles on heterogeneous substrate

Tongtong Zhang,[1] Lingzhi Wang,[1] Jing Wang,[2] Zhongqiang Wang,[3] Madhav Gupta,[1] Xuyun Guo,[4] Ye Zhu,[4] Yau Chuen Yiu,[1, 5] Tony K.C. Hui,[5] Yan Zhou,[6] Can Li,[1] Dangyuan Lei,[7] Kwai Hei Li,[8] Xinqiang Wang[3, 9], Qi Wang,[3, *] Lei Shao,[2, *] and Zhiqin Chu[1, 10, *]

[1] Department of Electrical and Electronic Engineering, The University of Hong Kong, Pokfulam Road, Hong Kong, China.

[2] State Key Laboratory of Optoelectronic Materials and Technologies, Guangdong Province Key Laboratory of Display Material and Technology, School of Electronics and Information Technology, Sun Yat-sen University, Guangzhou, China

[3] Dongguan Institute of Opto-Electronics, Peking University, Dongguan, China

[4] Department of Applied Physics, Research Institute for Smart Energy, The Hong Kong Polytechnic University, Hung Hom, Hong Kong, China

[5] Primemax Biotech Limited, Hong Kong, China

[6] School of Science and Engineering, The Chinese University of Hong Kong, Shenzhen, China

[7] Department of Material Science and Engineering, City University of Hong Kong, Hong Kong, China

[8] School of Microelectronics, Southern University of Science and Technology, Shenzhen, China

[9] State Key Laboratory for Mesoscopic Physics and Frontiers Science Center for Nano-optoelectronics, School of Physics, Peking University, Beijing, China

[10] School of Biomedical Sciences, The University of Hong Kong, Hong Kong, China

[*] Correspondence and requests for materials should be addressed to Q.W. (wangq@pku-ioe.cn), L.S. (shaolei5@mail.sysu.edu.cn), and Z.Q.C. (zqchu@eee.hku.hk).

Supplementary Table 1. The performance of the most representative optical PUFs in respect of capacity, diversity, safety, manufacturability, robustness and compatibility.

PUF system	Capacity	Diversity	Safety	Manufacturability	Robustness	Compatibility	Ref
This work	$C = R^m$<br>*(R: number of responses per pixel; m: number of pixels)*<br>$R = S + (n + 1) \times P$<br>*(S: Scattering responses; P: PL responses; n: oxidation time)*	1. Physical patterns<br>2. Scattering spectrum<br>3. PL intensity<br>4. Dynamic change of PL intensity	Non-toxic, excellent biosafety and eco-friendly[1, 2]	One-step commercial integrated CVD system	Ultrahigh stability in different extreme application scenarios, including harsh chemical environments, high temperature, mechanical abrasion, and UV light irradiation.	Compatible with microelectronics[3]; The method has the flexibility for the choice of substrate, growth rate, and in situ doping options[4].	-
Plasmonic NPs<br>*(e.g., gold NRs)*	$C = R^m$	1. Physical patterns<br>2. Scattering color<br>3. Polarization-dependent response	NA<br>*(Toxicity of gold NRs is dependent on their size, aspect ratio, and surface characteristics)*[5]	1. Chemical synthesis process<br>2. Drop-casting or dip-coating	NA<br>*(Good stability against oxidation; Good photostability; Environmental sensitive)*[6]	The NPs can be drop-cast onto a variety of substrates. (The physical properties of a substrate may influence the compatibility of the substrate with the NPs)<br>*(Gold is not CMOS-compatible)*[6]	7
Plasmonic NPs<br>*(e.g., gold NPs embedded with Raman probe molecules)*	$C = R^m$	1. Physical patterns<br>2. Raman intensity<br>3. Type of NPs	NA<br>*(Toxicity of gold NPs is dependent on their size, shape, and surface characteristics)*[8]	1. Chemical synthesis process<br>2. Drop-casting	NA<br>*(Good photostability and long-term stability)*[9]	The label can be fabricated on Scotch tape and transferred onto the surface of various products afterwards;<br>Small and large aggregations formed may affect its compatibility.<br>*(Solvent based strategy, may not be compatible with microelectronics)*[10]	11
Polymer dots<br>*(e.g., π-conjugated polymer semiconductors)*	$C = R^m$	1. Physical patterns<br>2. PL (color, lifetime)<br>3. Raman/Infrared spectrum	NA<br>*(High biosafety, and low toxicity, eco-friendly)*[10]	1. Chemical synthesis process<br>2. Spin-coating or drop-casting	Good photostability;<br>Stable in humidity/water and mechanical abrasion.<br>*(High stability toward environmental degradation)*[10]	Good applicability to substrates of varying degrees of surface energy and flexibility.<br>*(Many may have low water solubility; Solvent based strategy, may not be compatible with microelectronics)*[10]	12

Quantum dots *(II–VI semiconducting quantum dots, e.g., CdSe/CdS/ CdZnS)*	C = R^m	1. Physical patterns 2. PL (multicolor inks)	NA *(The material usually contains toxic ions, e.g., Cd, and not eco-friendly)*[13]	1. Chemical synthesis process 2. Inkjet printing	NA *(Photo-blinking)*[13] *(Surface modification may result in excellent acid and base resistance, photostability, and thermal stability)*[10]	NA *(Adaptable to various substrates; Solvent based strategy, may not be compatible with microelectronics)*[10]	14
Photonic crystals *(e.g., PSMA nanospheres)*	C = R^m	Structural colors *(Conflict between the "regular arrangement" of photonic crystals and the "randomness" of PUF)*	NA	1. Chemical synthesis process 2. Patterning process (e.g., self-assembly, and electrostatic interaction)	Nonfading, good durability	NA *(Complex fabrication process may affect its compatibility. Each anti-counterfeiting mark requires special design and complex fabrication process to allow a high-security level)*[13]	15
Perovskite NPs *(e.g., $CsPbBr_3$ NPs)*	C = R^m	1. Physical patterns 2. PL (multicolor inks, lifetime)	NA *(The material usually contains toxic ions, e.g., Pb, and not eco-friendly)*	1. Chemical synthesis process 2.Electrohydrody-namic printing	NA *(Poor photostability, poor thermal stability, and poor humidity stability)*[10]	NA *(Adaptable to various substrates; Solvent based strategy, may not be compatible with microelectronics)*[10]	16
Upconversion NPs *(e.g., Mn^{2+} co-doped in $NaLnF_4$ Ln=lanthanide)*	C = R^m	1. Physical patterns 2. Emission profiles (multicolor, lifetime, different excitations)	NA *(Some lanthanide dopant may be toxic, e.g., Tb, and not eco-friendly)*[13]	1. Chemical synthesis process 2. Stamping (used as inks)	Long-term stability at ambient conditions *(High chemical and physical stability toward environmental degradation because of the advantages of ceramics)*[10] *(Thermal quenching effect)*[13]	NA *(Large-scale controlled synthesis is lacked)*[13] *(Adaptable to various substrates; Solvent based strategy, may not be compatible with microelectronics)*[10]	17

Capacity: encoding capacity, the maximum number of unique PUFs that can be produced.
Diversity: the number of encoding methods that can be provided.
Safety: the degree of harm or danger the PUF may cause to the environment and human.
Manufacturability: the degree to which a PUF can be effectively manufactured given its design, cost, and distribution requirements.
Robustness: the ability to tolerate perturbations that might affect the PUF function.
Compatibility: the ability to work together in harmony because of well-matched characteristics.
NRs: nanorods.
NPs: nanoparticles.
NA: not available.
PSMA: polystyrene-maleic acid copolymer.

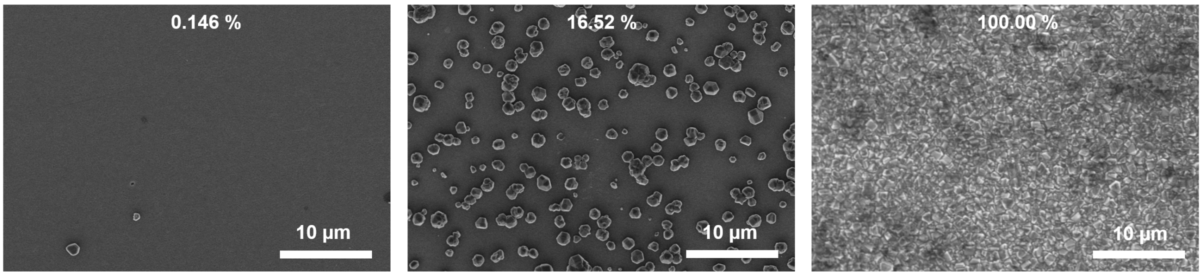

Supplementary Fig. 1. SEM image of the CVD-grown diamond particles on substrate with different coverage (calculated in the area of 30 µm × 40 µm).

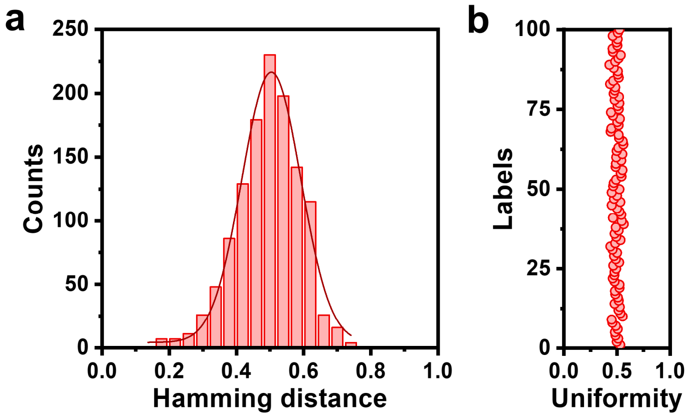

Supplementary Fig. 2. a The Hamming inter-distance (normalized) of the corresponding binary encoding matrix shown in Fig. 3c. **b** Uniformity of the binary keys generated from 100 labels.

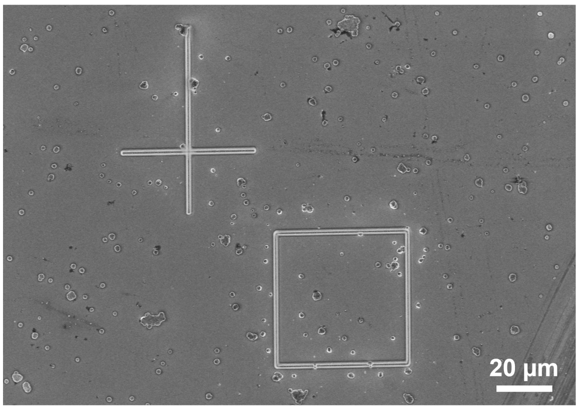

Supplementary Fig. 3. SEM image of the square and cross marker fabricated by focused-ion-beam (FIB) etching for the ease of locating the same area when authentication.

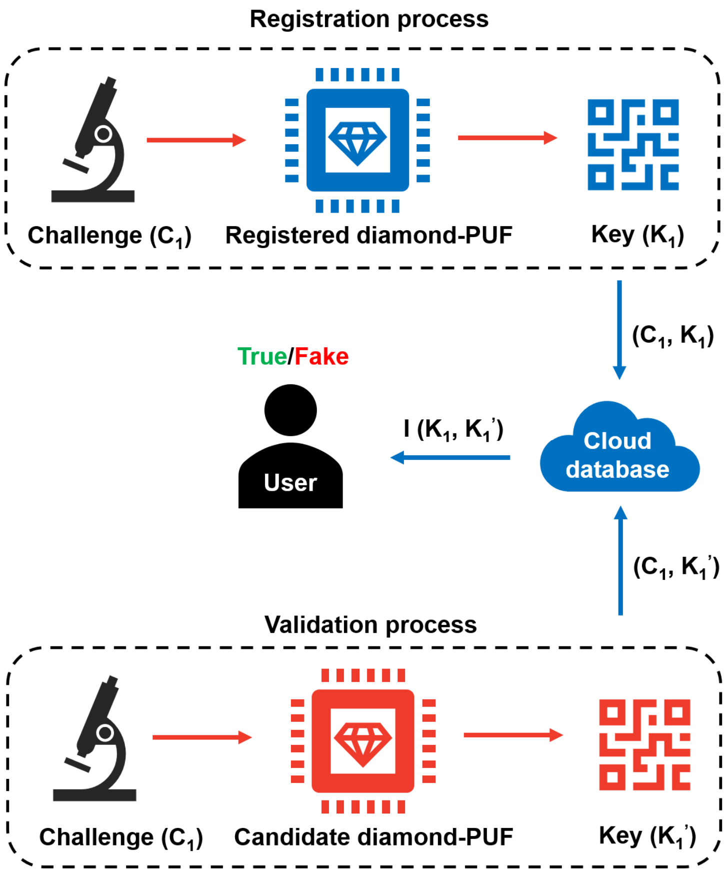

Supplementary Fig. 4. Schematic of authentication protocol based on diamond-PUFs, showing registration and authentication processes.

Supplementary Note 1:

As shown in Supplementary Fig. 4. The execution of authentication can be divided into two steps, including the registration and validation processes.

(1) In the registration stage, input challenges (C_1) are projected onto the diamond-PUF to generate the associated response (R_1), which is further transformed into the key (K_1). Then, the challenge-response pairs composed of the registered information of C_1 as well as K_1 are stored in the cloud database.

(2) In the validation stage, the authenticator randomly selects C_1 from the cloud database to challenge the candidate PUF, and then obtains a check code K_1'. Hereafter, the check code is uploaded to the data cloud to compare the similarity index (I) or Hamming distance between K_1 and K_1'. If I (K_1, K_1') is greater than a preset threshold, the verification is true. Otherwise, the verification result is false.

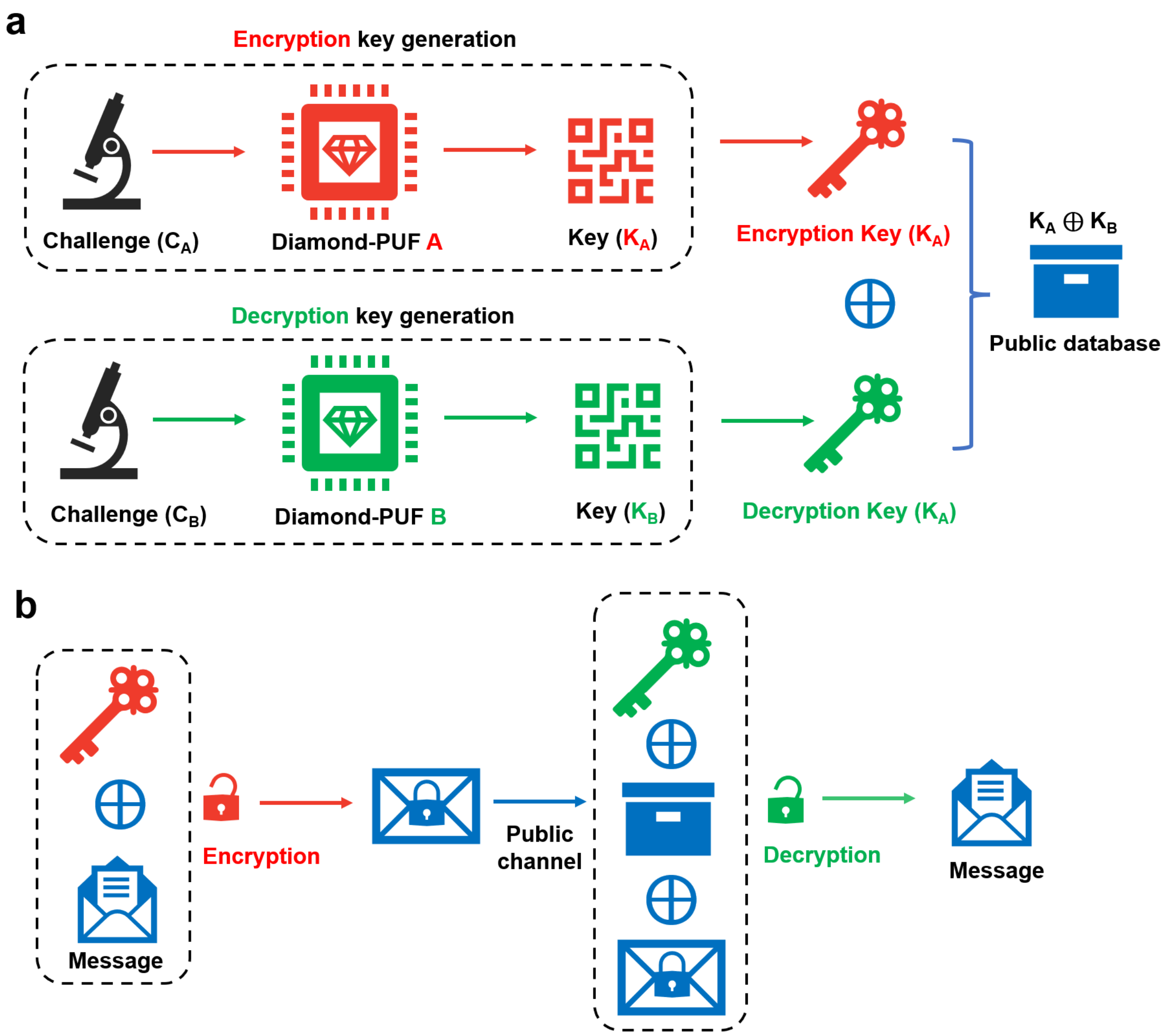

Supplementary Fig. 5. Schematic of a possible information encryption and decryption scheme using the diamond-PUFs. **a** The generation of encryption and decryption keys. **b** The encryption and decryption processes.

Supplementary Note 2:

Secure communication between two parties is important for everyday information security. Here, our diamond-PUF label has the application potential in secure information encryption[18]. As shown in Supplementary Fig. 5, a possible information encryption and decryption scheme using our diamond-PUF label is proposed. Firstly, two unique diamond-PUFs (A and B) are used to generate random and secret keys (K_A and K_B), which belong to the encryption and decryption side, respectively. The two keys are then mixed and stored in a public database ($K_A \oplus K_B$), shown in Supplementary Fig. 5a. During the information encryption stage, the diamond-PUF key K_A is used to encrypt the message (M) to generate the ciphertext ($M \oplus K_A$), which is then transmitted to the decryption side via a public channel. As for the information decryption, diamond-PUF key K_B is then used to decrypt the ciphertext by mixing the public database with the key K_B, i.e., $K_B \oplus (K_A \oplus K_B) \oplus (M \oplus K_A) = M$. Due to the unique and irreproducible nature of the diamond-PUF labels, there is no need for secret key sharing and storage during the encryption and decryption processes, ensuring efficient and secure information communication.

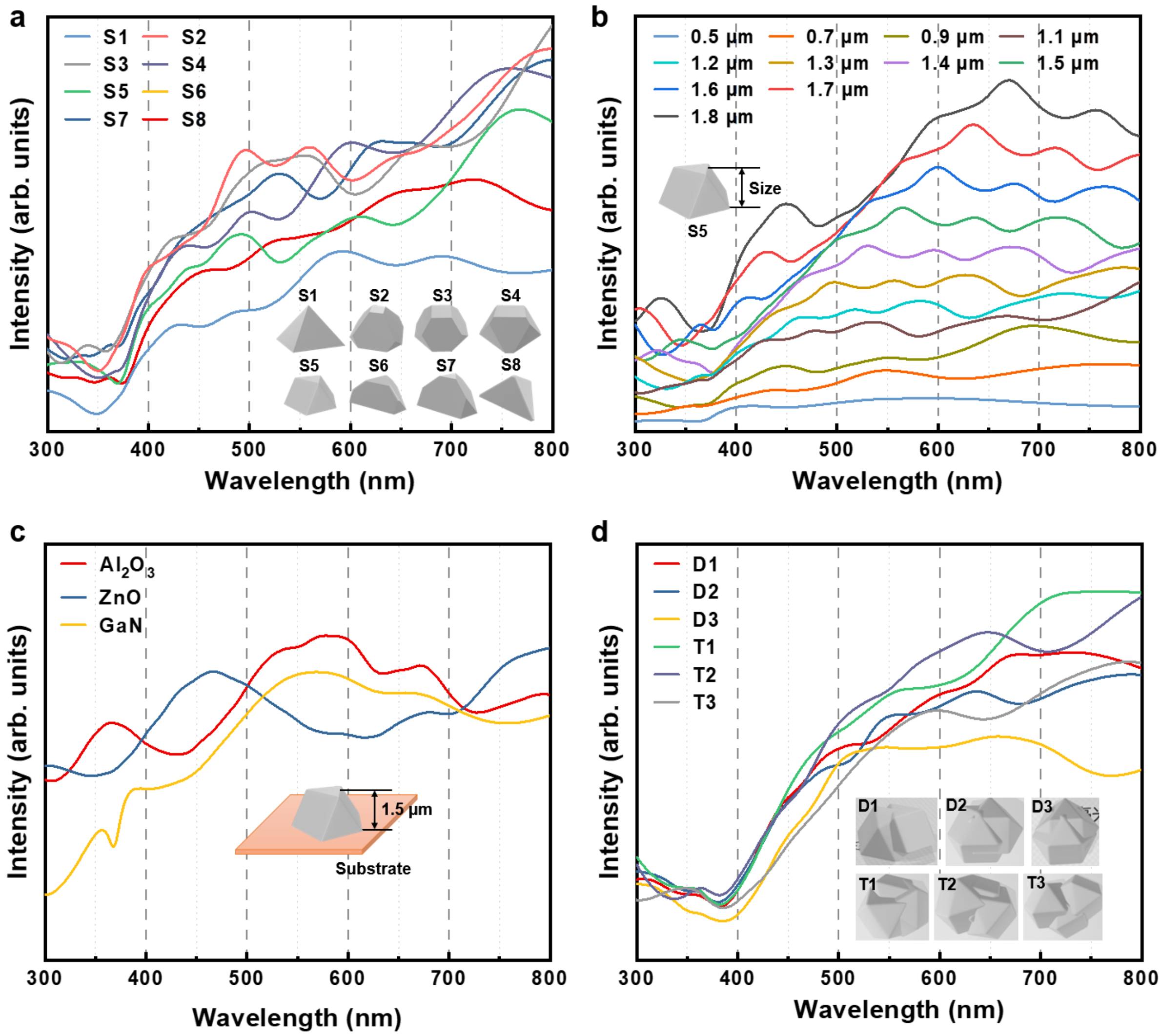

Supplementary Fig. 6. Numerical calculated scattering spectra of diamond microparticles with different **a** shapes (on Si substrate), **b** sizes (on Si substrate), **c** substrates, and **d** crystallinity (D: double crystals, T: triple crystals).

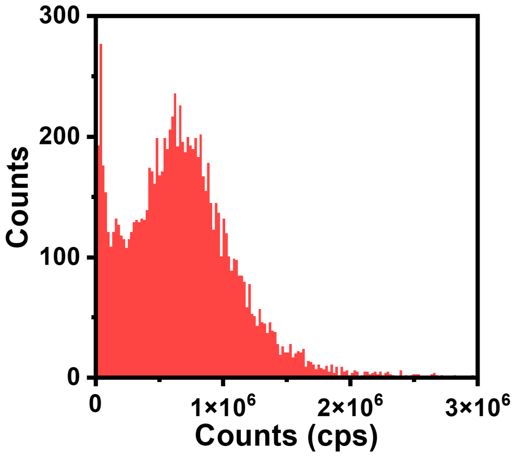

Supplementary Fig. 7. The distribution of the PL intensity (photon counts: cps) in the measured area shown in Fig. 4b.

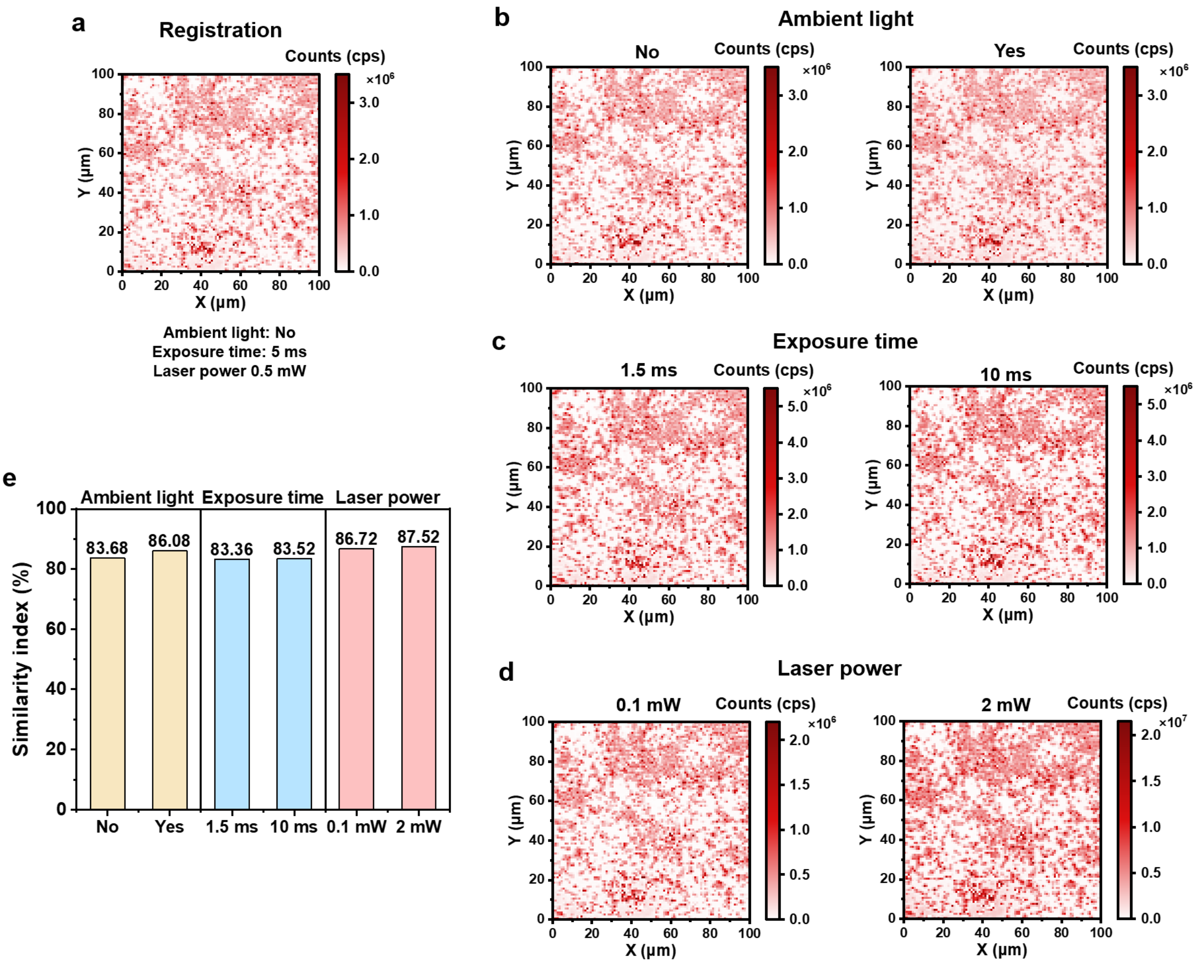

Supplementary Fig. 8. The PL images of the same diamond-PUF label in **a** standard registration condition and different measurement conditions (**b** with and without ambient light, **c** different exposure time, and **d** different laser power) for validation. **e** The similarity index (%) of the quaternary keys generated by the same diamond-PUF label at different measurement conditions. (Note: the condition of the registration process: no ambient light, 5 ms exposure time, 0.5 mW laser power. For the validation process, the conditions other than those tested remain unchanged, e.g., the exposure time and laser power remain unchanged (5 ms, 0.5 mW) when testing the effect of ambient light.)

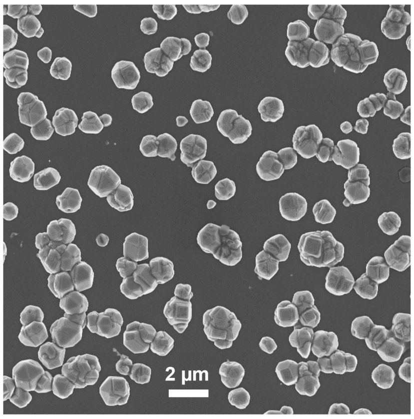

Supplementary Fig. 9. SEM image of the CVD-grown diamond particles after the 4 rounds of air oxidation in the selected area in Fig. 5a for time-dependent encoding.

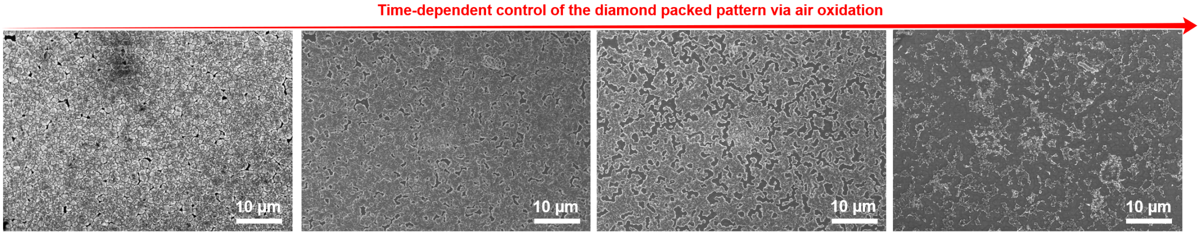

Supplementary Fig. 10. SEM images of the multigenerational microstructures of the diamond microparticles after air oxidation.

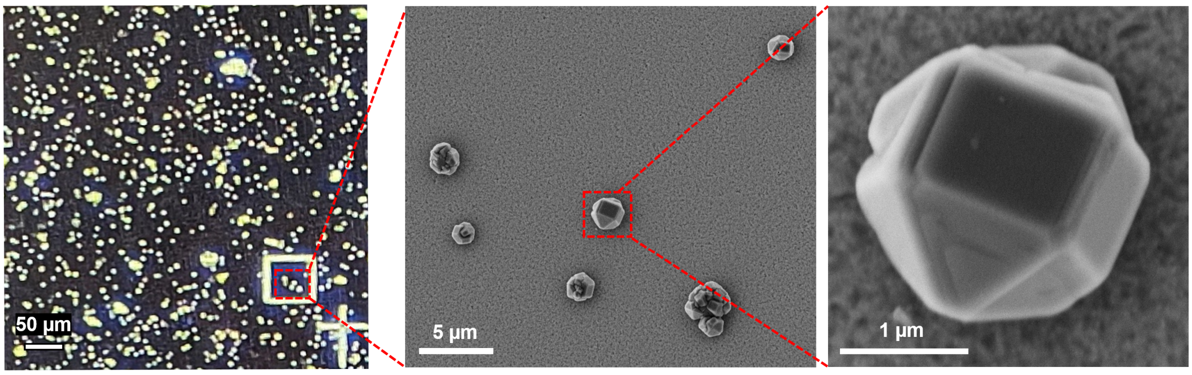

Supplementary Fig. 11. SEM images of the marked CVD-grown diamond particles after the stability treatments shown in Fig. 6a

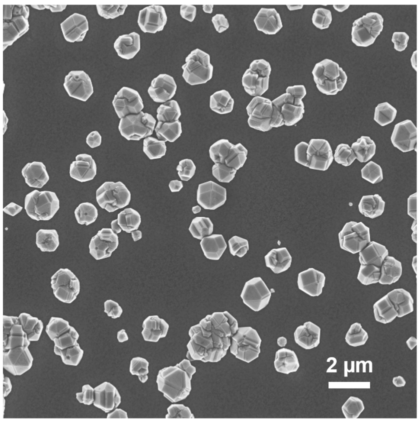

Supplementary Fig. 12. SEM image of the CVD-grown diamond particles after the stability treatments in the measured area of confocal fluorescence image in Fig. 6d

Supplementary Table 2. The mildest estimation of detailed cost for fabricating one piece 2-inch sized diamond-PUF.

Item	Unit price	Consumption	Cost (USD)	Remarks
Electricity	0.11 USD/kWh	3.6 kWh	0.396	The average power of our MPCVD equipment during the fabrication process is 2.4 kW with a running time of 1.5 hours, and the local electricity price is 0.11 USD/kWh.
Gas (Hydrogen)	0.0017 USD/L	8.46 L	0.014	The average price of bottled hydrogen is 8.86 USD/bottle (13.5 MPa, 40 L), equal to 0.0017 USD/L (atm). During diamond growth, the hydrogen flow rate is ~0.094 L/min with a running time of 90 minutes.
Gas (Methane)	0.033 USD/L	0.48 L	0.016	The average price of bottled methane is 177 USD/bottle (13.5 Mpa, 40 L), equal to 0.033 USD/L (atm). During diamond growth, the methane flow rate is ~0.006 L/min with a running time of 80 minutes.
Substrate	0.144 USD/piece	1 piece	0.144	The solar cell grade monocrystalline silicon wafer with a diameter of 2 inches and a thickness of 0.18 mm is used as the substrate for diamond growth in this study, and it can be purchased at a price of ~0.144 USD/piece from the market.
Diamond seeds	1.31 USD/g	0.0005 g	0.001	Diamond nanoparticles with a particle size of ~50 nm can be purchased at ~1.31 USD/g from the market. After the salt-assisted air-oxidation treatment, the diamond seeds are dispersed into deionized water with a concentration of ~0.1 wt%, and an average of 0.5 g of the seeds solution is spin-coated onto a 2-inch silicon wafer.
Equipment (MPCVD system)	0.846 USD/hour	1.5 h	1.269	We used the current market price ~100000 USD for calculation. The MPCVD system will depreciate over 15 years with a utilization rate ~90%. Then, the equipment purchase (one-time investment) can be converted into the running/maintaining cost ~0.846 USD/hour.
House rent	0.091 USD/hour	1.5 h	0.127	The local house rent for placing the MPCVD system is ~720 USD/year. Considering the utilization rate (~90%) of the MPCVD system, the unit cost of house rent can be converted into ~0.091 USD/hour.
Labor	0.3 USD/hour	1.5 h	0.450	The local labor cost is ~3 USD/hour. Considering the MPCVD system is highly integrated and does not require complex operations, one person can operate 10 or more equipment at the same time, so the unit labor cost can be converted into ~0.3 USD/hour. In the future, the labor cost will be lower or reduced to zero, because the final production line will develop into full-machine automatic production.
Total			**2.417**	This cost could be further reduced along with the significant technological advancement over the years.

Supplementary Note 3:

The above calculated total cost is the unit price for fabricating one piece of 2-inch sized diamond-PUF label. Due to the high encoding capacity of the diamond-PUFs, a tiny working area can meet the purpose of anti-counterfeiting. In our study, the largest area used for encoding is 200 μm × 200 μm (Fig. 3a). Thus, a 2-inch wafer can be cut into a maximum of ~4.9×10^4 pieces of working labels, and the cost of an individual working label will be significantly low (~0.0001 USD). And it will be further reduced if a larger-sized substrate is used for the diamond-PUF fabrication. In addition, we might need to consider the extra cost for processing (i.e., cutting the 2-inch wafer into many small pieces for our purpose) the Si wafer (~10 USD/piece), but this is negligible when considering the large number (on the order of ~10^4 pieces) of generated anti-counterfeiting labels from such a 2-inch wafer.

Supplementary Note 4:

Uniformity. The uniformity metric can be calculated using the following equation:

$$\text{Uniformity} = \frac{1}{n}\sum_{i=1}^{n} R_i \tag{1}$$

where, R_i is the ith bit response from the n-bit key.

Similarity index. The similarity index (I) is used to detect the degree of similarity between different PUFs, which can be calculated by the following equation:

$$\mathrm{I} = \frac{A}{B} \times 100\% \tag{2}$$

where, A is the same number of pixels between two PUFs, B is the total number of pixels in PUF.

Hamming distance. The Hamming distance between two keys is the minimum number of substitutions required to change one key into the other. The Hamming inter-distance is used to quantify the uniqueness of a PUF, which is the ability to distinguish a PUF from others. And the Hamming intra-distance is used to quantify the reliability of the same PUF label, checking if it has the ability to generate consistent keys under multiple measurements. The uniqueness and reliability are evaluated using the Hamming distance as below:

$$\text{Uniqueness} = \frac{2}{N(N-1)}\sum_{i=1}^{N-1}\sum_{j=i+1}^{N}\frac{HD(R_i,R_j)}{n} \tag{3}$$

where R_i and R_j are n-bit keys of the ith and jth PUF, and N is the total number of PUFs.

$$\text{Reliability} = \frac{1}{N}\sum_{i=1}^{N}\frac{HD(R_0,R_i)}{n} \tag{4}$$

where R_0 is the original n-bit key of the PUF, and R_i is the n-bit key generated by the same PUF from ith measurement among total N times of measurements.

Supplementary References

1. Mochalin, V. N., Shenderova, O., Ho, D. & Gogotsi, Y. The properties and applications of nanodiamonds. *Nat. Nanotechnol.* **7**, 11−23 (2011).
2. Hu, Y. W., et al. Flexible and biocompatible physical unclonable function anti-counterfeiting label. *Adv. Funct. Mater.* **31**, 2102108 (2021).
3. Auciello, O. & Aslam, D. M. Review on advances in microcrystalline, nanocrystalline and ultrananocrystalline diamond films-based micro/nano-electromechanical systems technologies. *J. Mater. Sci.* **56**, 7171−7230 (2021).
4. Arnault, J.-C., Saada, S. & Ralchenko, V. Chemical vapor deposition single-crystal diamond: a review. *Phys. Status Solidi RRL* **16**, 2100354 (2021).
5. Zarska, M., et al. Biological safety and tissue distribution of (16-mercaptohexadecyl)trimethylammonium bromide-modified cationic gold nanorods. *Biomaterials* **154**, 275−290 (2018).
6. Ibrar, M. & Skrabalak, S. E. Designer plasmonic nanostructures for unclonable anticounterfeit tags. *Small Struct.* **2**, 2100043 (2021).
7. Smith, J. D., et al. Plasmonic anticounterfeit tags with high encoding capacity rapidly authenticated with deep machine learning. *ACS nano* **15**, 2901−2910 (2021).
8. Alkilany, A. M. & Murphy, C. J. Toxicity and cellular uptake of gold nanoparticles: what we have learned so far? *J. Nanopart. Res.* **12**, 2313−2333 (2010).
9. Zhang, Y., Gu, Y., He, J., Thackray, B. D. & Ye, J. Ultrabright gap-enhanced Raman tags for high-speed bioimaging. *Nat. Commun.* **10**, 3905 (2019).
10. Abdollahi, A., Roghani-Mamaqani, H., Razavi, B. & Salami-Kalajahi, M. Photoluminescent and chromic nanomaterials for anticounterfeiting technologies: recent advances and future challenges. *ACS nano* **14**, 14417−14492 (2020).
11. Gu, Y., He, C., Zhang, Y., Lin, L., Thackray, B. D. & Ye, J. Gap-enhanced Raman tags for physically unclonable anticounterfeiting labels. *Nat. Commun.* **11**, 516 (2020).
12. Kayaci, N., Ozdemir, R., Kalay, M., Kiremitler, N. B., Usta, H. & Onses, M. S. Organic light-emitting physically unclonable functions. *Adv. Funct. Mater.* **32**, 2108675 (2021).
13. Ren, W., Lin, G., Clarke, C., Zhou, J. & Jin, D. Optical nanomaterials and enabling technologies for high-security-level anticounterfeiting. *Adv. Mater.* **32**, 1901430 (2020).
14. Liu, Y., et al. Inkjet-printed unclonable quantum dot fluorescent anti-counterfeiting labels with artificial intelligence authentication. *Nat. Commun.* **10**, 2409 (2019).
15. Wu, J., et al. Unclonable photonic crystal hydrogels with controllable encoding capacity for anticounterfeiting. *ACS Appl. Mater. Interfaces* **14**, 2369−2380 (2022).
16. Yakunin, S., et al. Radiative lifetime-encoded unicolour security tags using perovskite nanocrystals. *Nat. Commun.* **12**, 981 (2021).
17. Liu, X., et al. Binary temporal upconversion codes of Mn^{2+}-activated nanoparticles for multilevel anti-counterfeiting. *Nat. Commun.* **8**, 899 (2017).
18. Arppe, R. & Sorensen, T. J. Physical unclonable functions generated through chemical methods for anti-counterfeiting. *Nat. Rev. Chem.* **1**, 0031 (2017).
