# Supplementary figures and images for: Multimodal dynamic and unclonable anti-counterfeiting using robust diamond microparticles on heterogeneous substrate
Source: Nat Commun. 2023 May 2;14:2507. doi: 10.1038/s41467-023-38178-1 (PMC10154296; doi:10.1038/s41467-023-38178-1)

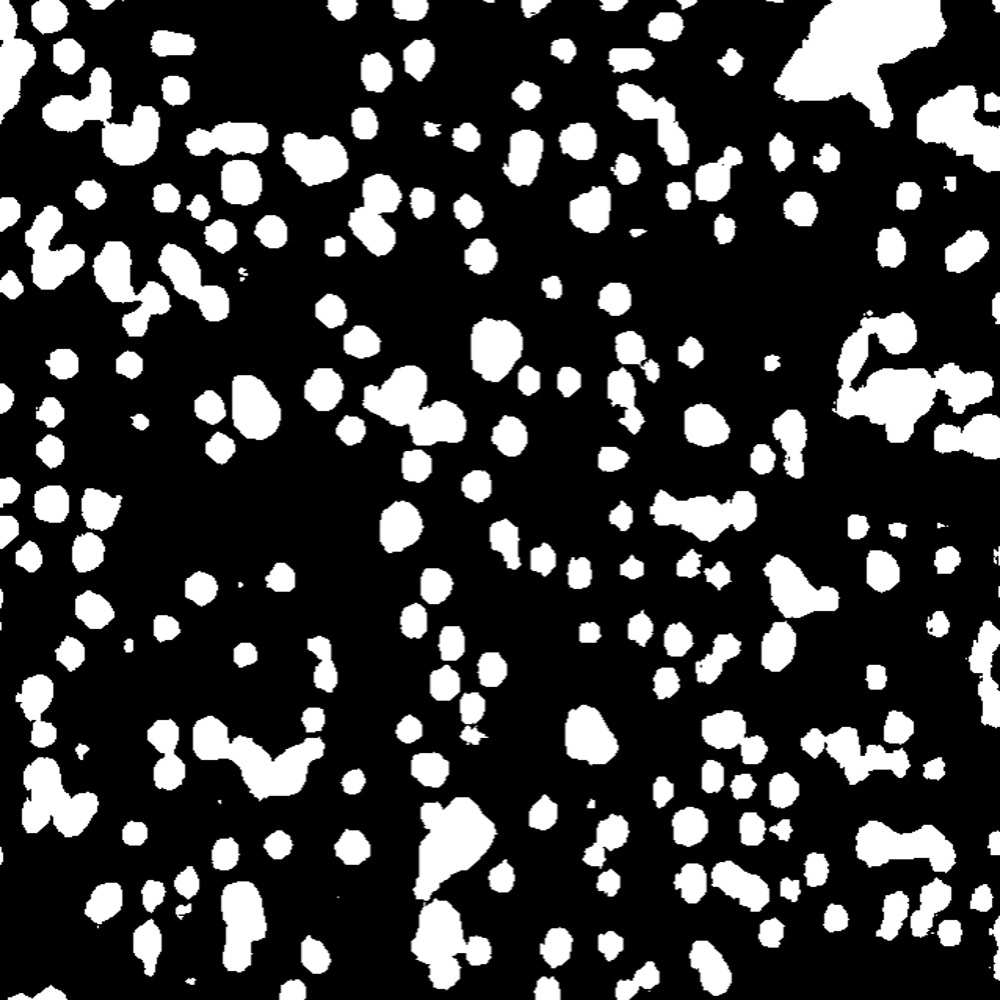

Supplement: Supplementary file 3 — Source Data [file 41467_2023_38178_MOESM3_ESM.zip › Source Data/Fig 3/1.jpg]

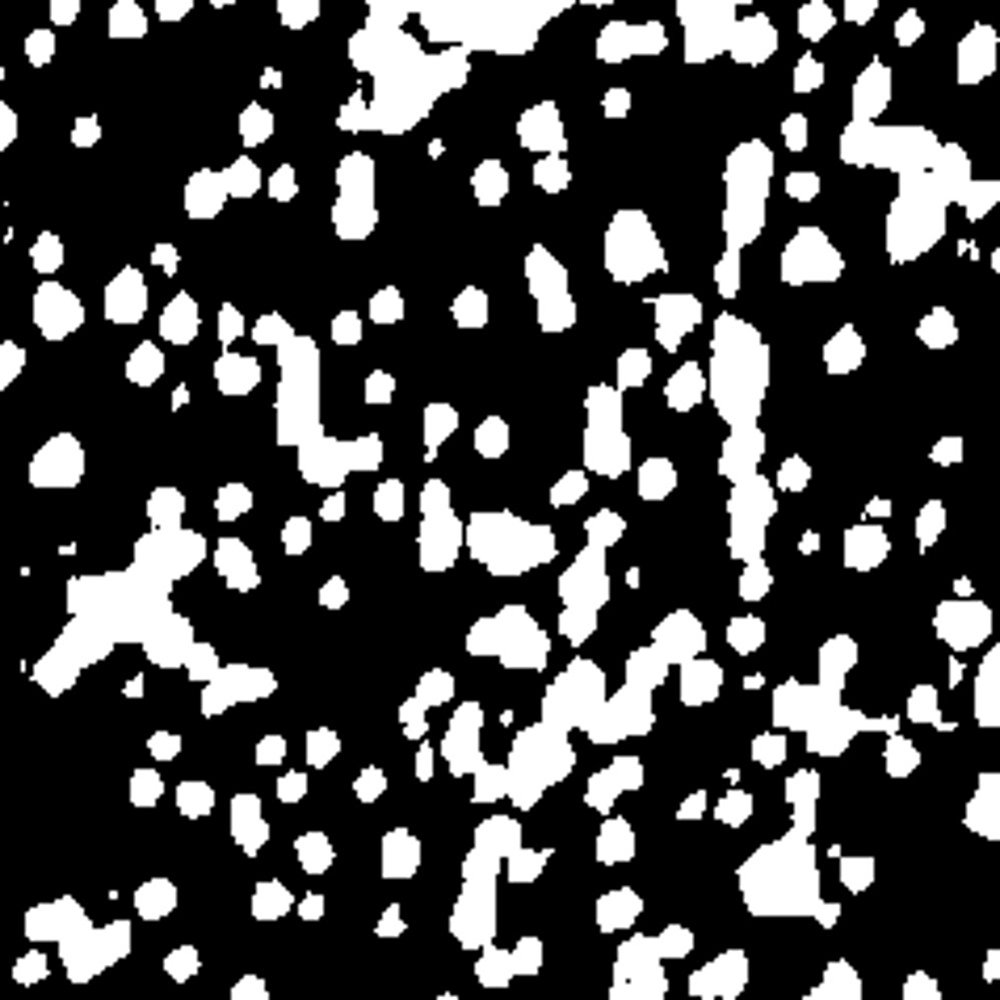

Supplement: Supplementary file 3 — Source Data [file 41467_2023_38178_MOESM3_ESM.zip › Source Data/Fig 3/10.jpg]

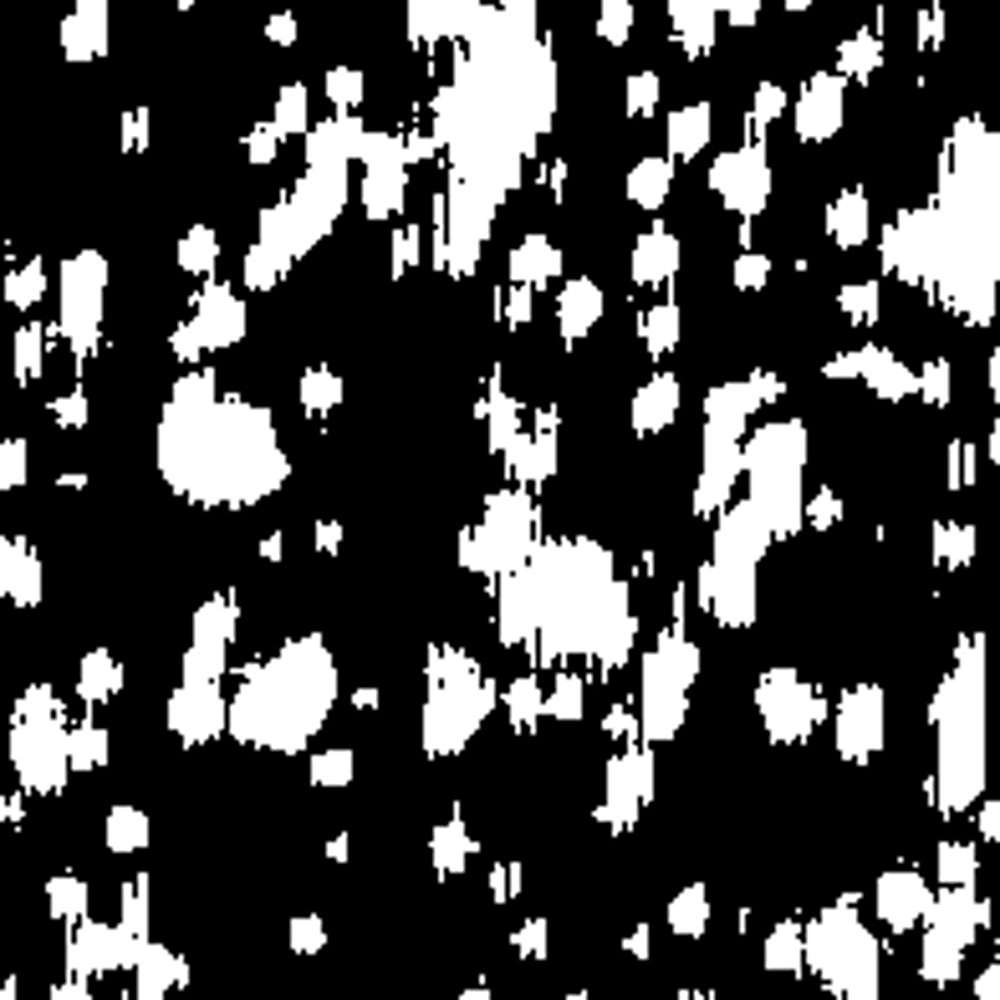

Supplement: Supplementary file 3 — Source Data [file 41467_2023_38178_MOESM3_ESM.zip › Source Data/Fig 3/100.jpg]

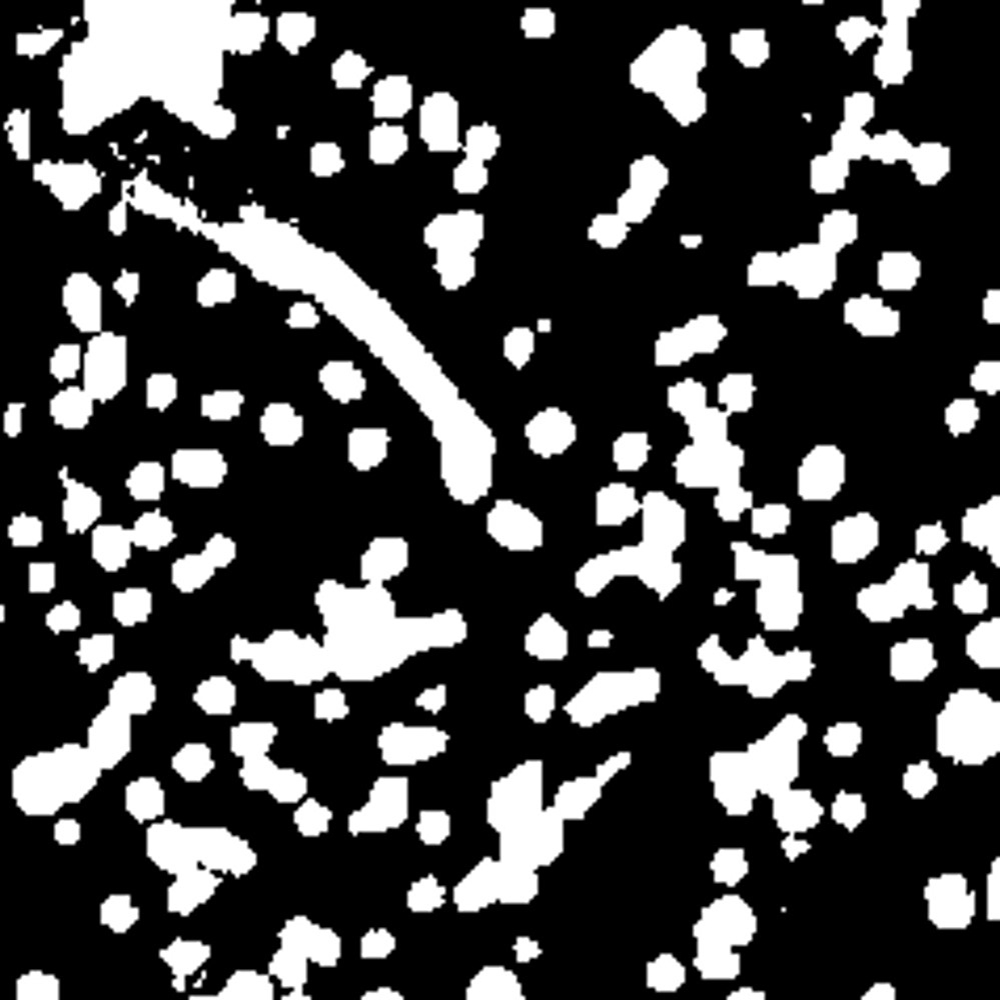

Supplement: Supplementary file 3 — Source Data [file 41467_2023_38178_MOESM3_ESM.zip › Source Data/Fig 3/11.jpg]

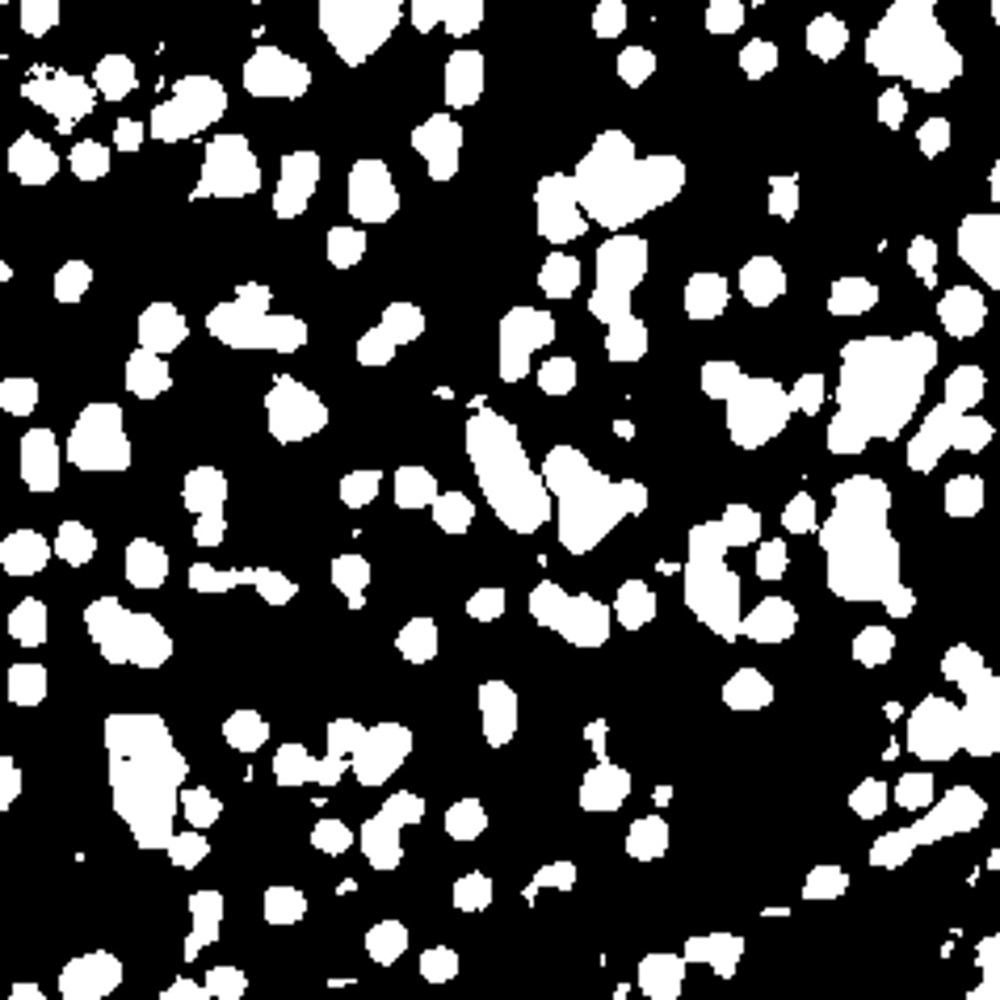

Supplement: Supplementary file 3 — Source Data [file 41467_2023_38178_MOESM3_ESM.zip › Source Data/Fig 3/12.jpg]

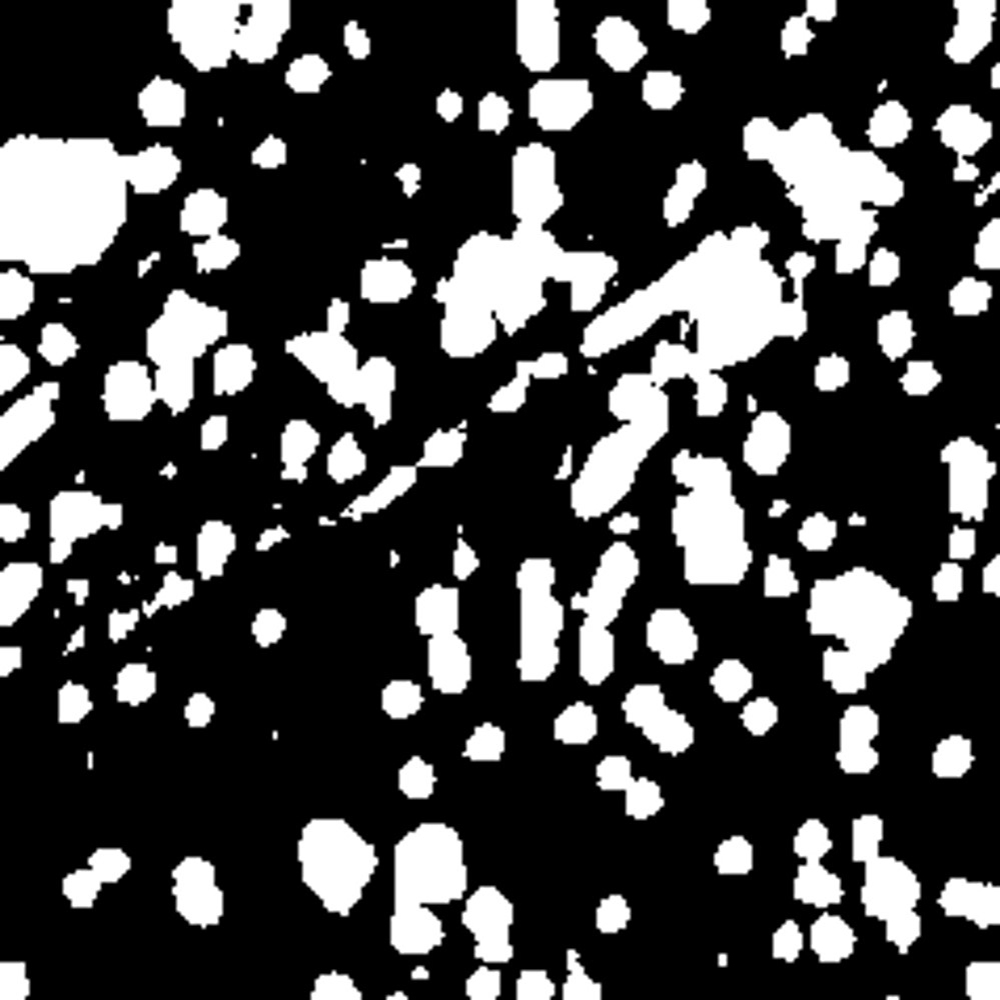

Supplement: Supplementary file 3 — Source Data [file 41467_2023_38178_MOESM3_ESM.zip › Source Data/Fig 3/13.jpg]

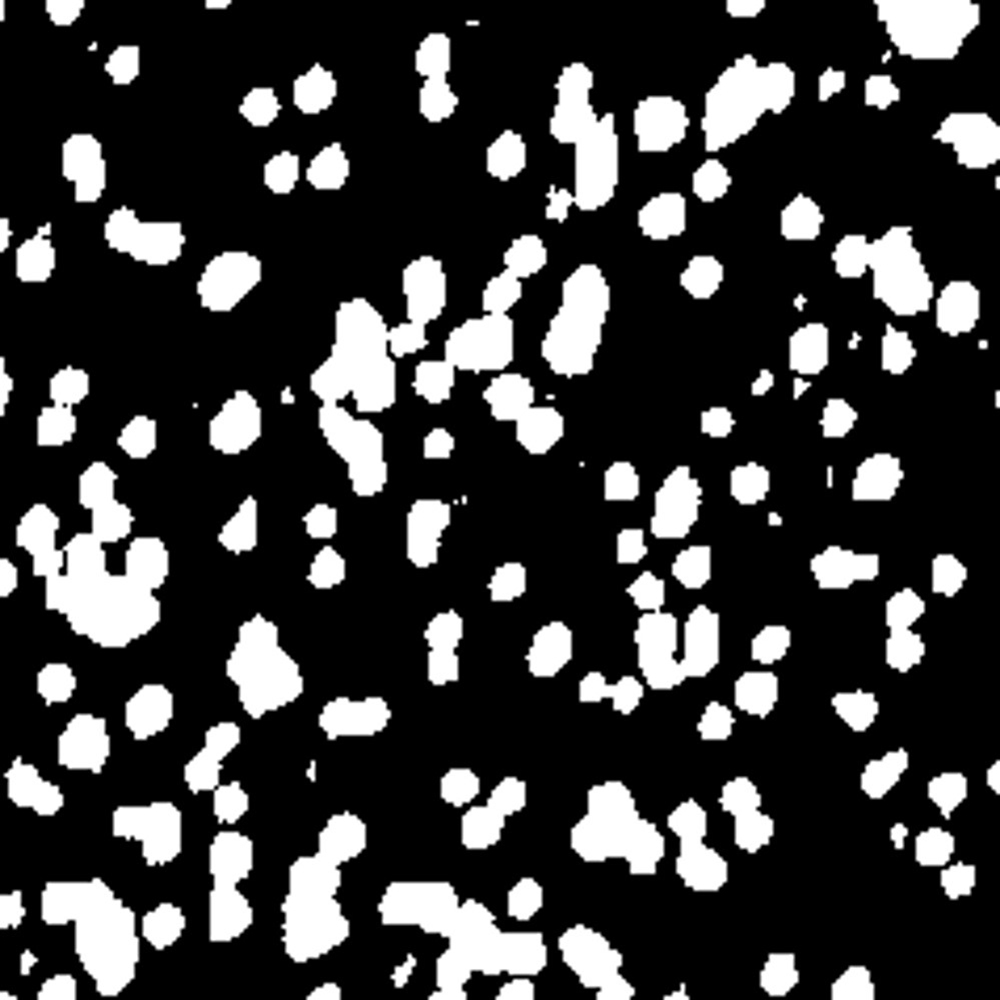

Supplement: Supplementary file 3 — Source Data [file 41467_2023_38178_MOESM3_ESM.zip › Source Data/Fig 3/14.jpg]

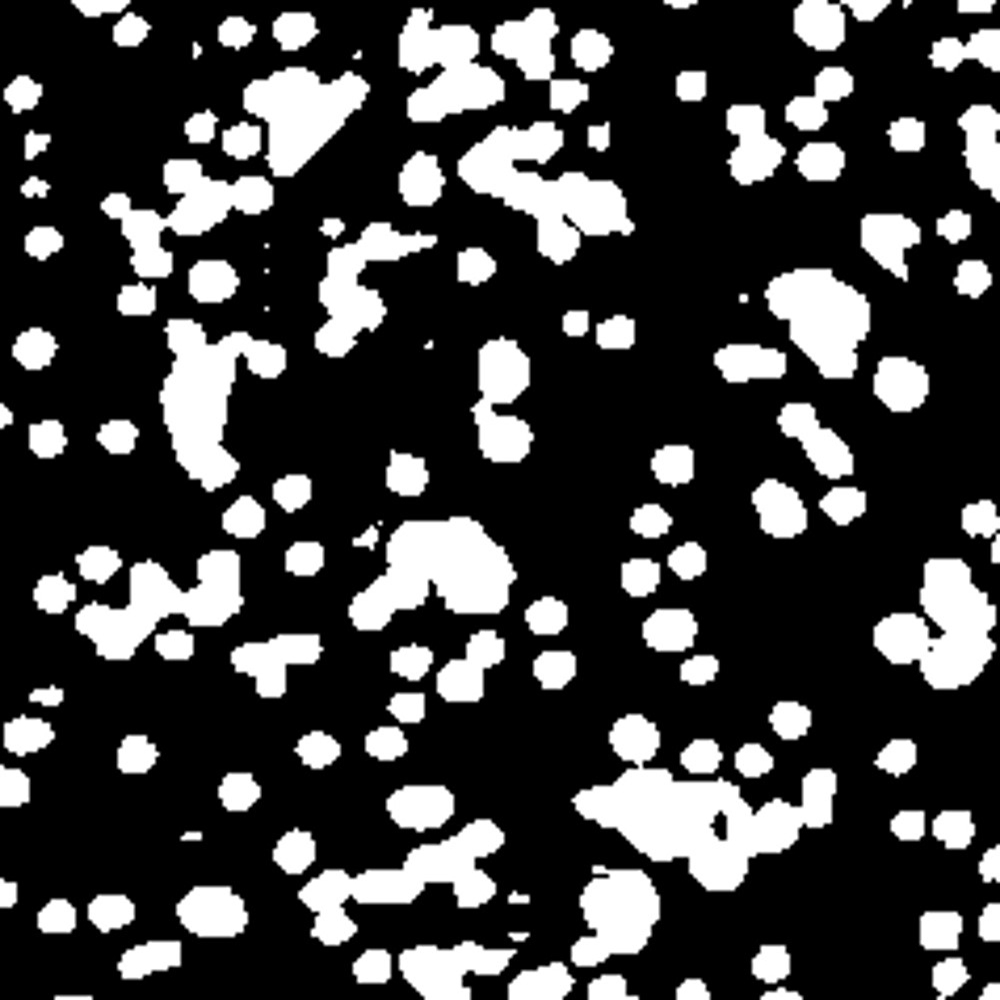

Supplement: Supplementary file 3 — Source Data [file 41467_2023_38178_MOESM3_ESM.zip › Source Data/Fig 3/15.jpg]

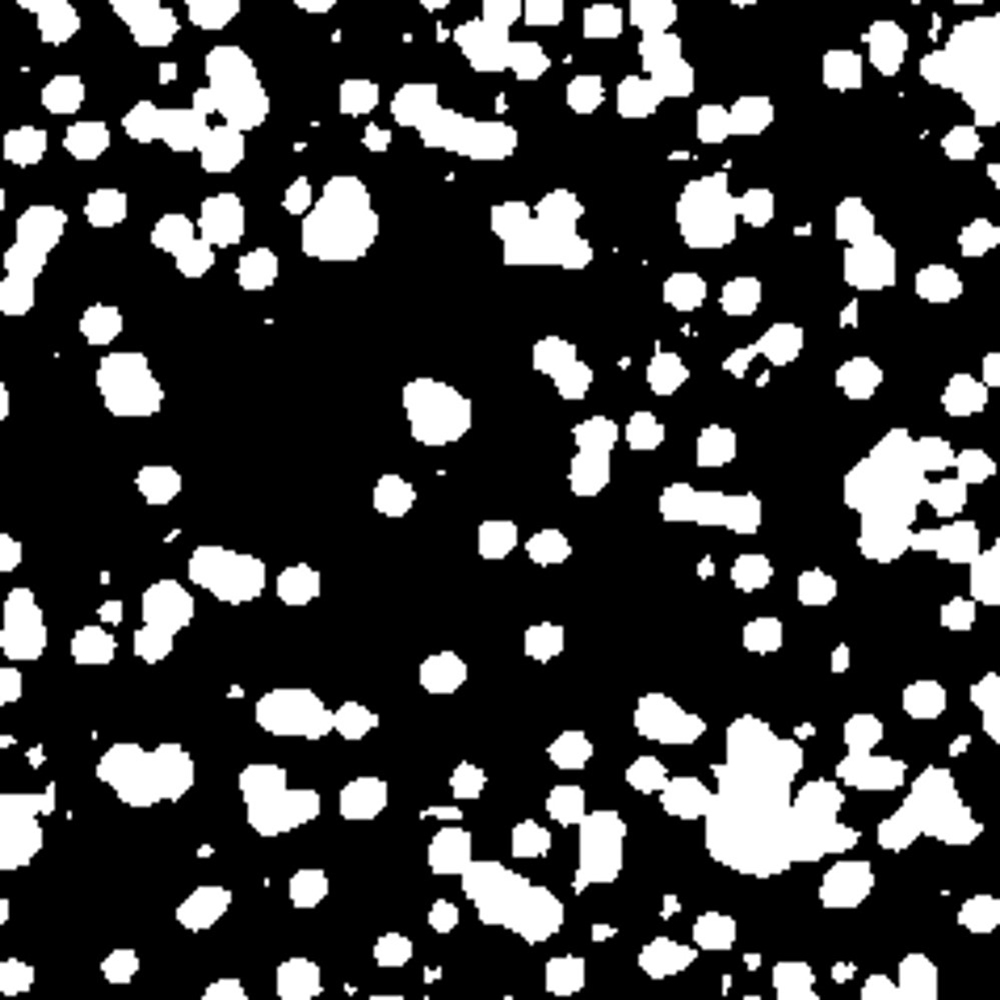

Supplement: Supplementary file 3 — Source Data [file 41467_2023_38178_MOESM3_ESM.zip › Source Data/Fig 3/16.jpg]

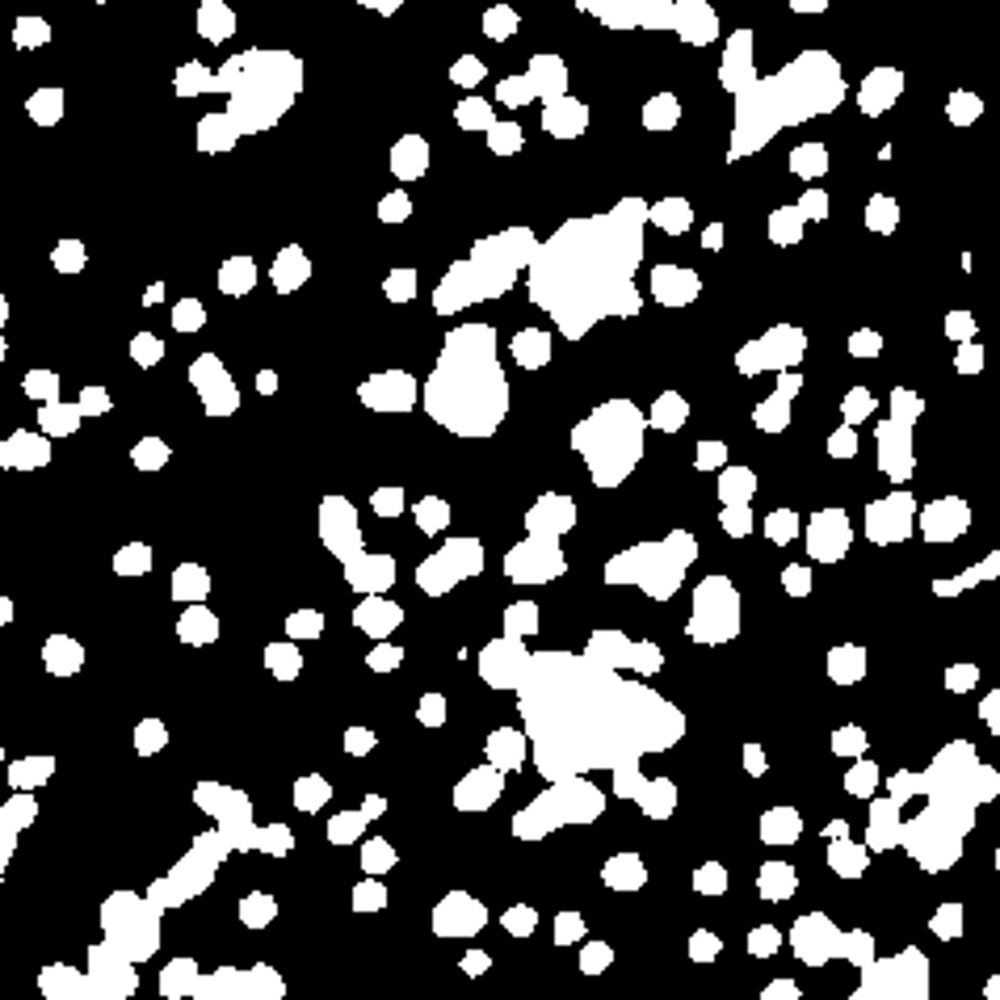

Supplement: Supplementary file 3 — Source Data [file 41467_2023_38178_MOESM3_ESM.zip › Source Data/Fig 3/17.jpg]

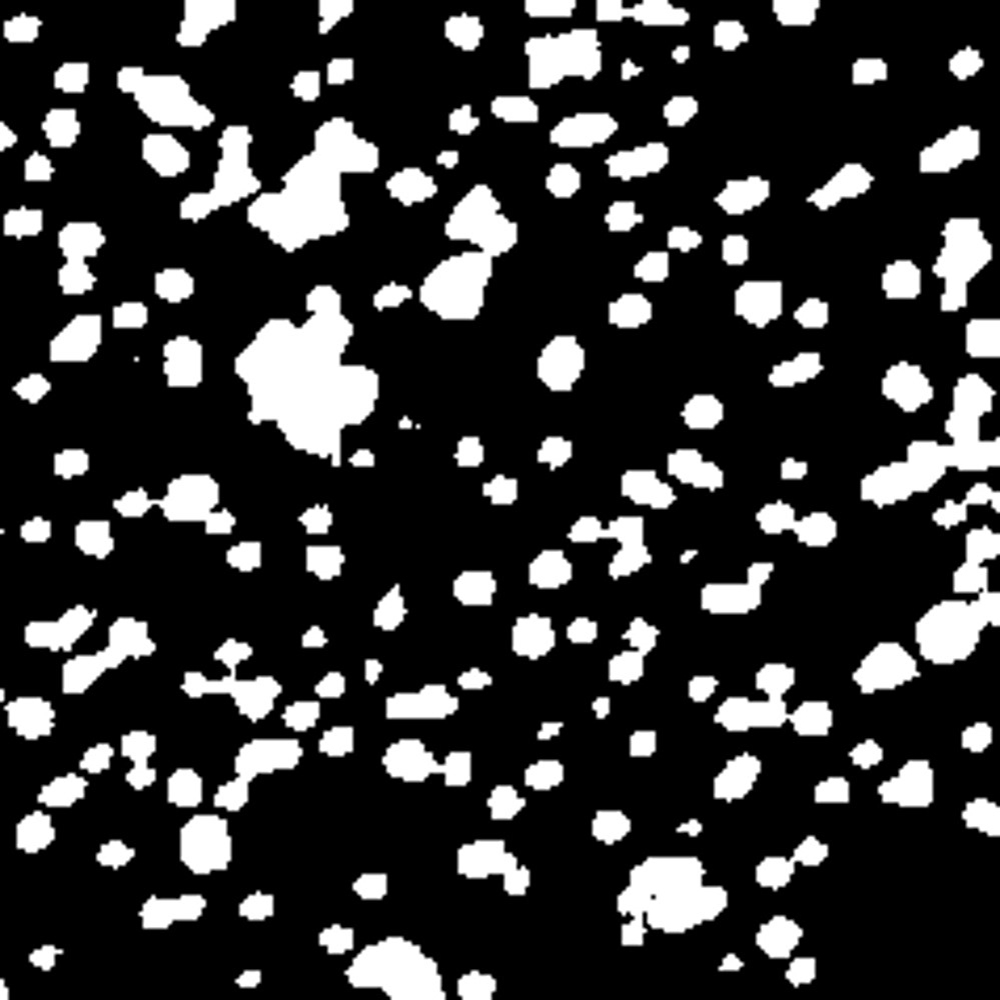

Supplement: Supplementary file 3 — Source Data [file 41467_2023_38178_MOESM3_ESM.zip › Source Data/Fig 3/18.jpg]

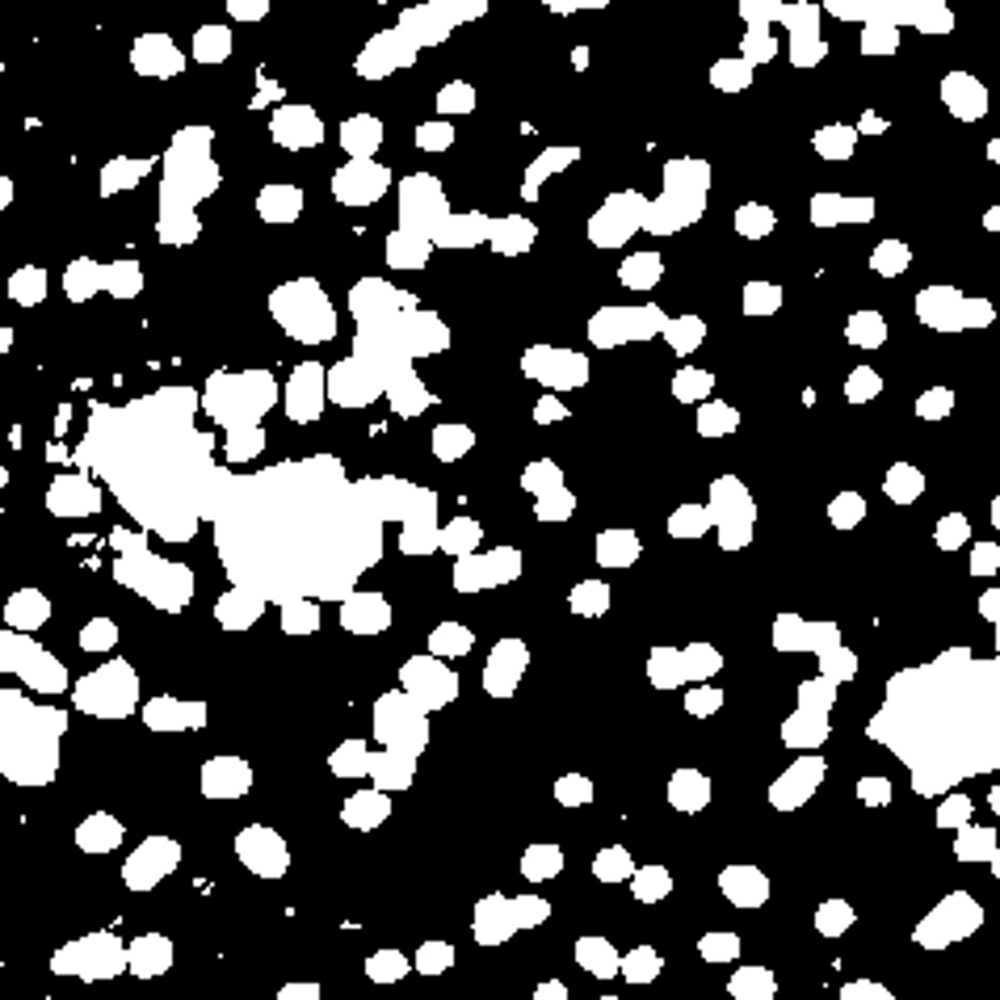

Supplement: Supplementary file 3 — Source Data [file 41467_2023_38178_MOESM3_ESM.zip › Source Data/Fig 3/19.jpg]

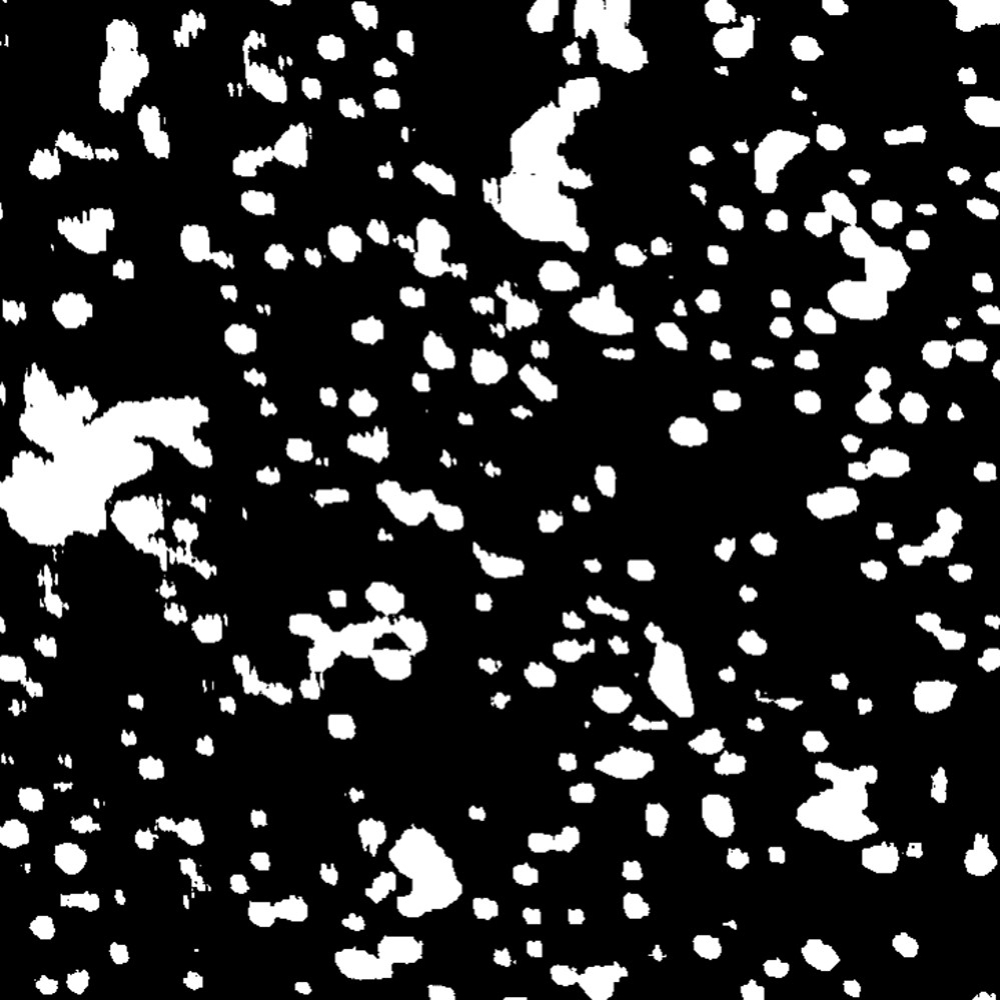

Supplement: Supplementary file 3 — Source Data [file 41467_2023_38178_MOESM3_ESM.zip › Source Data/Fig 3/2.jpg]

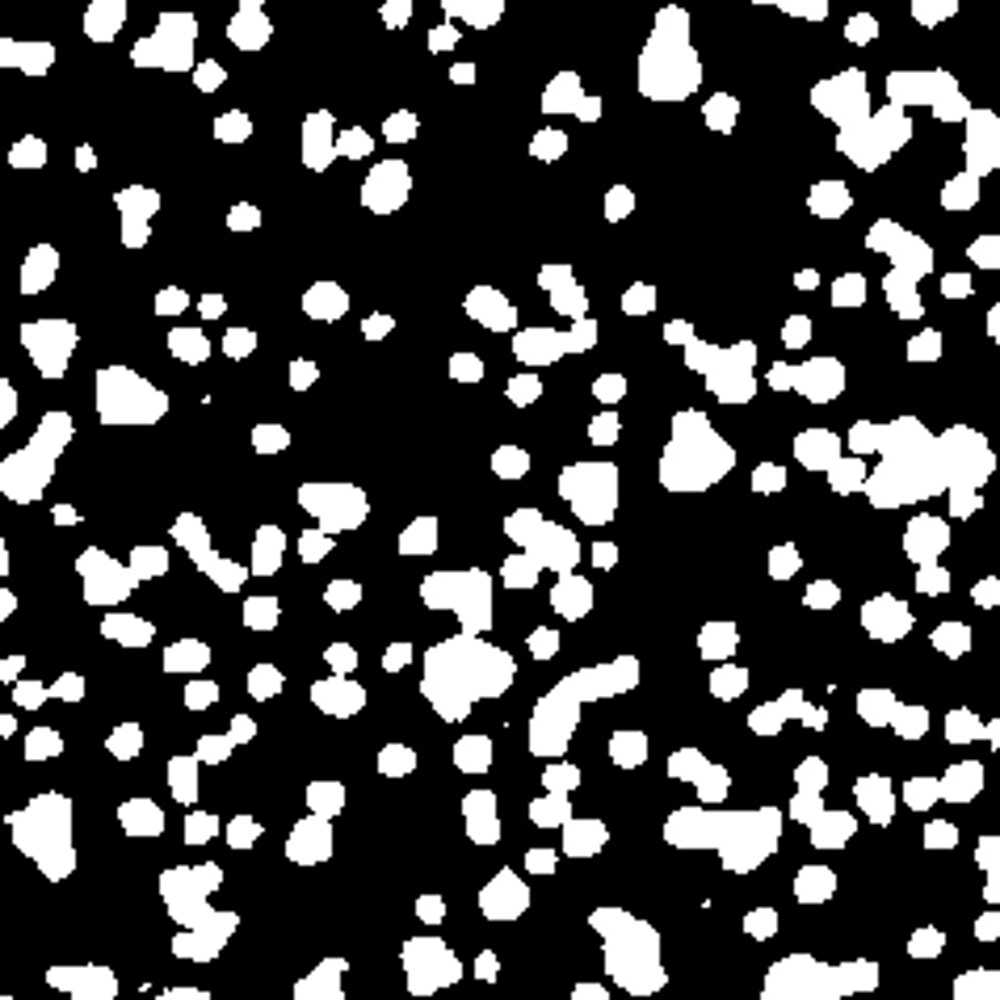

Supplement: Supplementary file 3 — Source Data [file 41467_2023_38178_MOESM3_ESM.zip › Source Data/Fig 3/20.jpg]

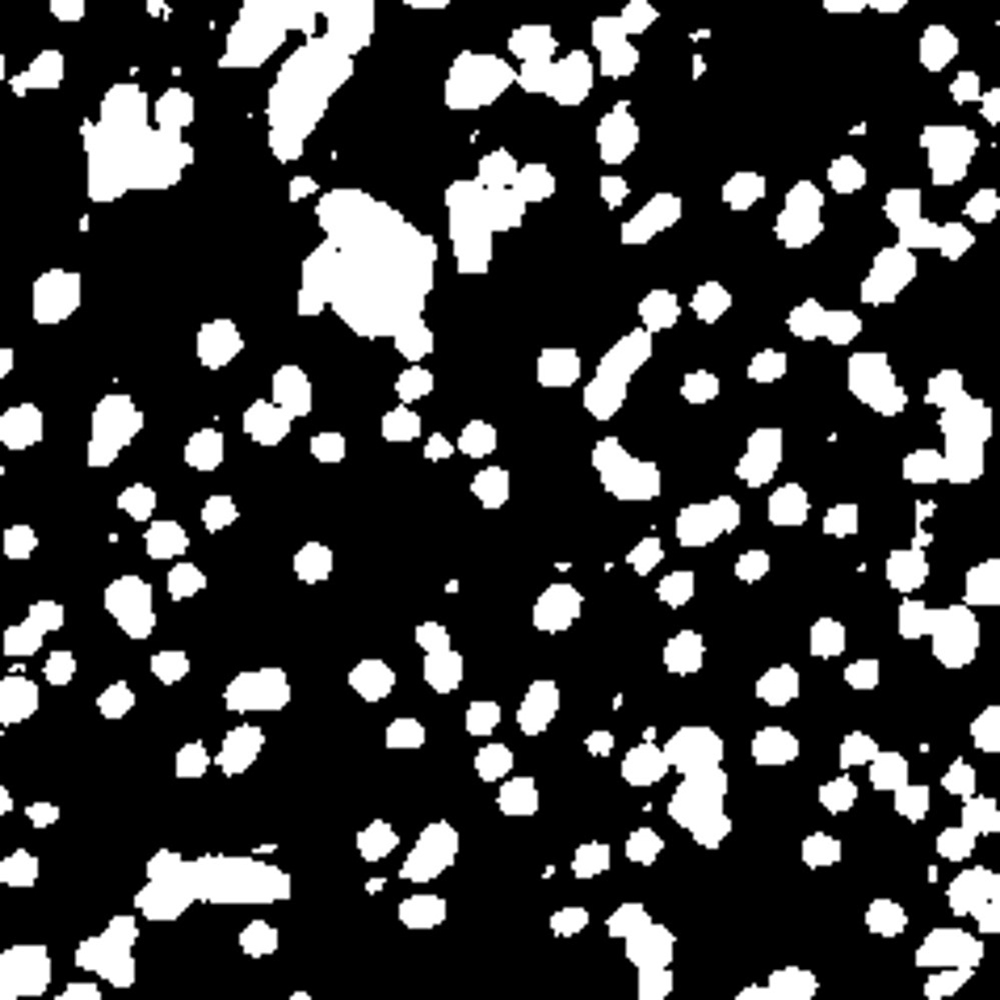

Supplement: Supplementary file 3 — Source Data [file 41467_2023_38178_MOESM3_ESM.zip › Source Data/Fig 3/21.jpg]

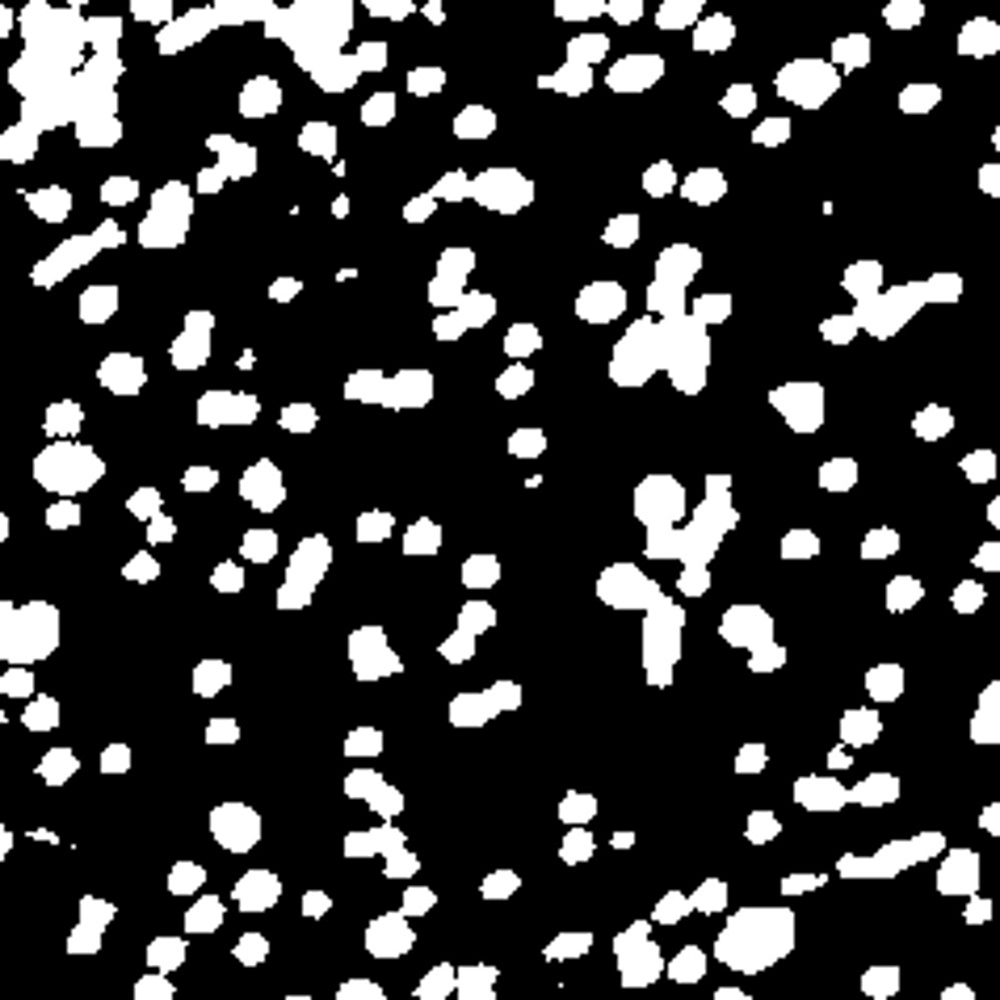

Supplement: Supplementary file 3 — Source Data [file 41467_2023_38178_MOESM3_ESM.zip › Source Data/Fig 3/22.jpg]

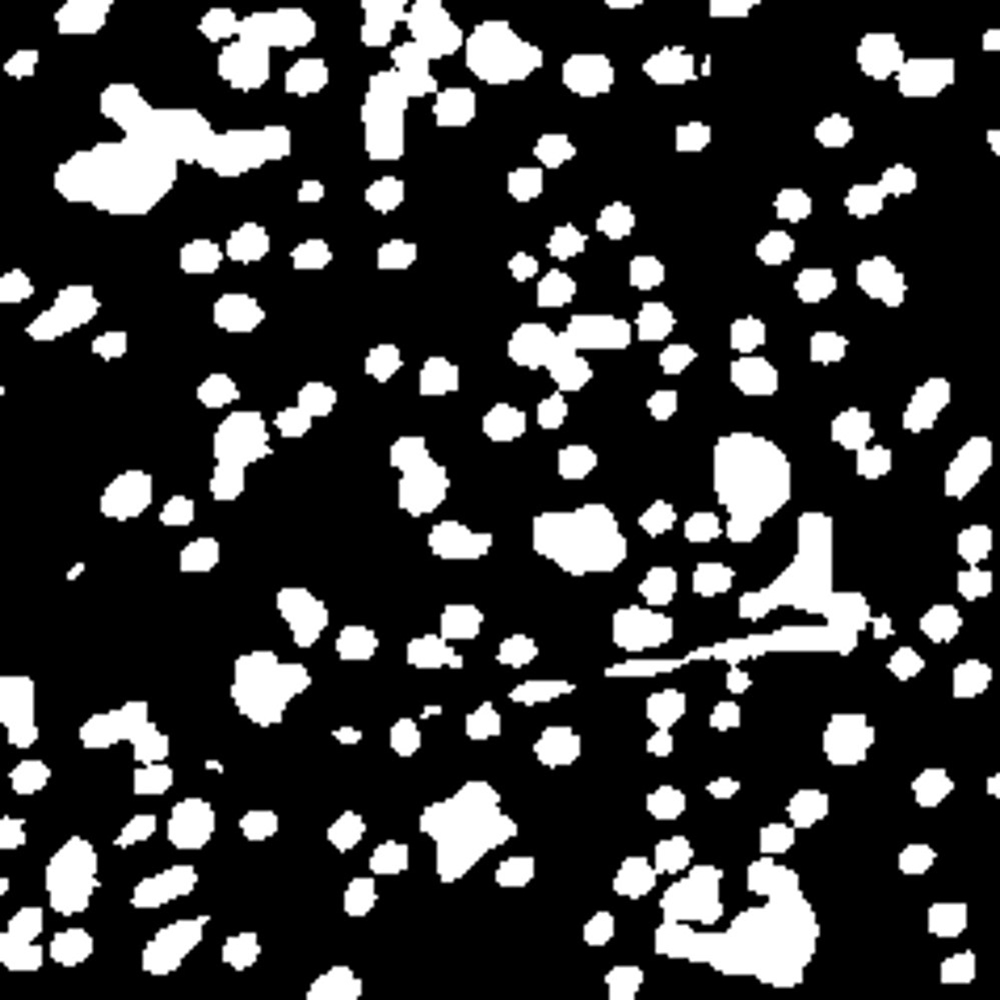

Supplement: Supplementary file 3 — Source Data [file 41467_2023_38178_MOESM3_ESM.zip › Source Data/Fig 3/23.jpg]

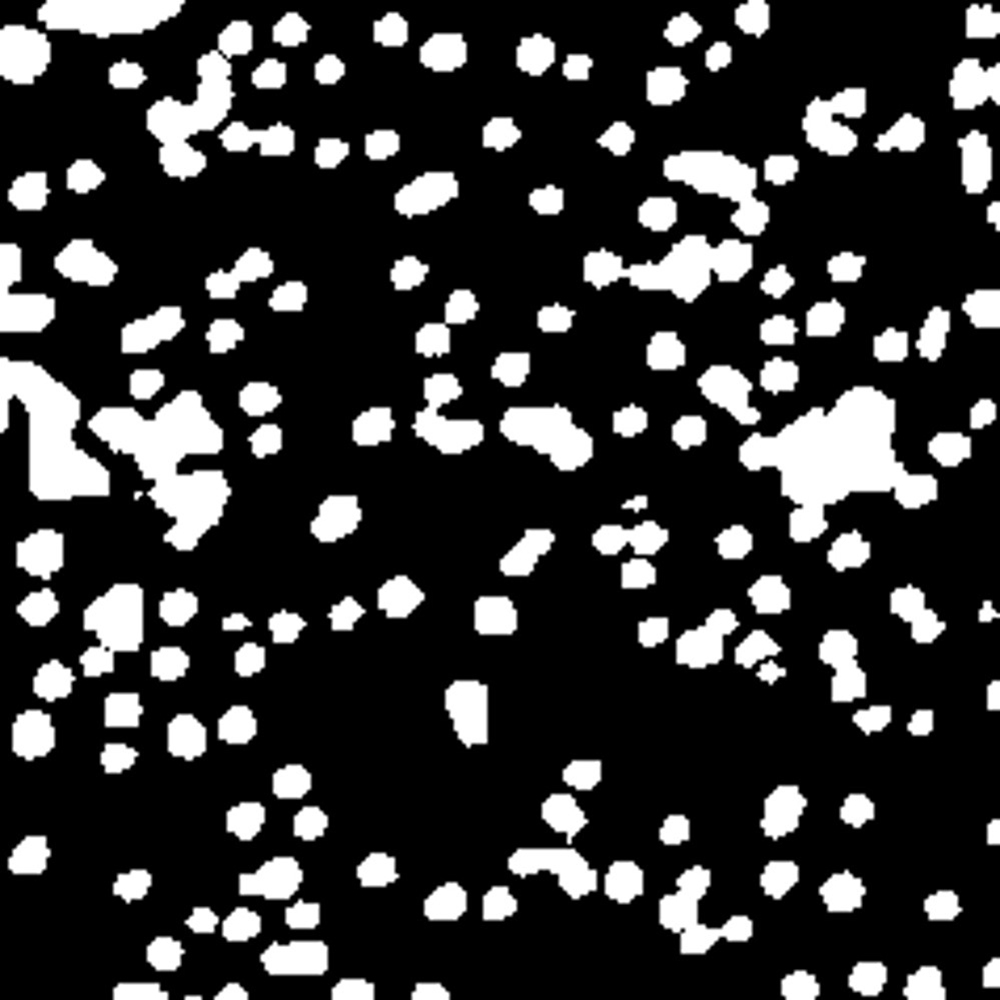

Supplement: Supplementary file 3 — Source Data [file 41467_2023_38178_MOESM3_ESM.zip › Source Data/Fig 3/24.jpg]

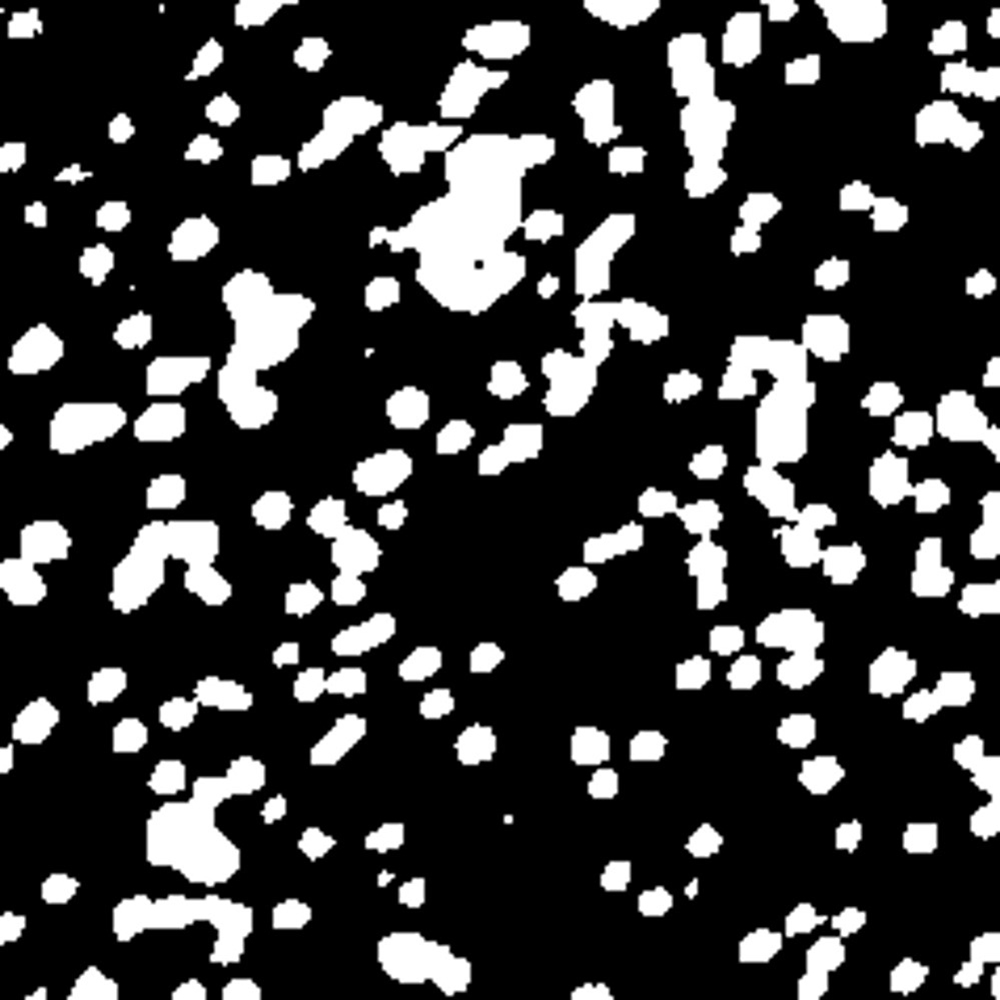

Supplement: Supplementary file 3 — Source Data [file 41467_2023_38178_MOESM3_ESM.zip › Source Data/Fig 3/25.jpg]

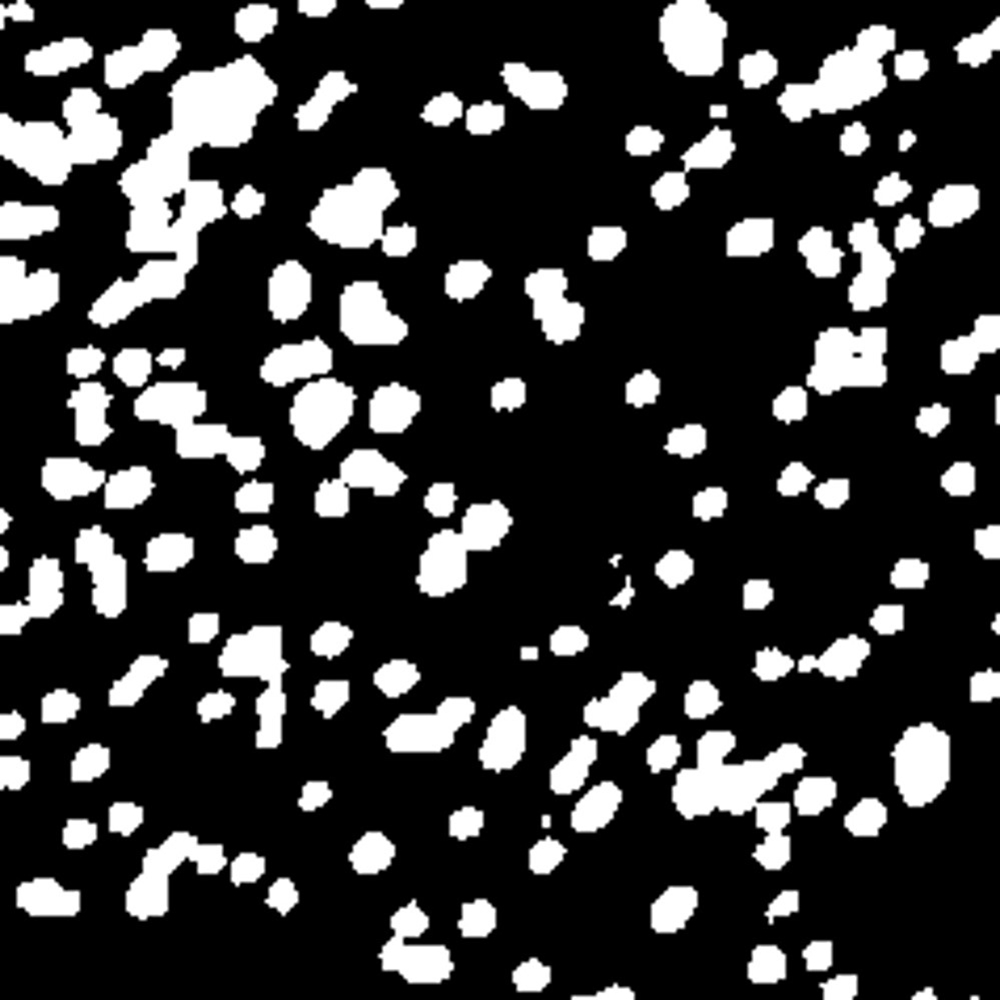

Supplement: Supplementary file 3 — Source Data [file 41467_2023_38178_MOESM3_ESM.zip › Source Data/Fig 3/26.jpg]

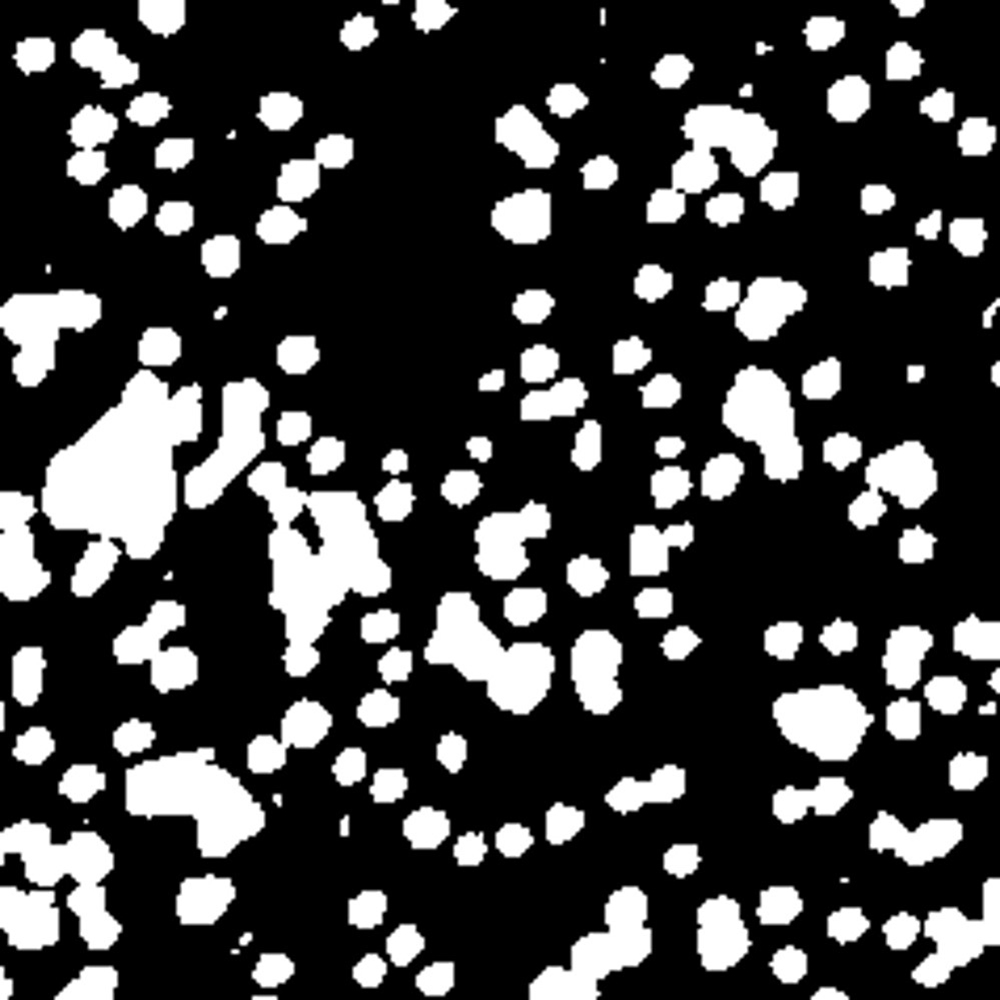

Supplement: Supplementary file 3 — Source Data [file 41467_2023_38178_MOESM3_ESM.zip › Source Data/Fig 3/27.jpg]

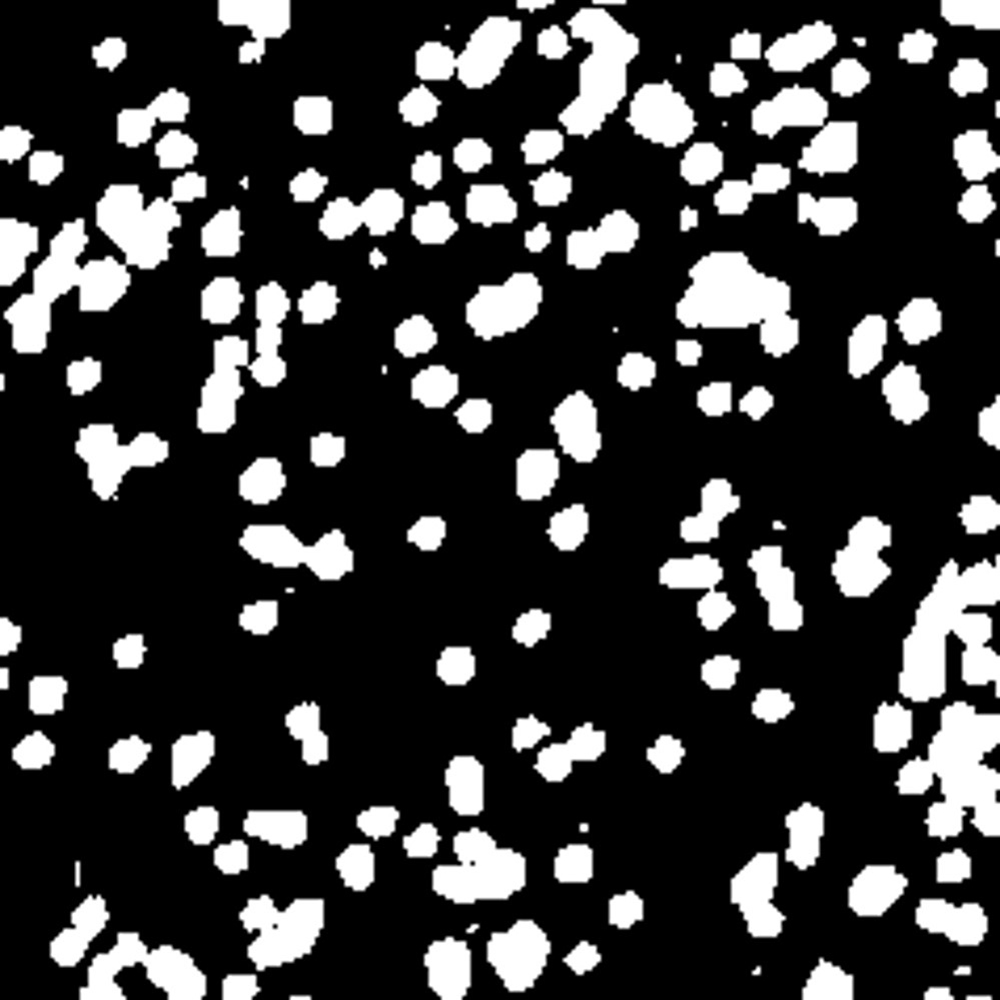

Supplement: Supplementary file 3 — Source Data [file 41467_2023_38178_MOESM3_ESM.zip › Source Data/Fig 3/28.jpg]

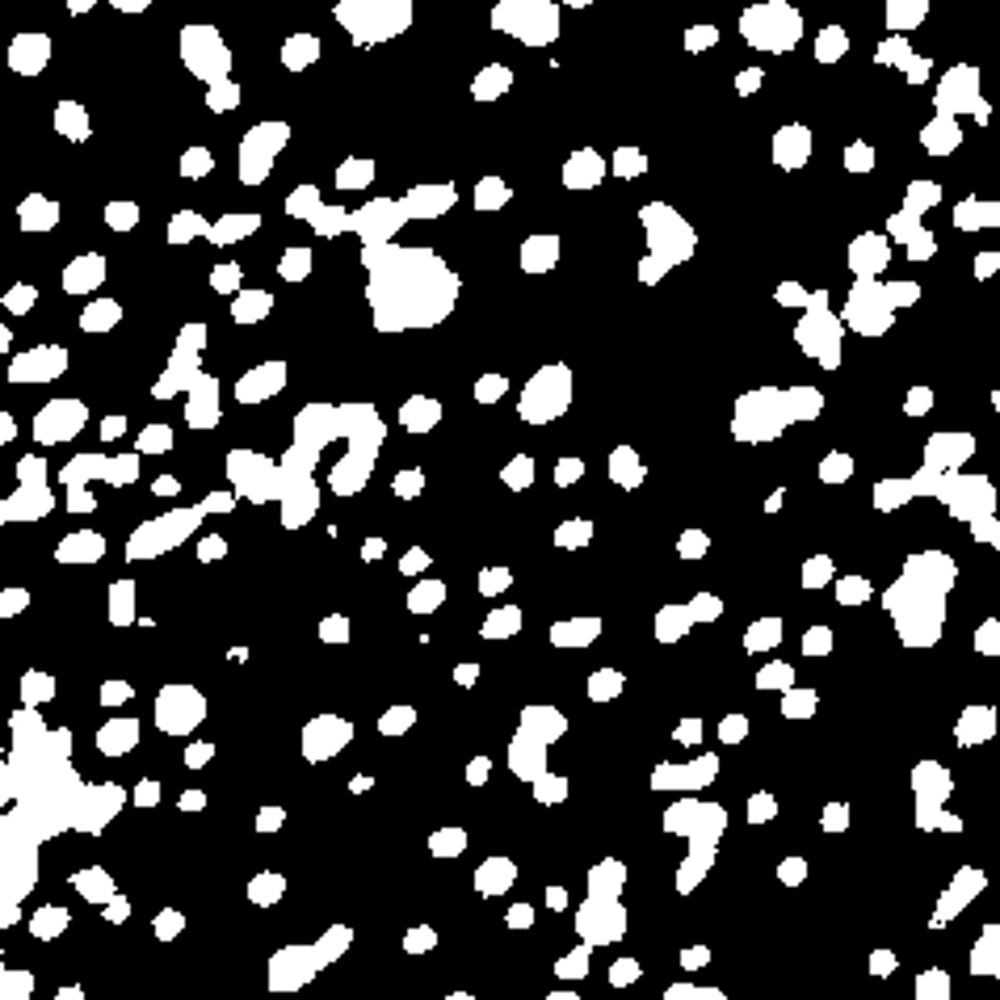

Supplement: Supplementary file 3 — Source Data [file 41467_2023_38178_MOESM3_ESM.zip › Source Data/Fig 3/29.jpg]

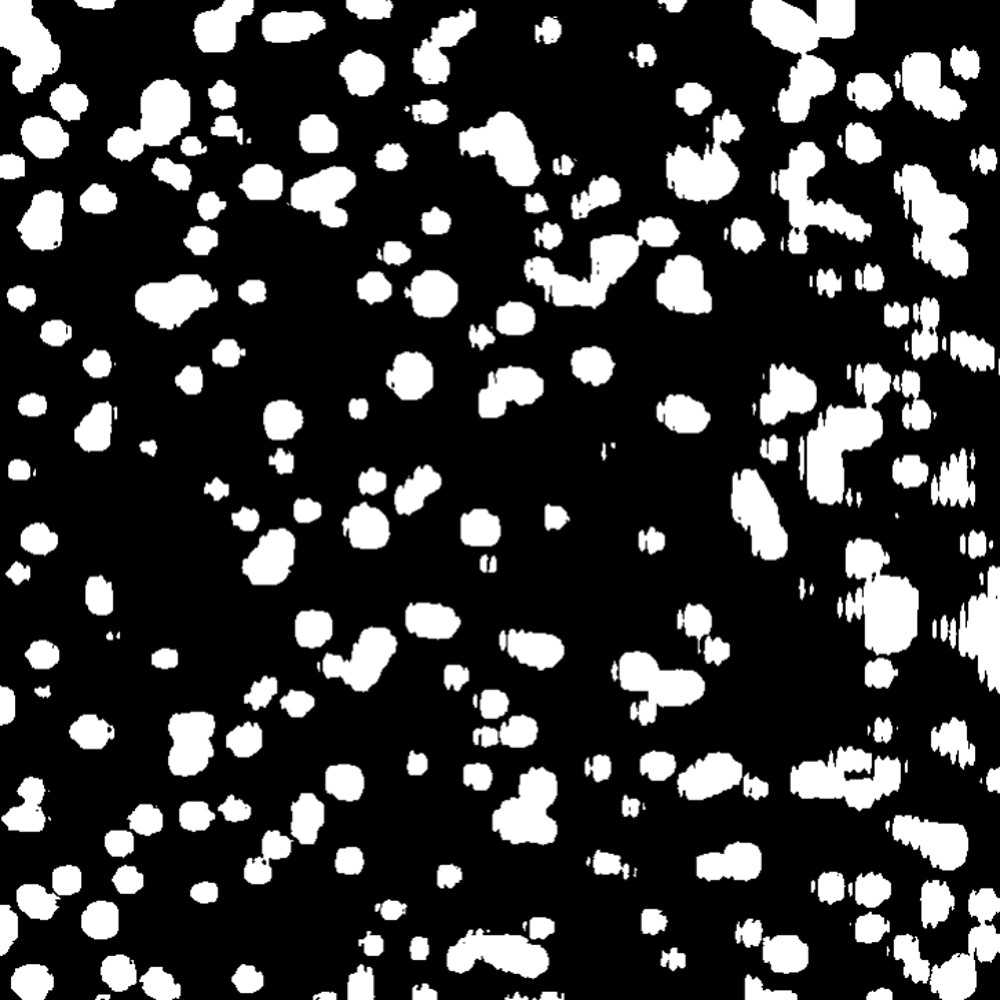

Supplement: Supplementary file 3 — Source Data [file 41467_2023_38178_MOESM3_ESM.zip › Source Data/Fig 3/3.jpg]

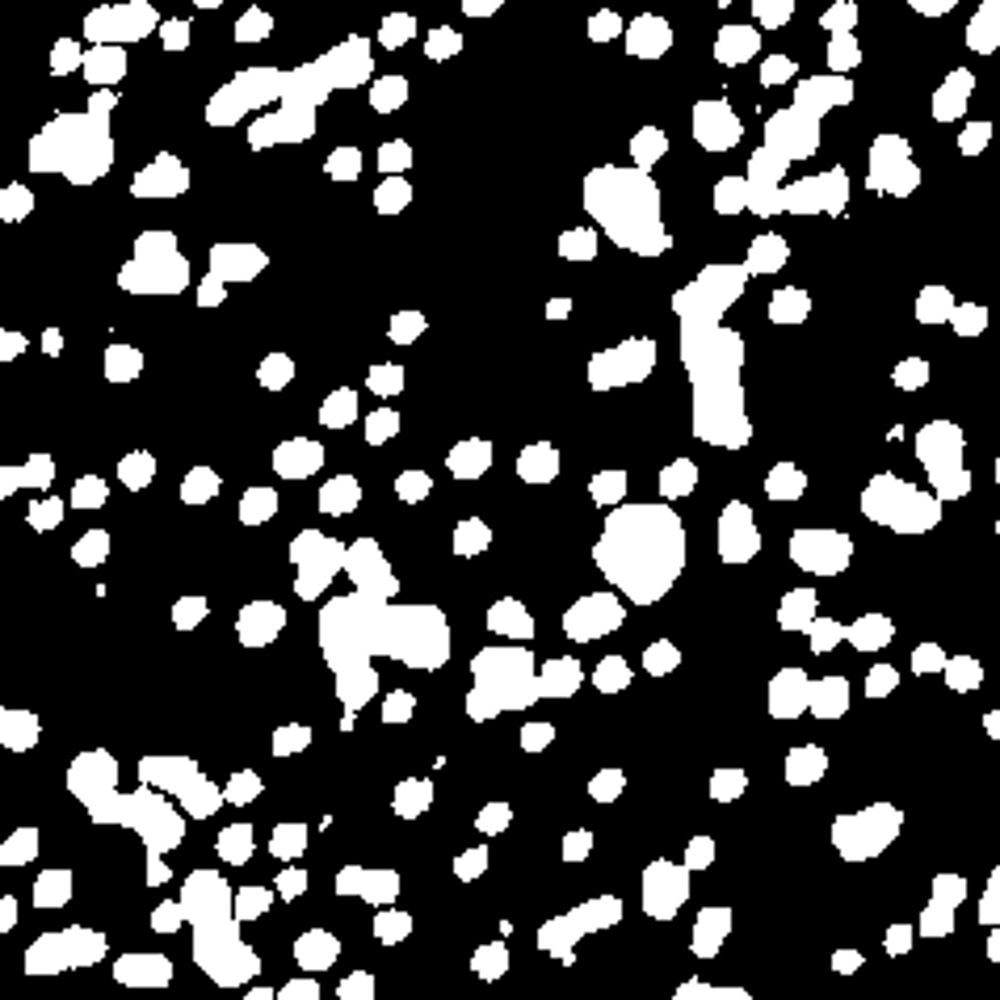

Supplement: Supplementary file 3 — Source Data [file 41467_2023_38178_MOESM3_ESM.zip › Source Data/Fig 3/30.jpg]

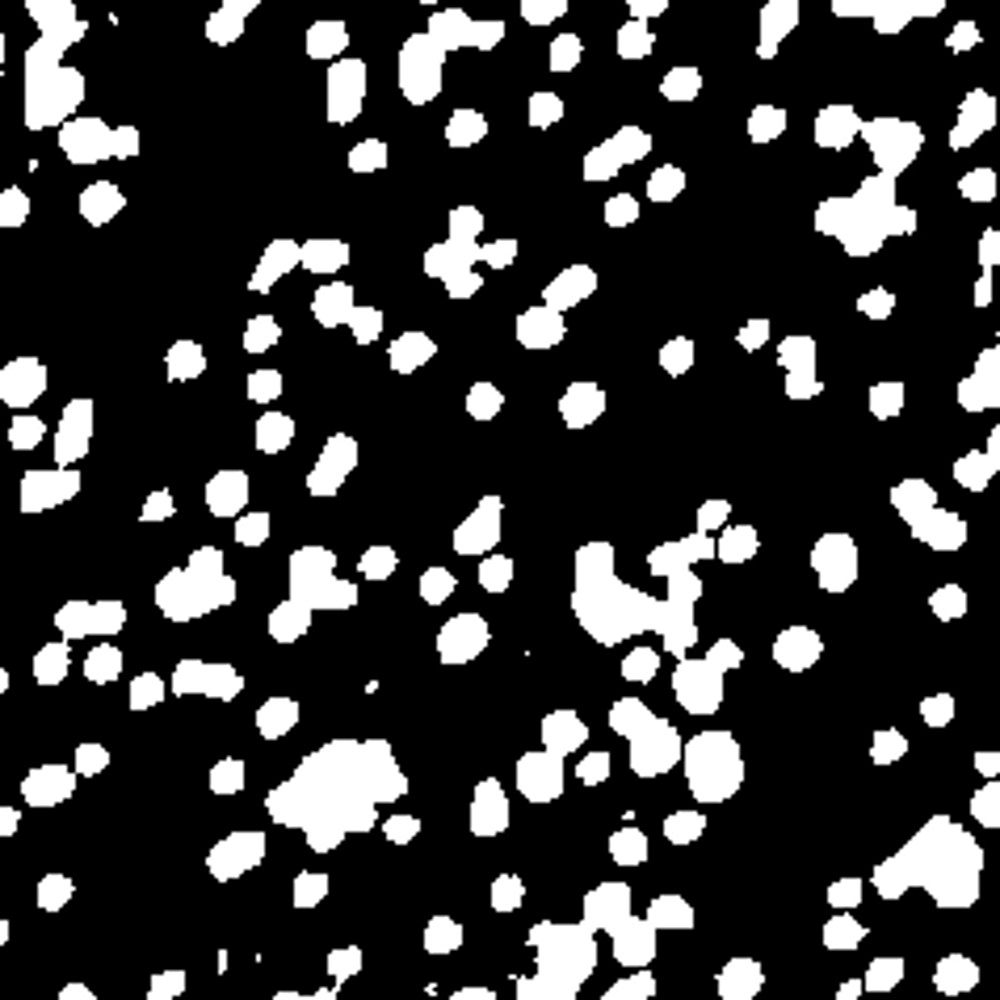

Supplement: Supplementary file 3 — Source Data [file 41467_2023_38178_MOESM3_ESM.zip › Source Data/Fig 3/31.jpg]

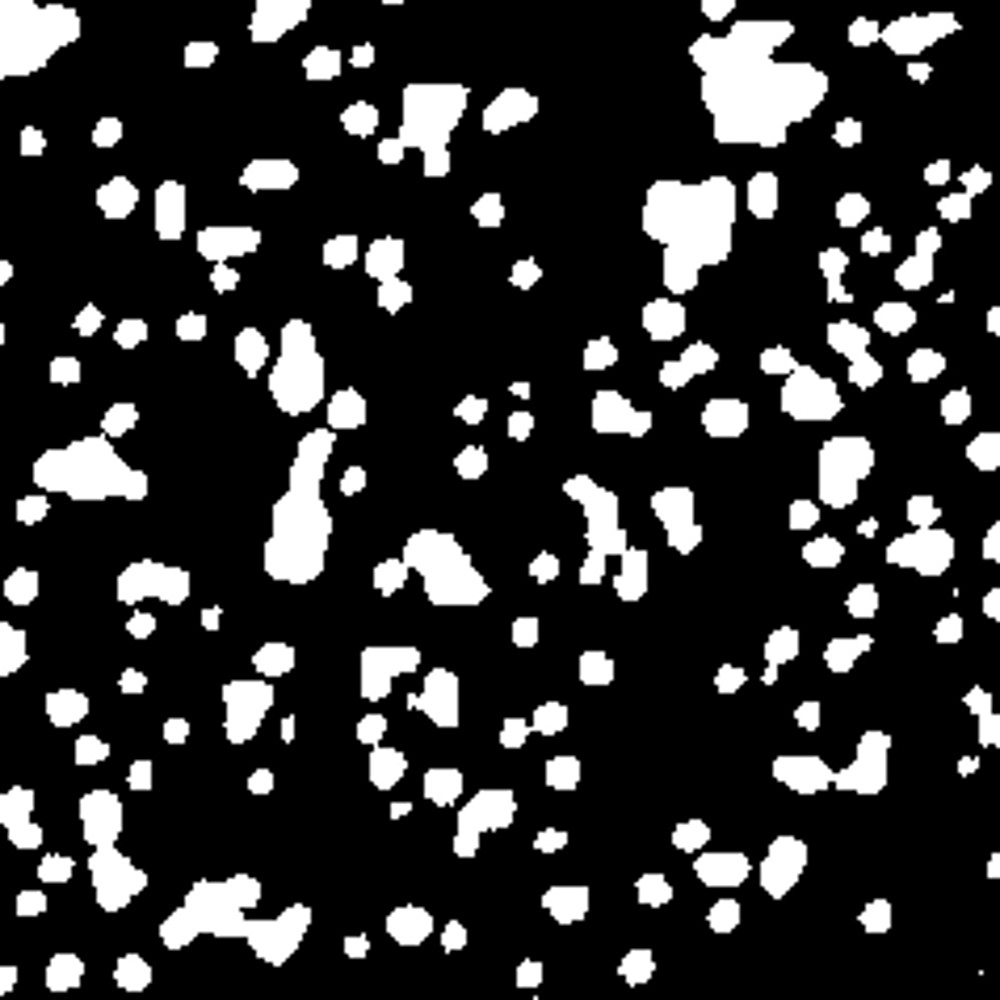

Supplement: Supplementary file 3 — Source Data [file 41467_2023_38178_MOESM3_ESM.zip › Source Data/Fig 3/32.jpg]

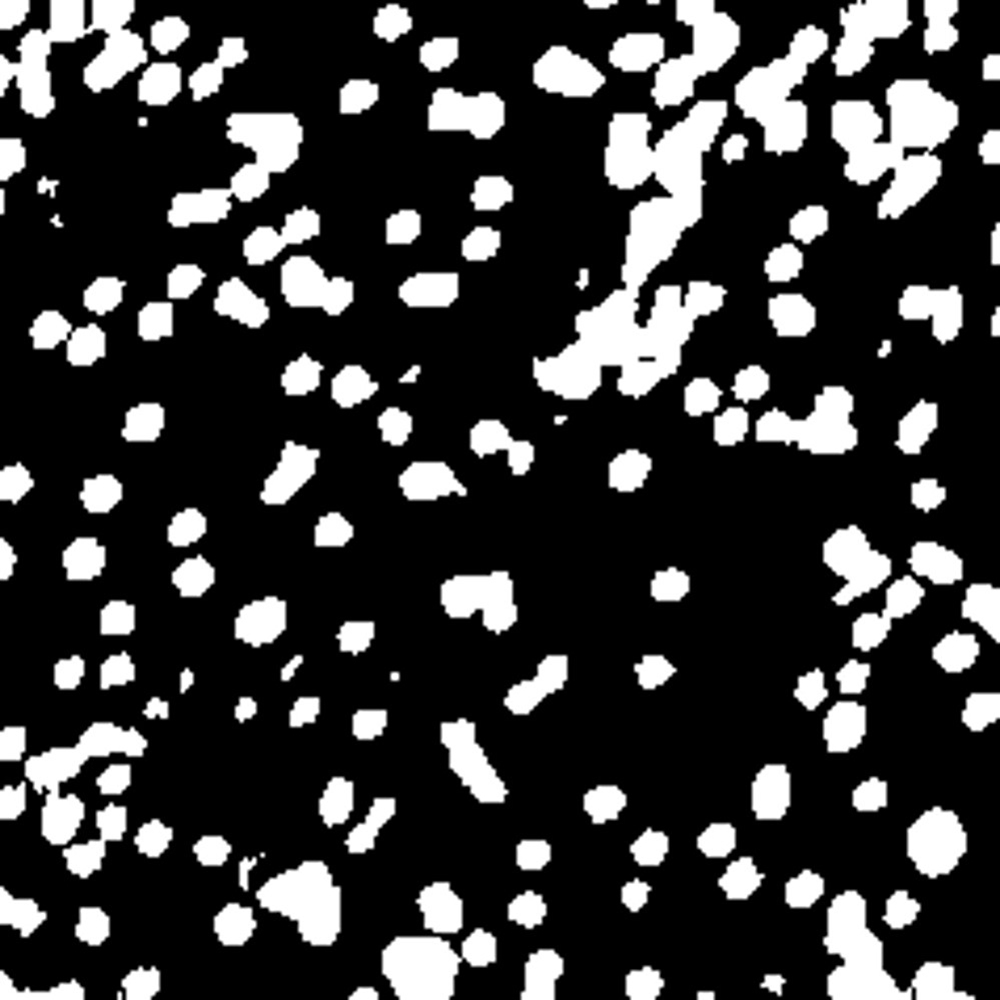

Supplement: Supplementary file 3 — Source Data [file 41467_2023_38178_MOESM3_ESM.zip › Source Data/Fig 3/33.jpg]

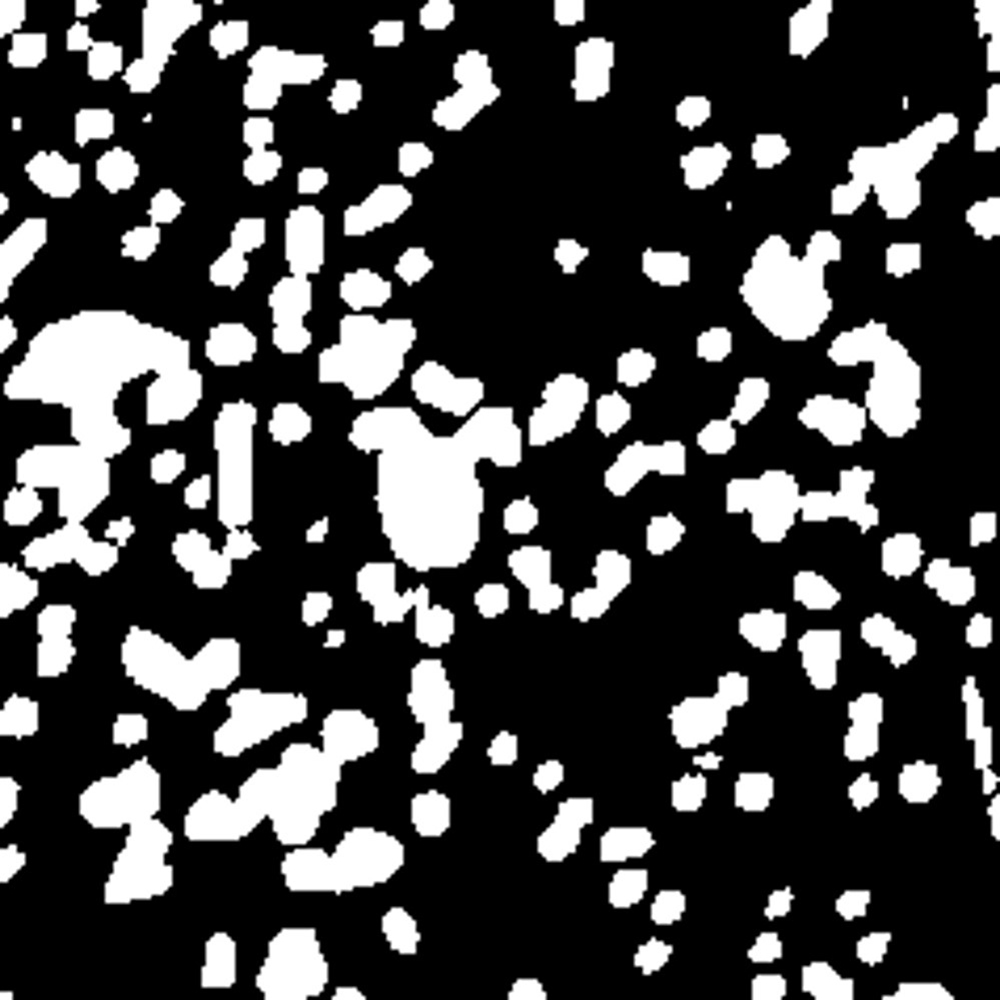

Supplement: Supplementary file 3 — Source Data [file 41467_2023_38178_MOESM3_ESM.zip › Source Data/Fig 3/34.jpg]

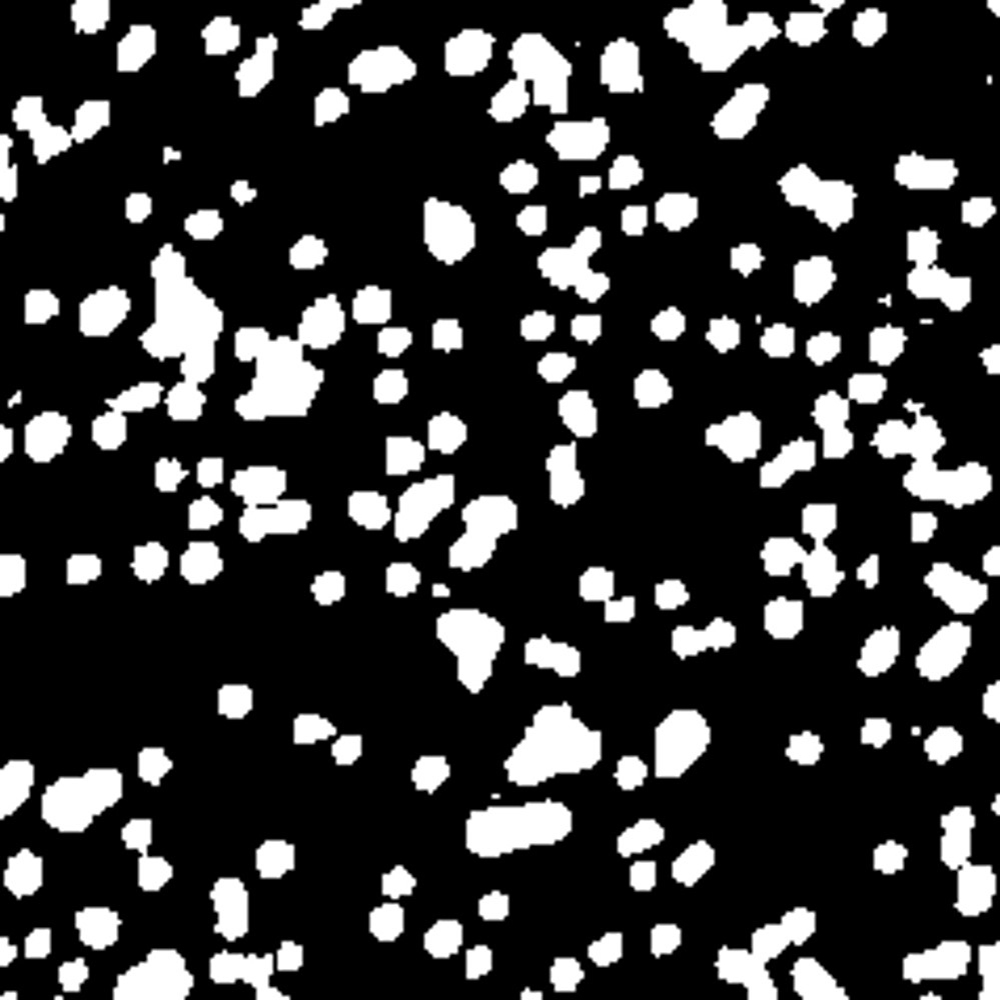

Supplement: Supplementary file 3 — Source Data [file 41467_2023_38178_MOESM3_ESM.zip › Source Data/Fig 3/35.jpg]

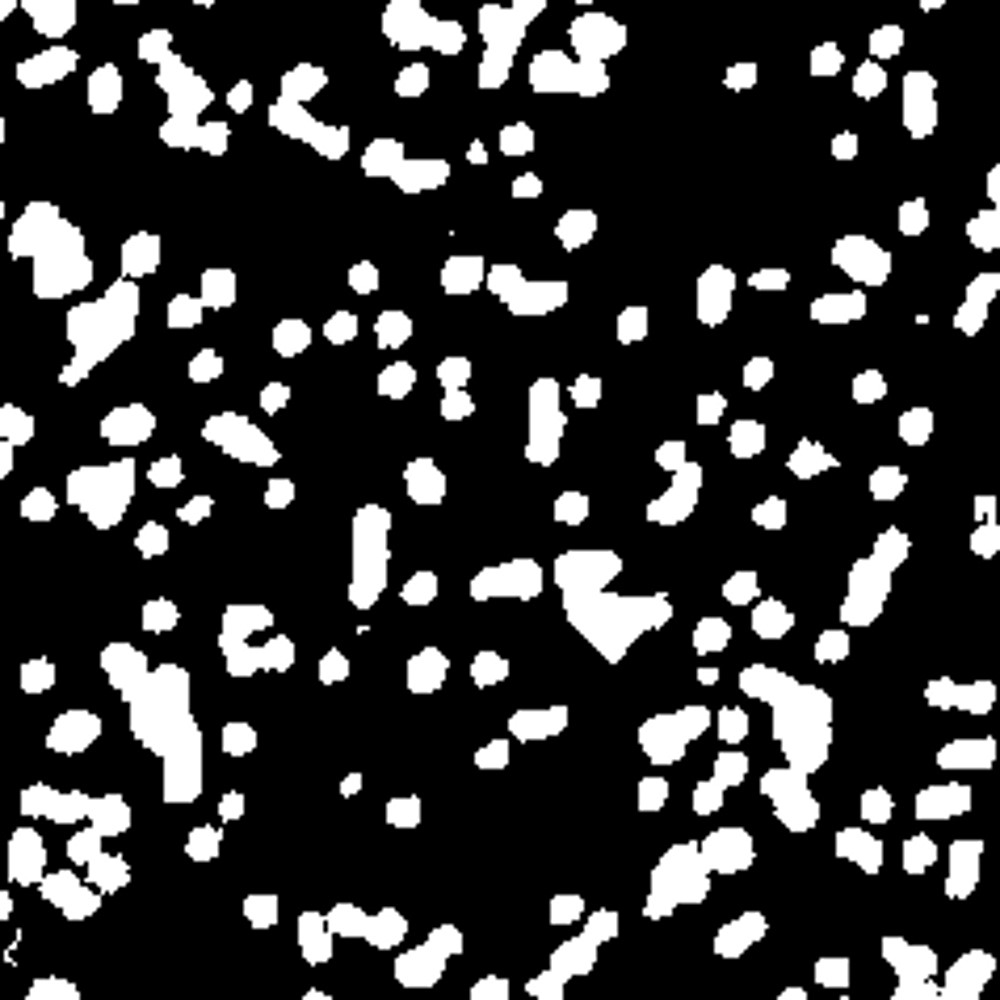

Supplement: Supplementary file 3 — Source Data [file 41467_2023_38178_MOESM3_ESM.zip › Source Data/Fig 3/36.jpg]

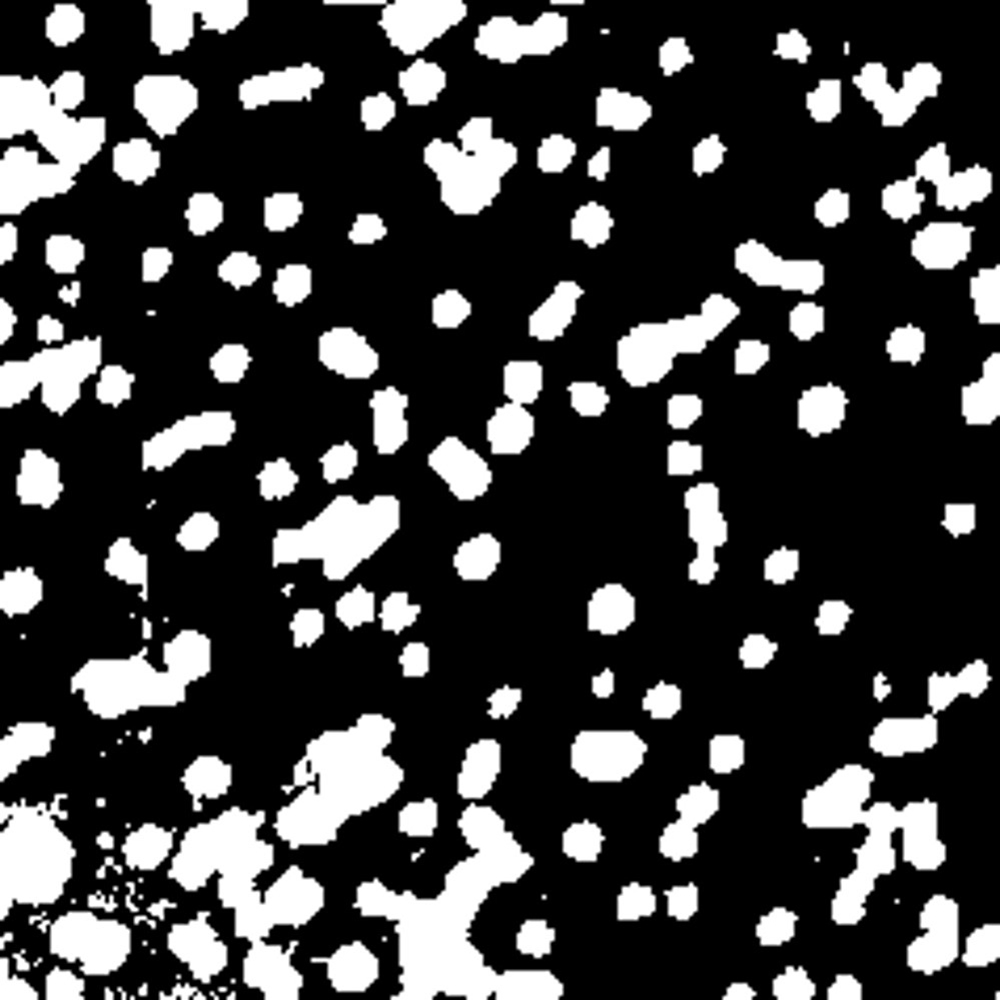

Supplement: Supplementary file 3 — Source Data [file 41467_2023_38178_MOESM3_ESM.zip › Source Data/Fig 3/37.jpg]

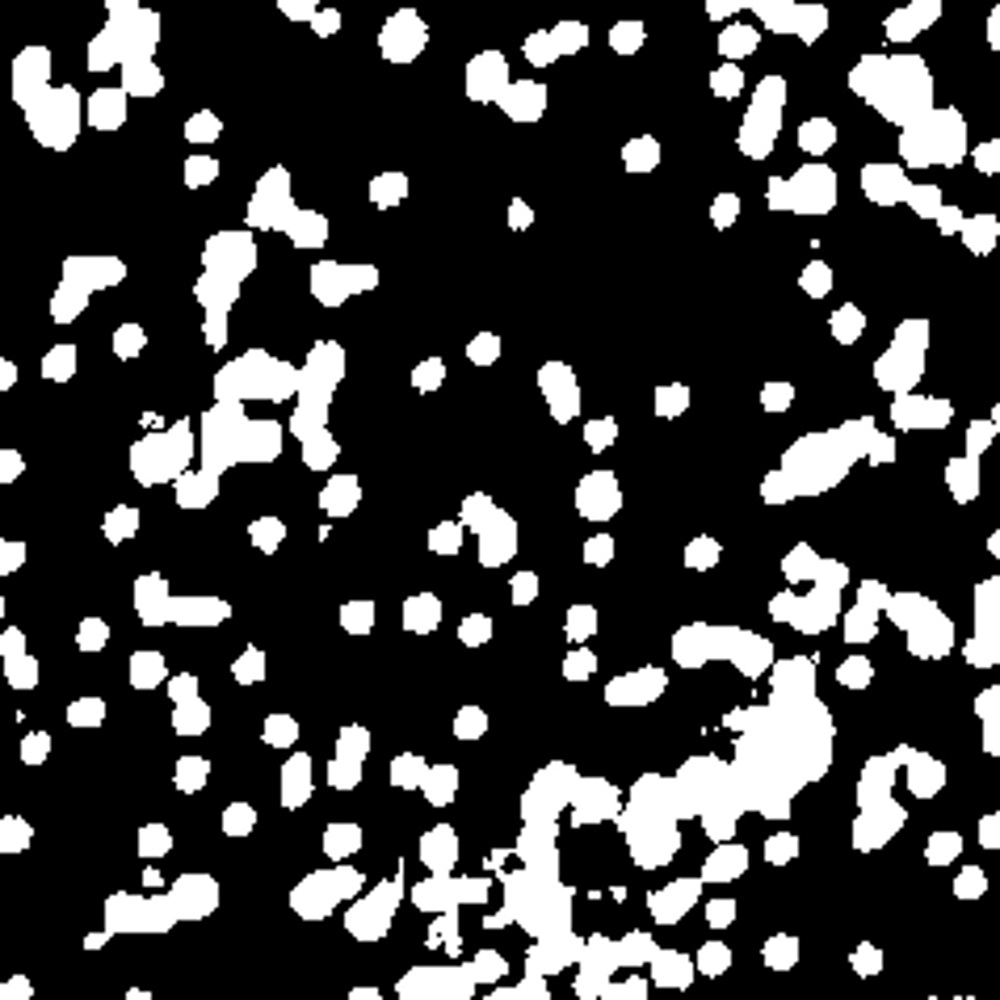

Supplement: Supplementary file 3 — Source Data [file 41467_2023_38178_MOESM3_ESM.zip › Source Data/Fig 3/38.jpg]

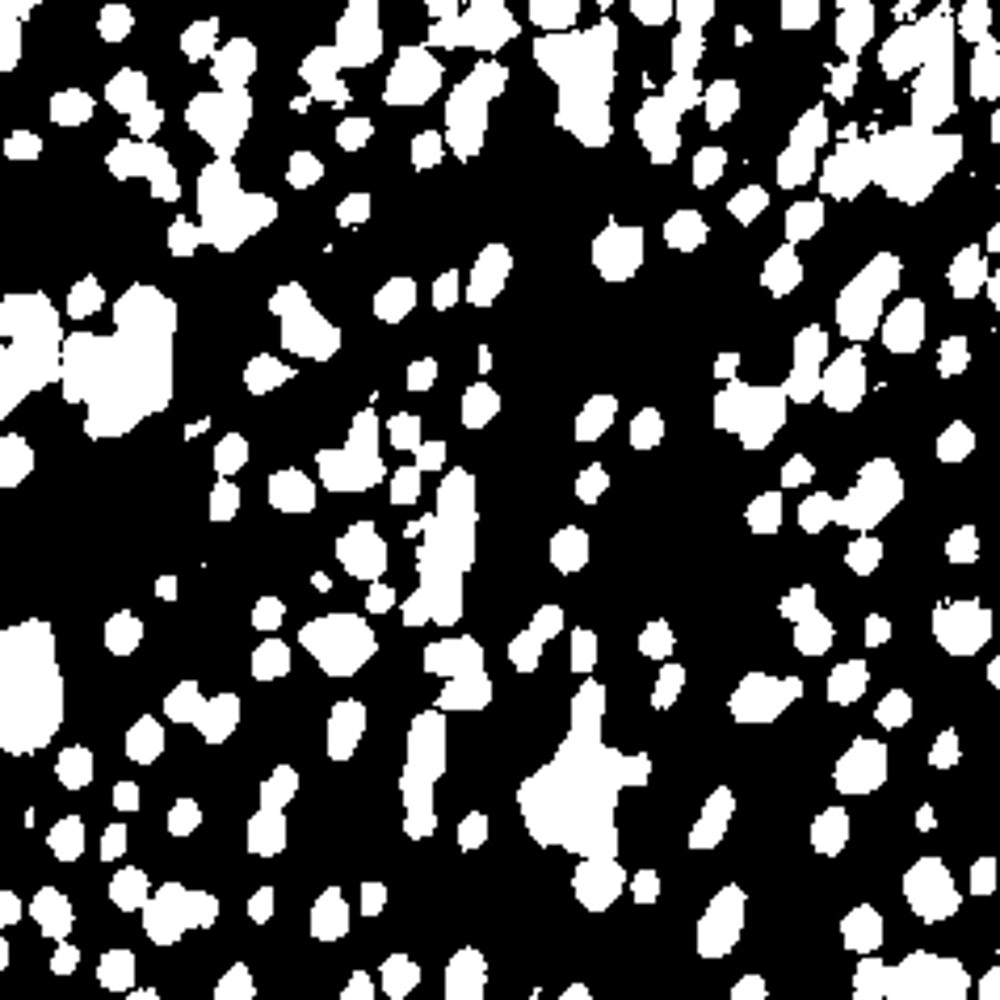

Supplement: Supplementary file 3 — Source Data [file 41467_2023_38178_MOESM3_ESM.zip › Source Data/Fig 3/39.jpg]

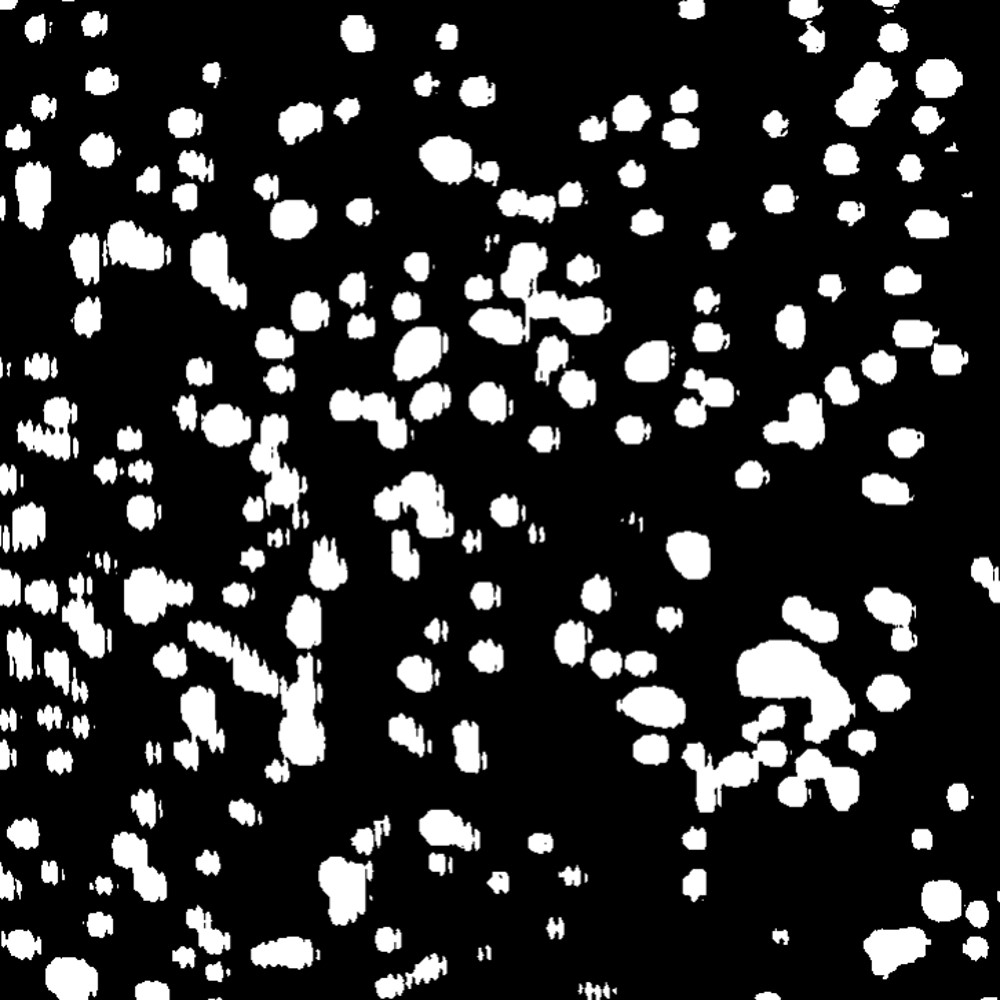

Supplement: Supplementary file 3 — Source Data [file 41467_2023_38178_MOESM3_ESM.zip › Source Data/Fig 3/4.jpg]

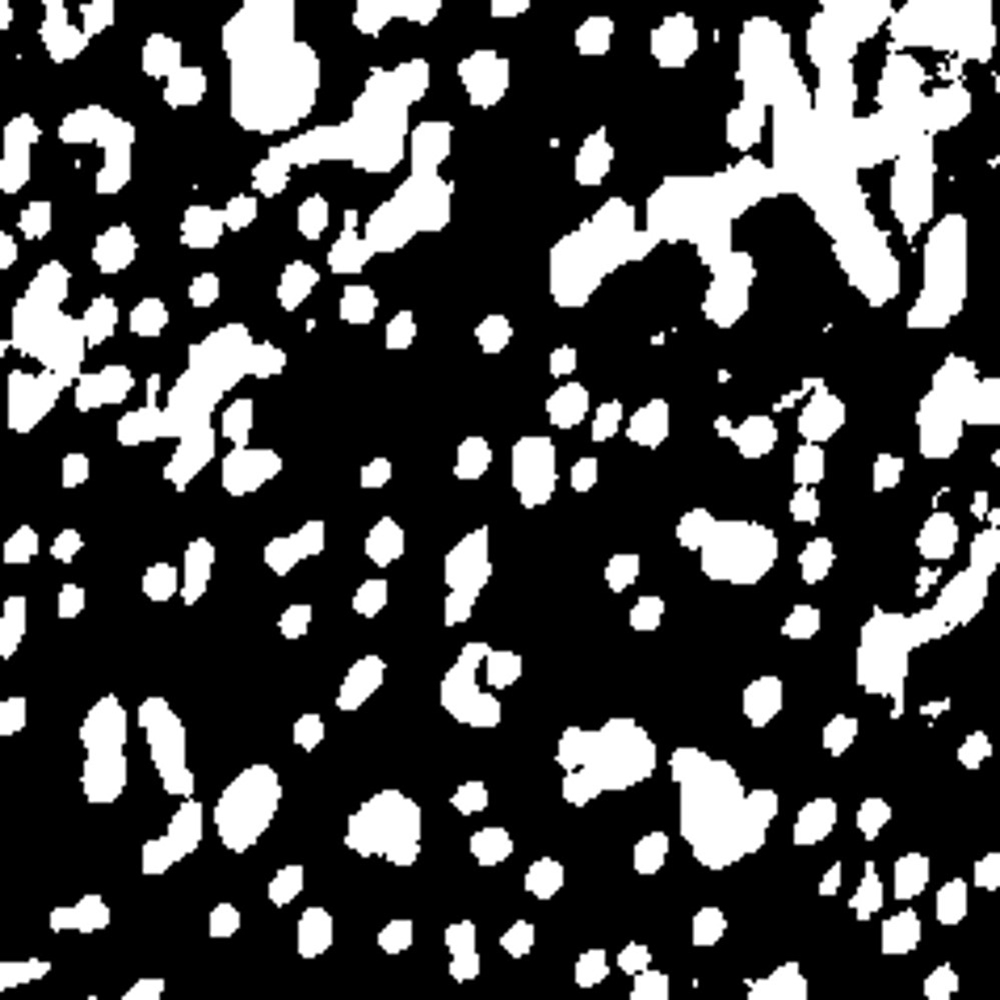

Supplement: Supplementary file 3 — Source Data [file 41467_2023_38178_MOESM3_ESM.zip › Source Data/Fig 3/40.jpg]

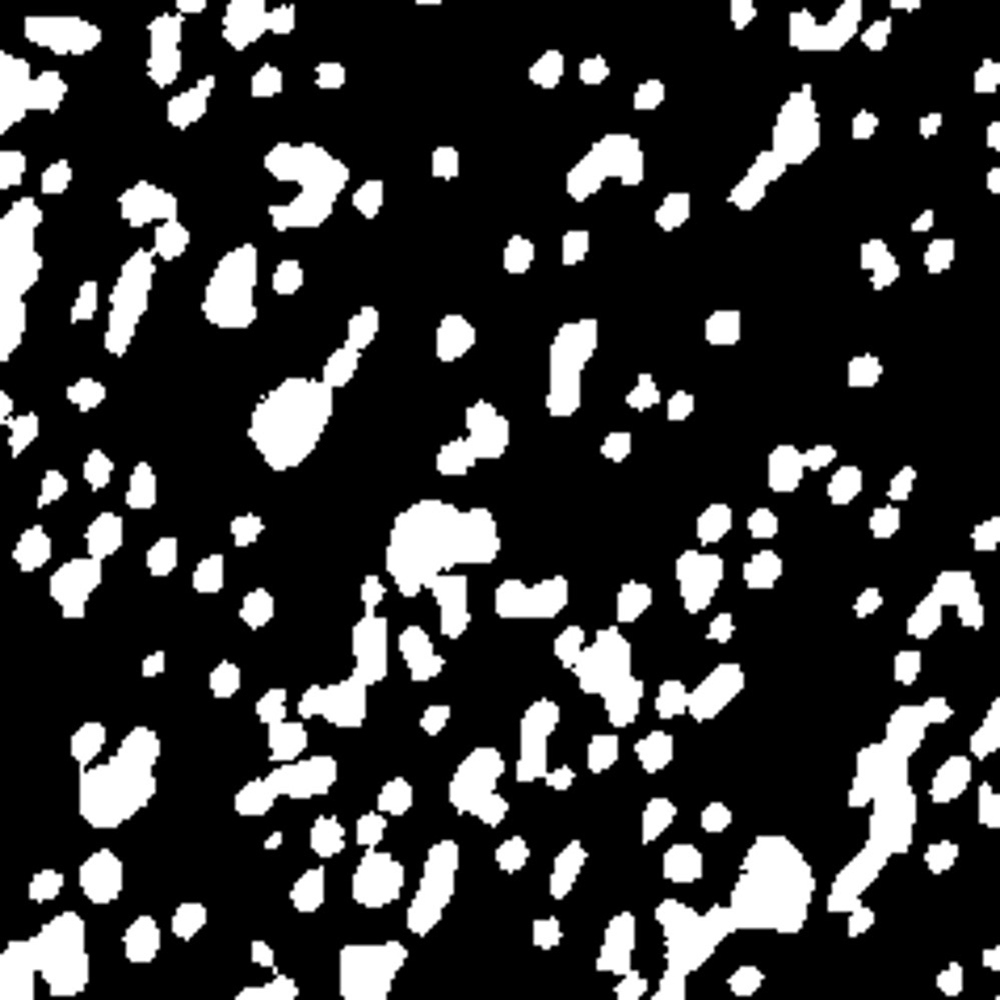

Supplement: Supplementary file 3 — Source Data [file 41467_2023_38178_MOESM3_ESM.zip › Source Data/Fig 3/41.jpg]

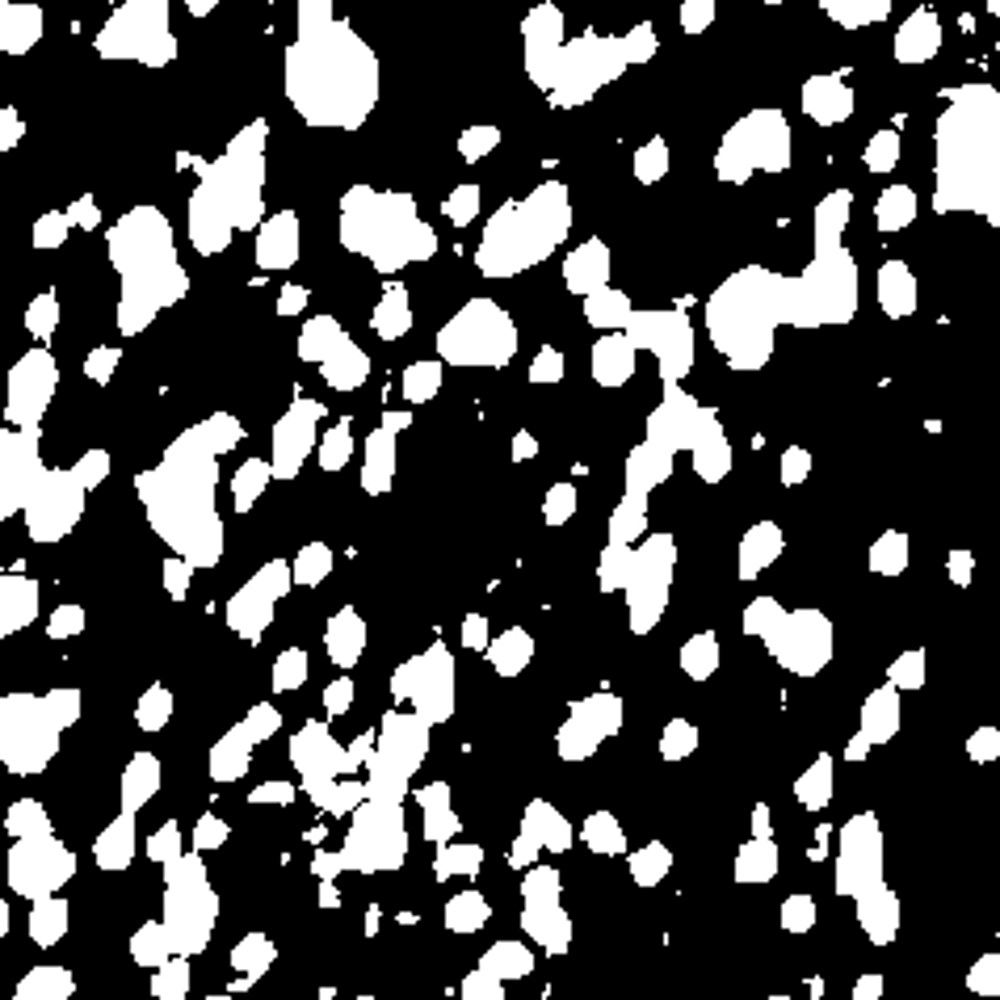

Supplement: Supplementary file 3 — Source Data [file 41467_2023_38178_MOESM3_ESM.zip › Source Data/Fig 3/42.jpg]

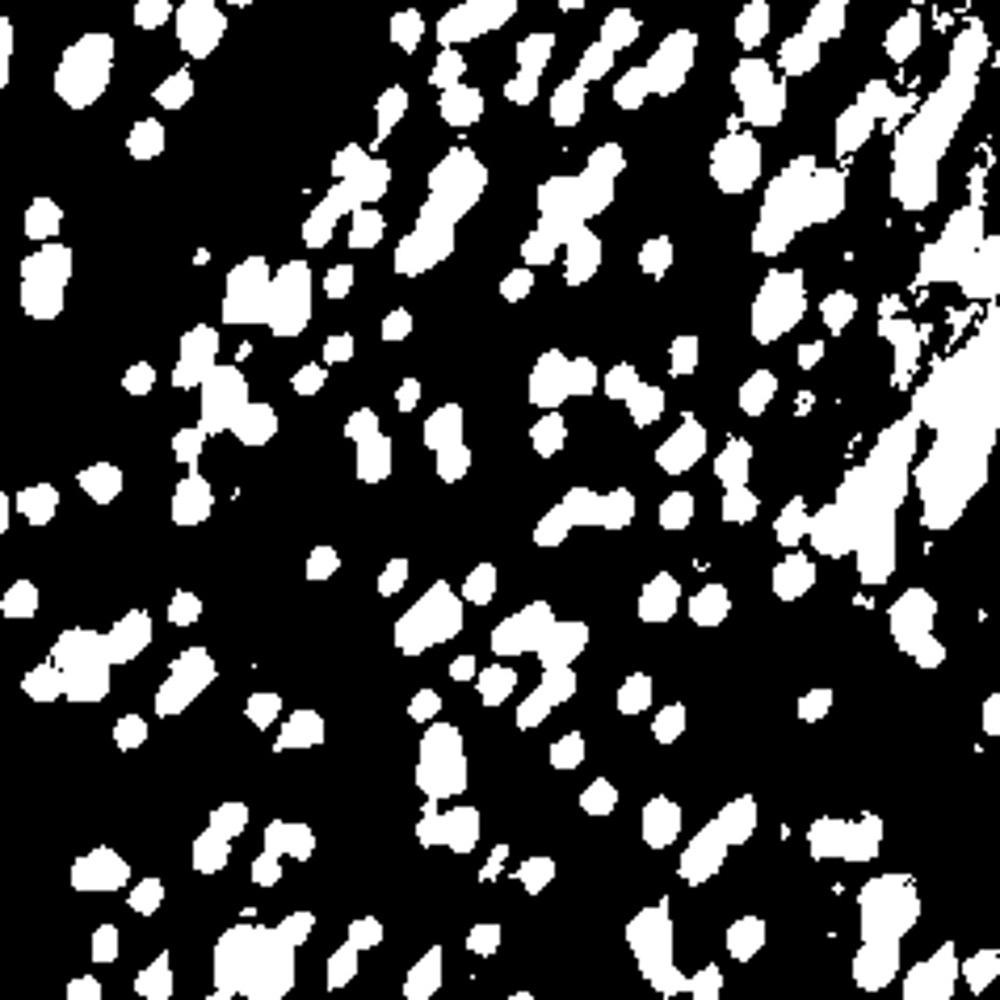

Supplement: Supplementary file 3 — Source Data [file 41467_2023_38178_MOESM3_ESM.zip › Source Data/Fig 3/43.jpg]

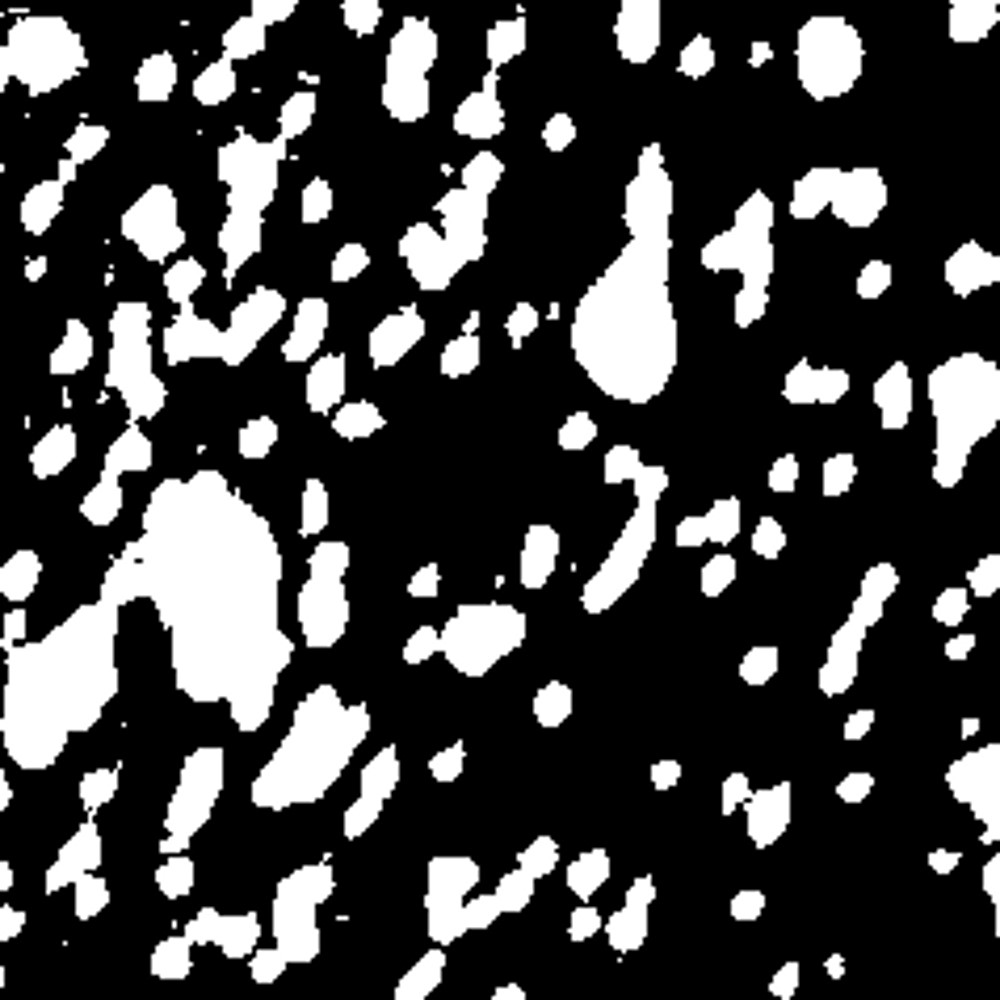

Supplement: Supplementary file 3 — Source Data [file 41467_2023_38178_MOESM3_ESM.zip › Source Data/Fig 3/44.jpg]

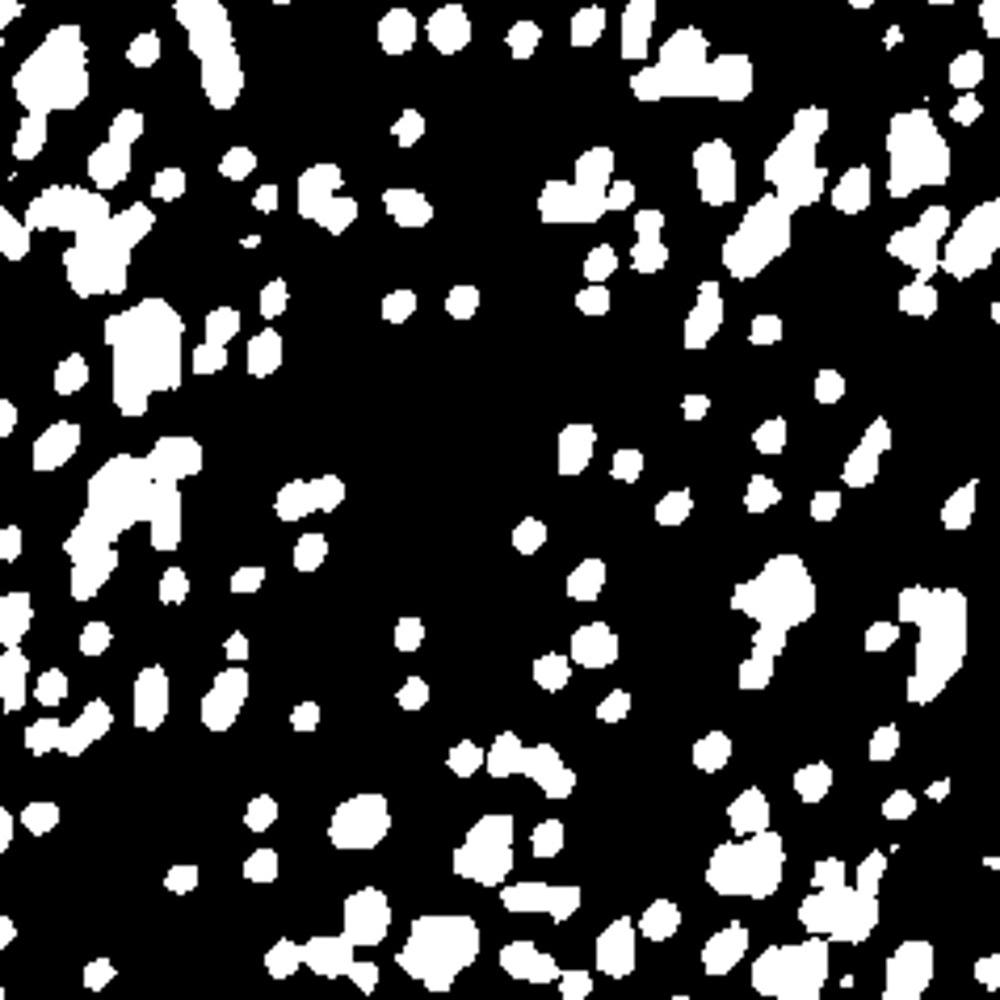

Supplement: Supplementary file 3 — Source Data [file 41467_2023_38178_MOESM3_ESM.zip › Source Data/Fig 3/45.jpg]

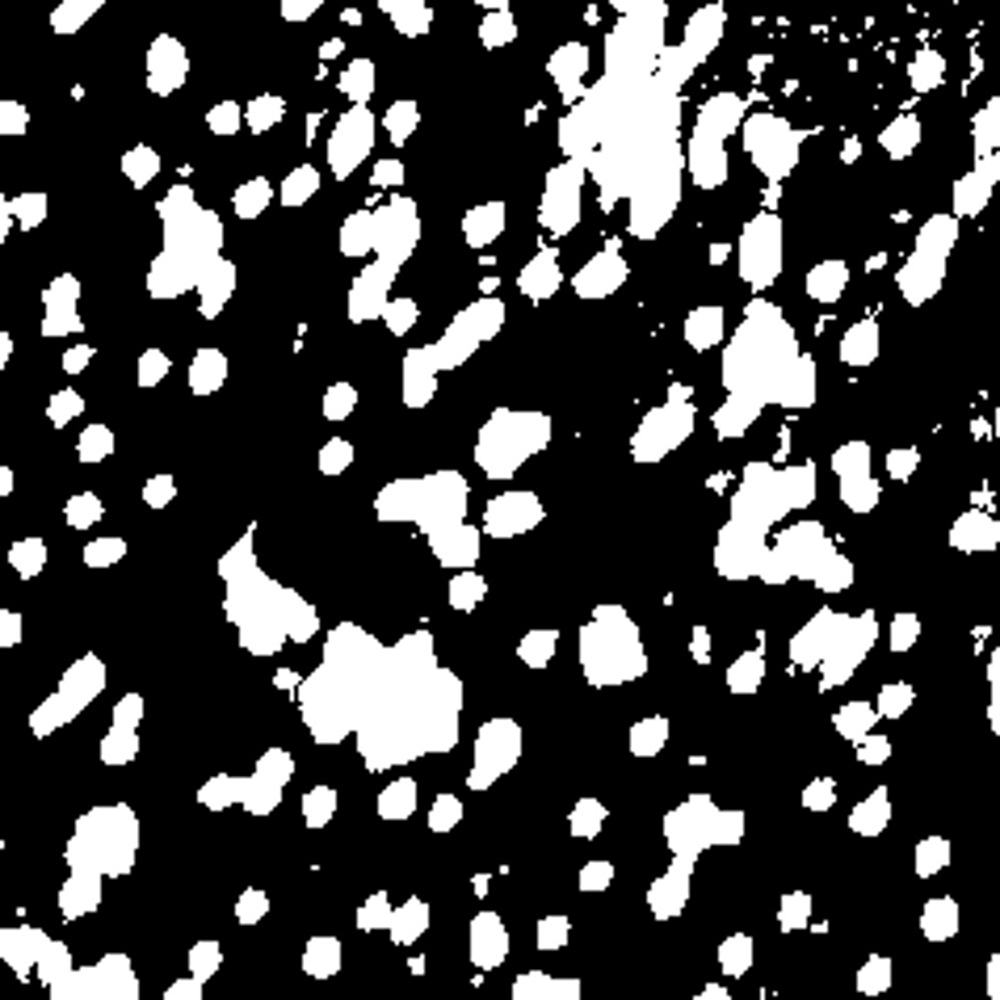

Supplement: Supplementary file 3 — Source Data [file 41467_2023_38178_MOESM3_ESM.zip › Source Data/Fig 3/46.jpg]

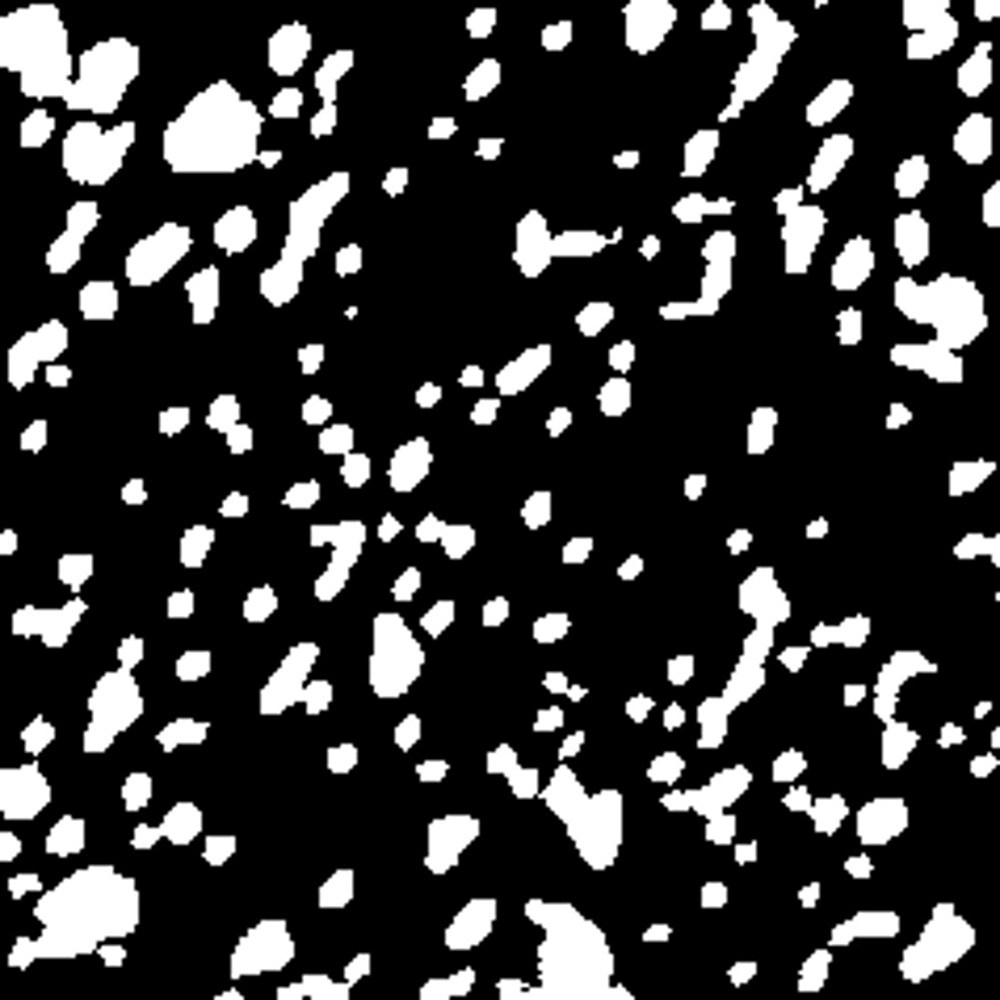

Supplement: Supplementary file 3 — Source Data [file 41467_2023_38178_MOESM3_ESM.zip › Source Data/Fig 3/47.jpg]

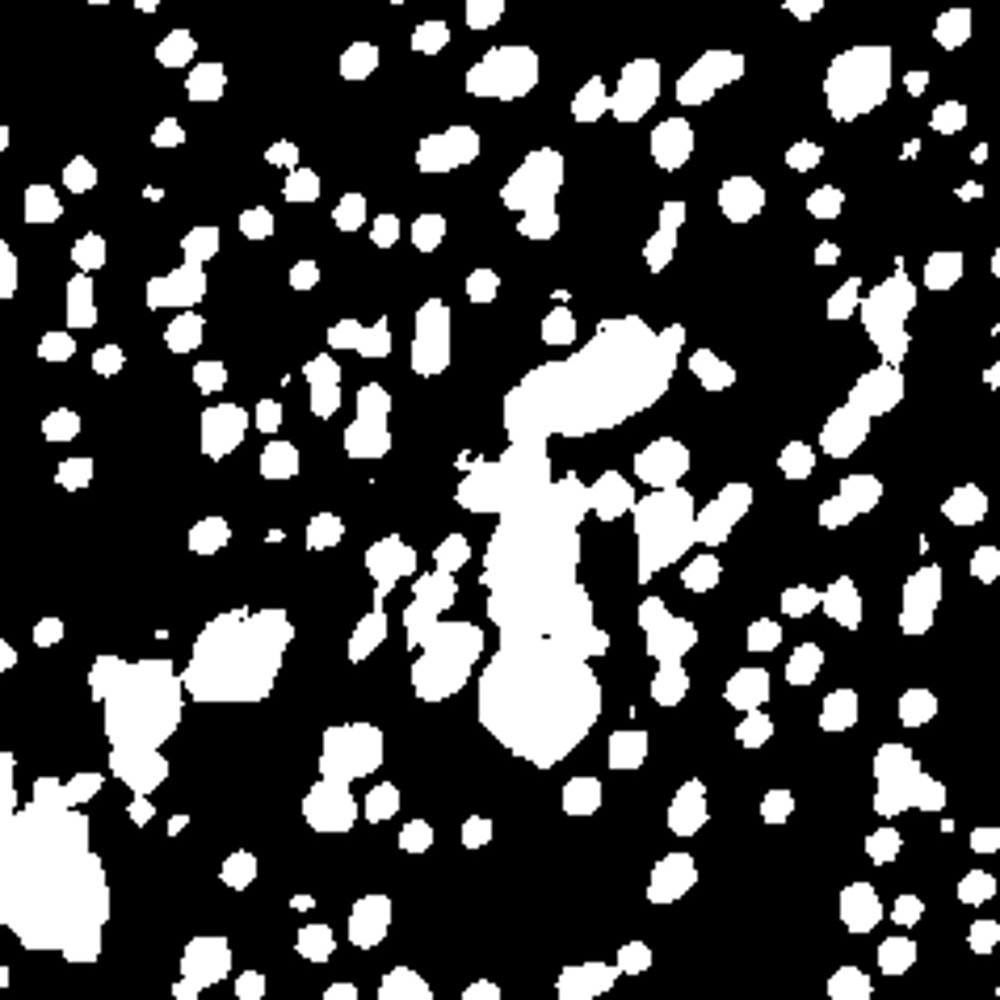

Supplement: Supplementary file 3 — Source Data [file 41467_2023_38178_MOESM3_ESM.zip › Source Data/Fig 3/48.jpg]

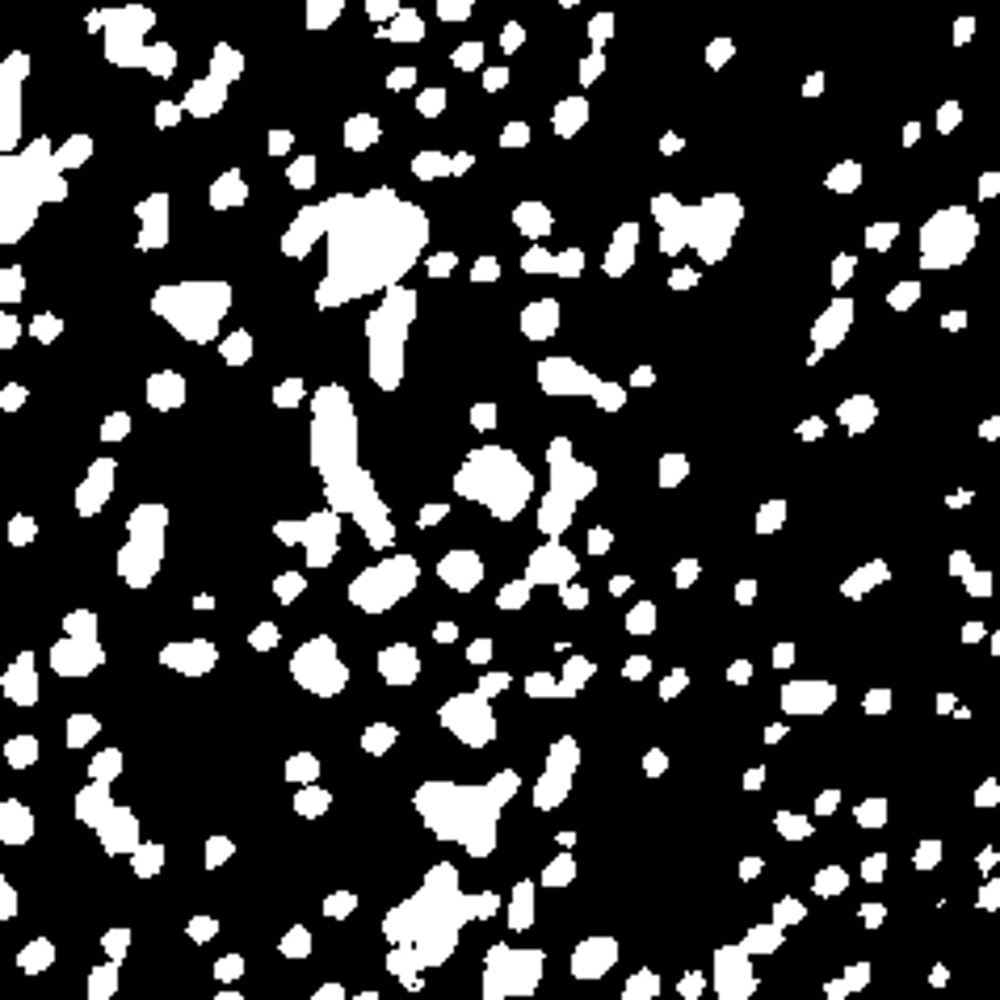

Supplement: Supplementary file 3 — Source Data [file 41467_2023_38178_MOESM3_ESM.zip › Source Data/Fig 3/49.jpg]

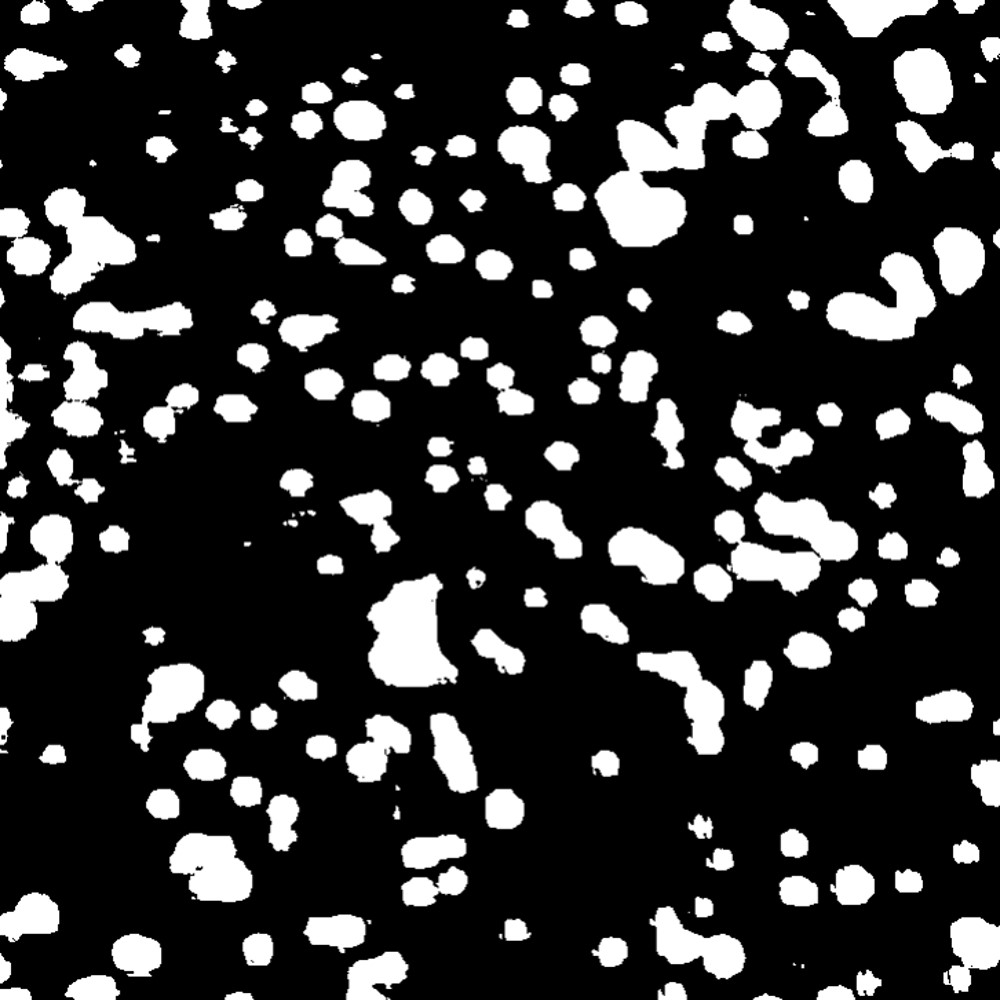

Supplement: Supplementary file 3 — Source Data [file 41467_2023_38178_MOESM3_ESM.zip › Source Data/Fig 3/5.jpg]

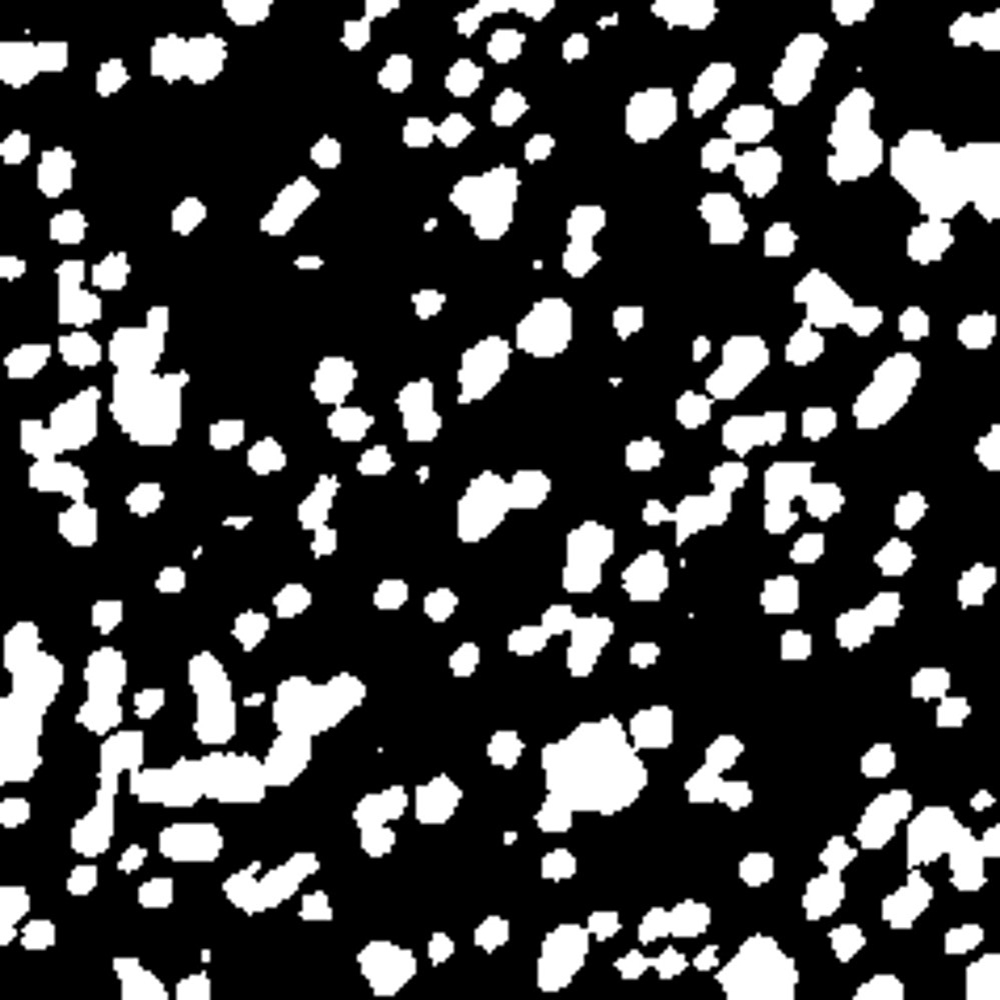

Supplement: Supplementary file 3 — Source Data [file 41467_2023_38178_MOESM3_ESM.zip › Source Data/Fig 3/50.jpg]

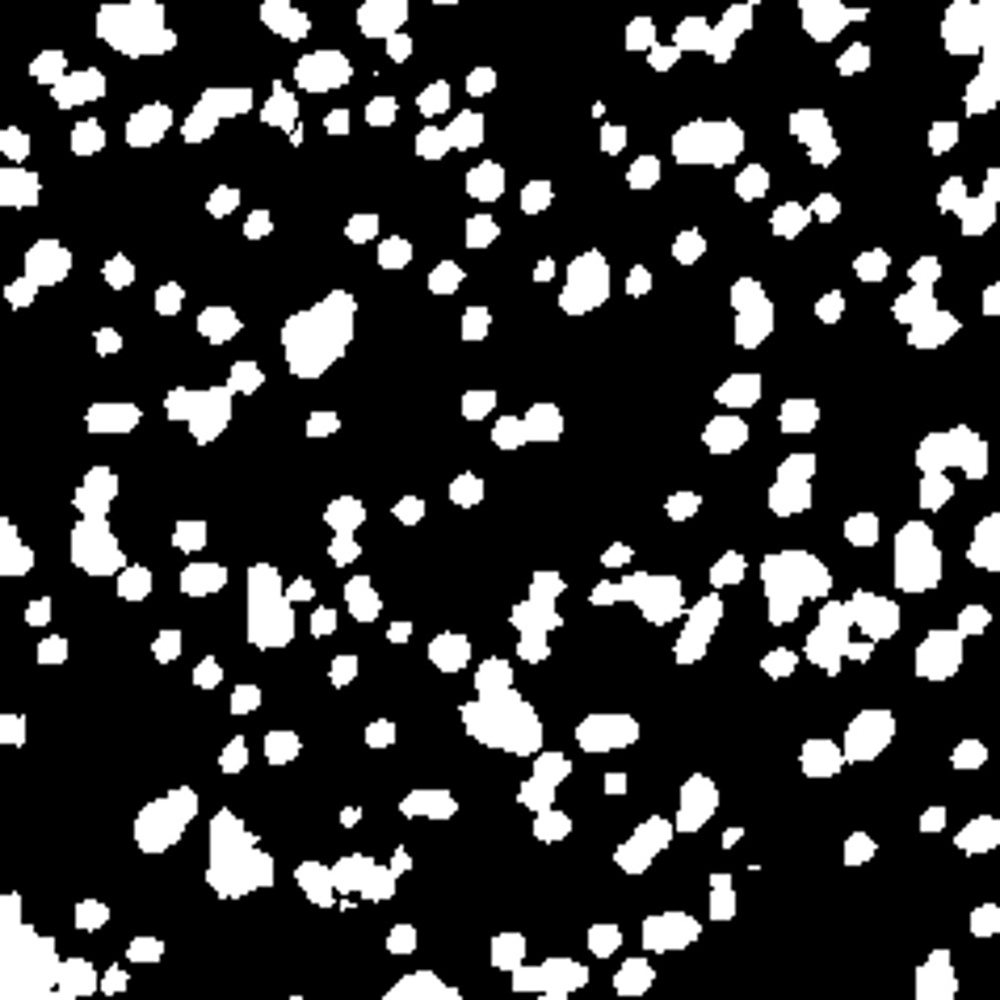

Supplement: Supplementary file 3 — Source Data [file 41467_2023_38178_MOESM3_ESM.zip › Source Data/Fig 3/51.jpg]

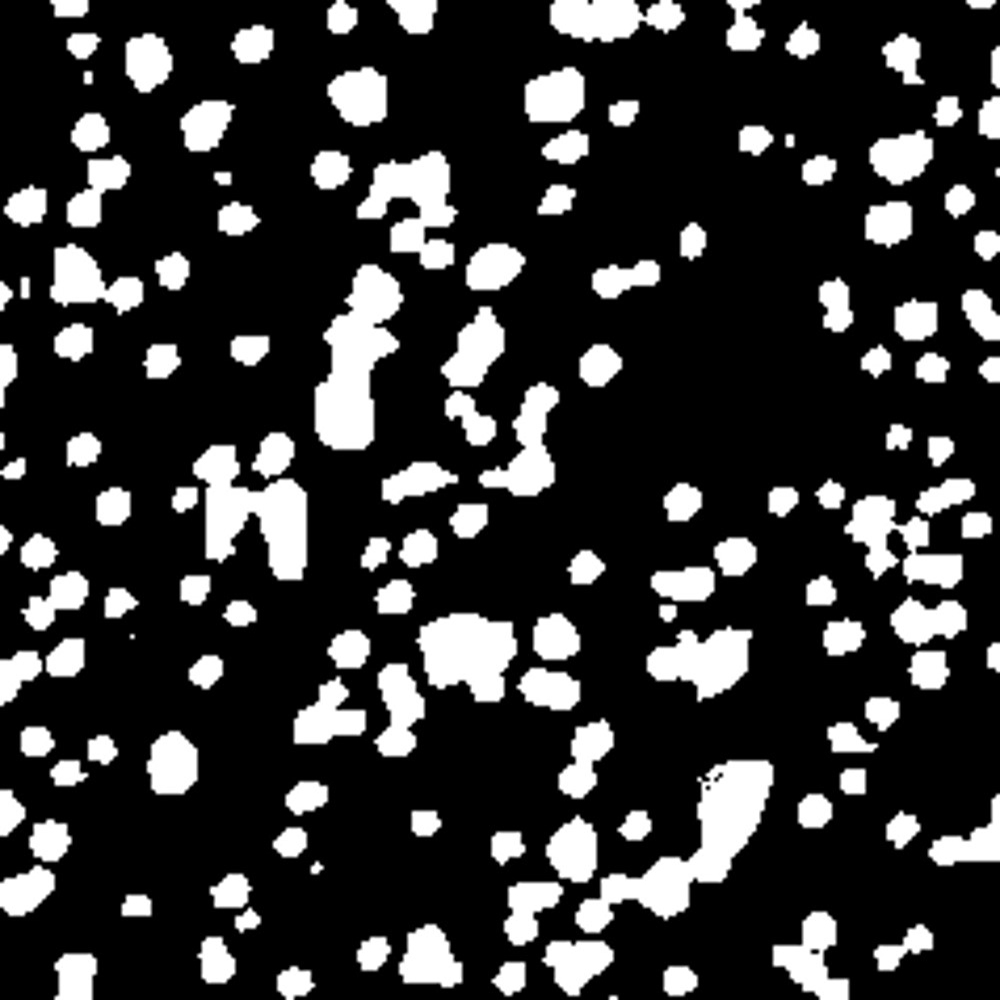

Supplement: Supplementary file 3 — Source Data [file 41467_2023_38178_MOESM3_ESM.zip › Source Data/Fig 3/52.jpg]

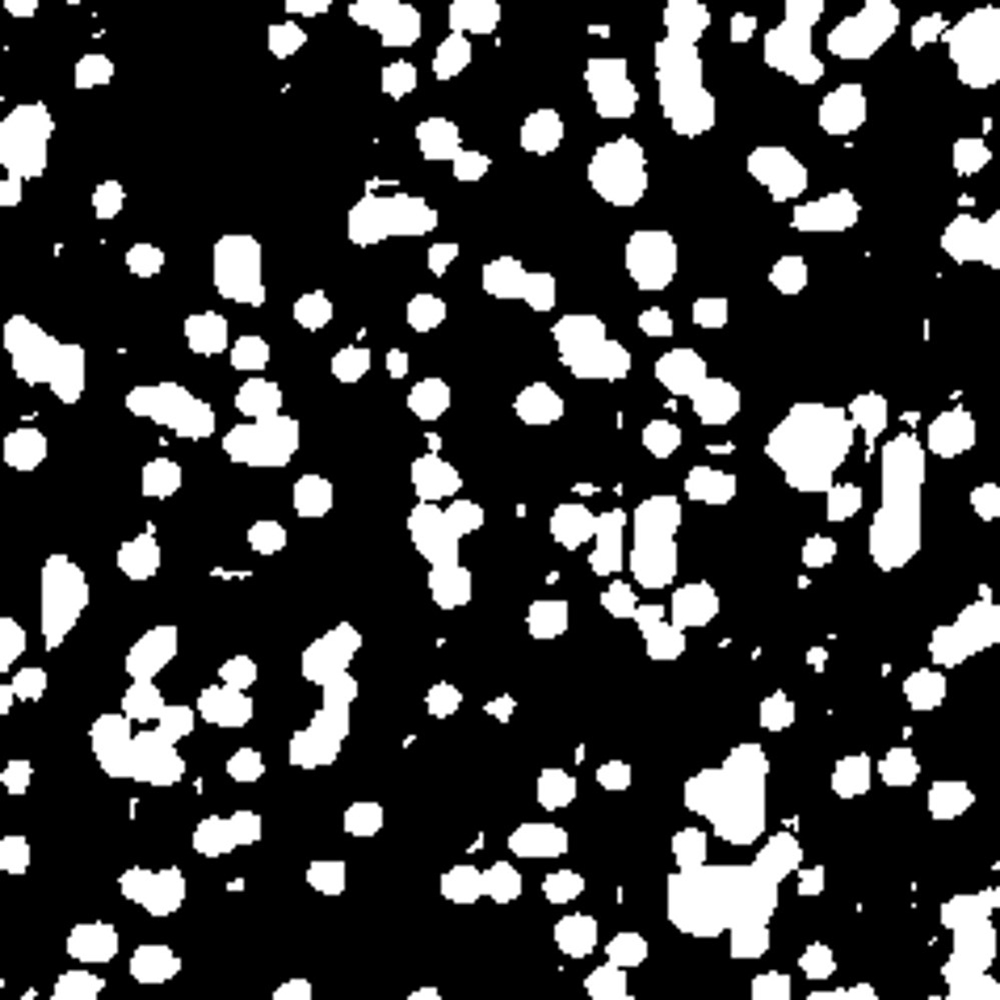

Supplement: Supplementary file 3 — Source Data [file 41467_2023_38178_MOESM3_ESM.zip › Source Data/Fig 3/53.jpg]

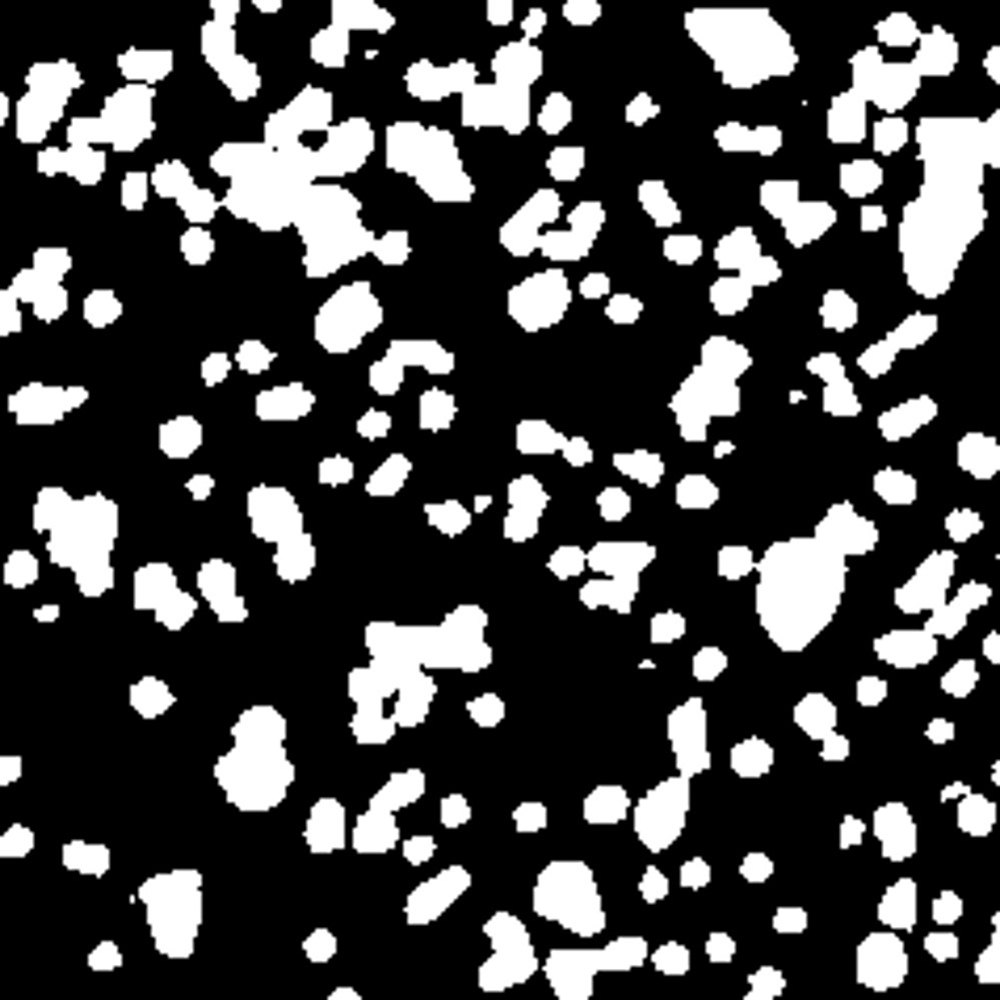

Supplement: Supplementary file 3 — Source Data [file 41467_2023_38178_MOESM3_ESM.zip › Source Data/Fig 3/54.jpg]

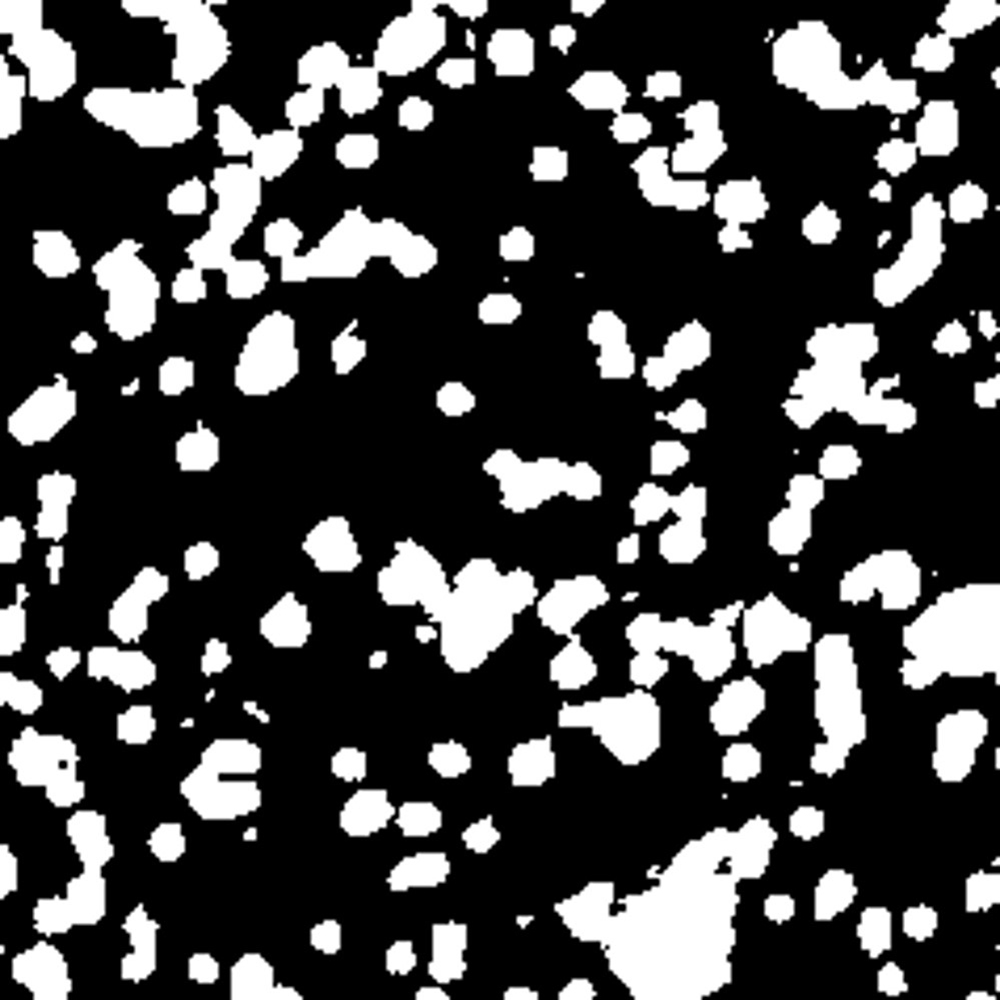

Supplement: Supplementary file 3 — Source Data [file 41467_2023_38178_MOESM3_ESM.zip › Source Data/Fig 3/55.jpg]

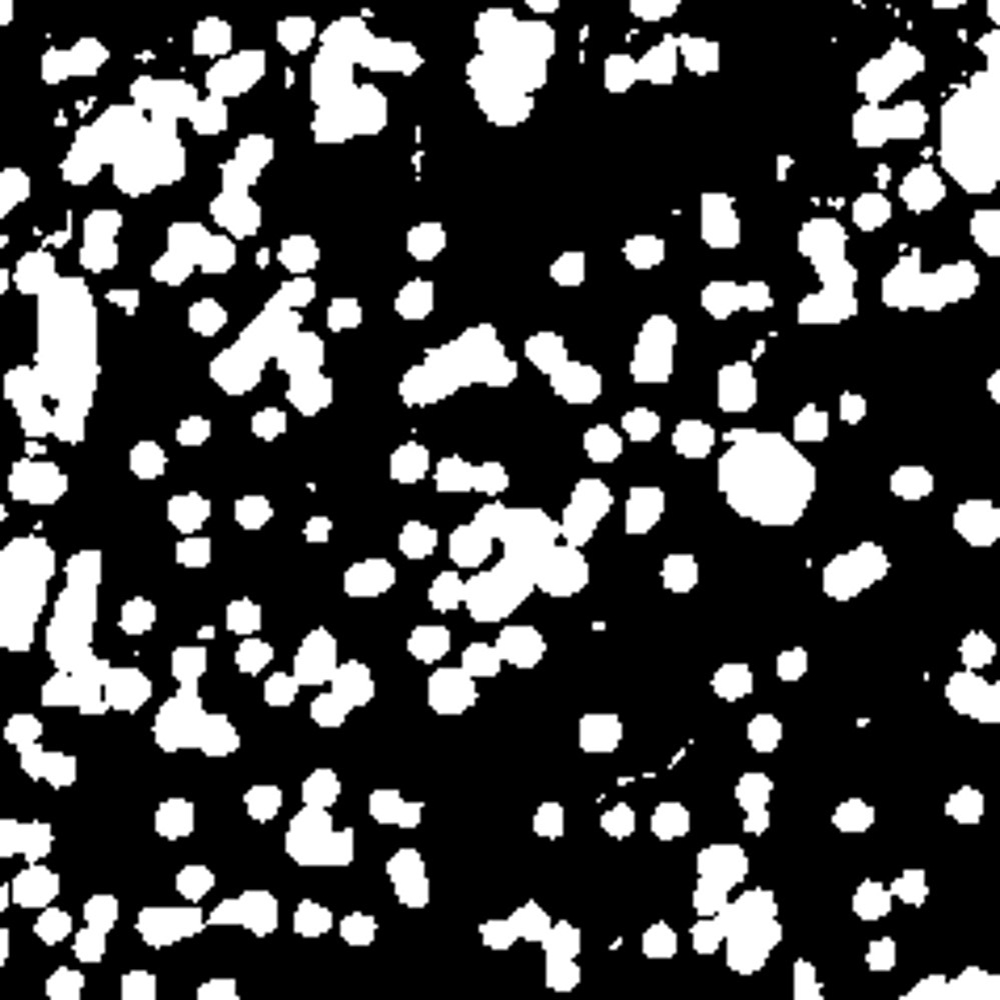

Supplement: Supplementary file 3 — Source Data [file 41467_2023_38178_MOESM3_ESM.zip › Source Data/Fig 3/56.jpg]

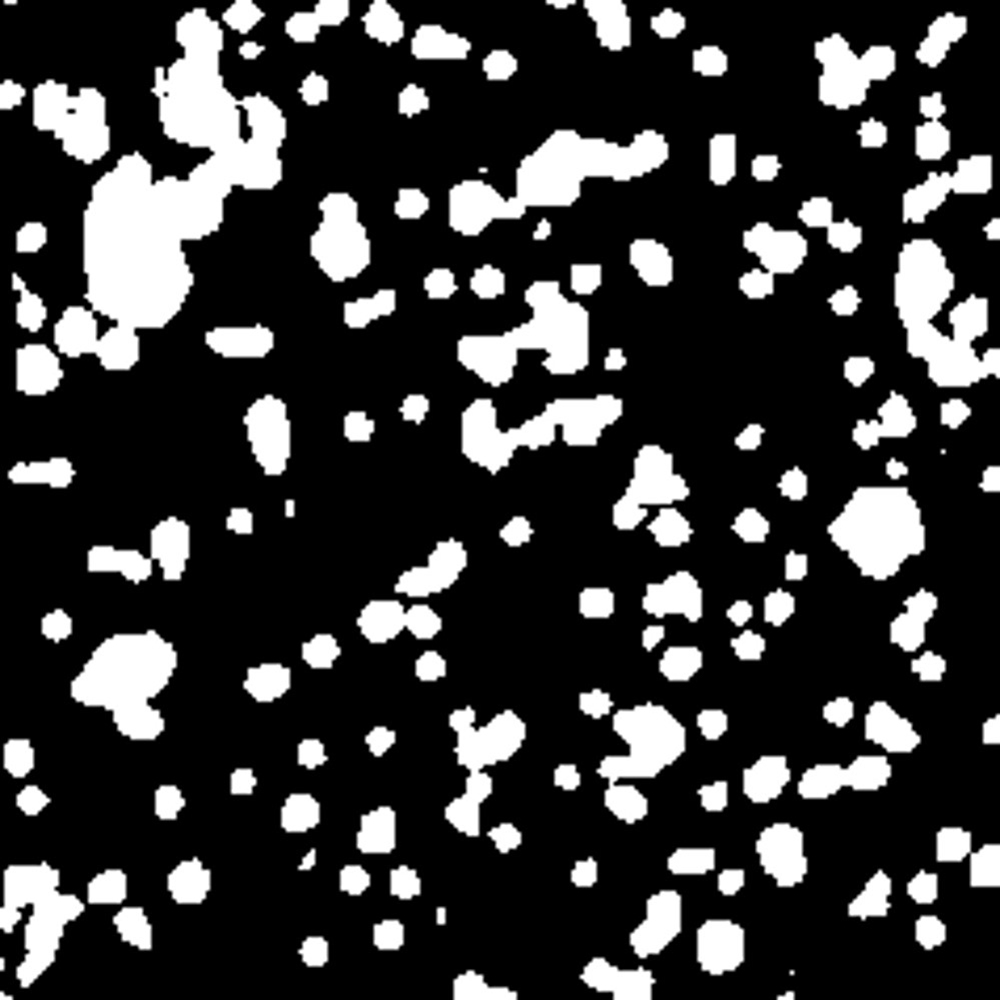

Supplement: Supplementary file 3 — Source Data [file 41467_2023_38178_MOESM3_ESM.zip › Source Data/Fig 3/57.jpg]

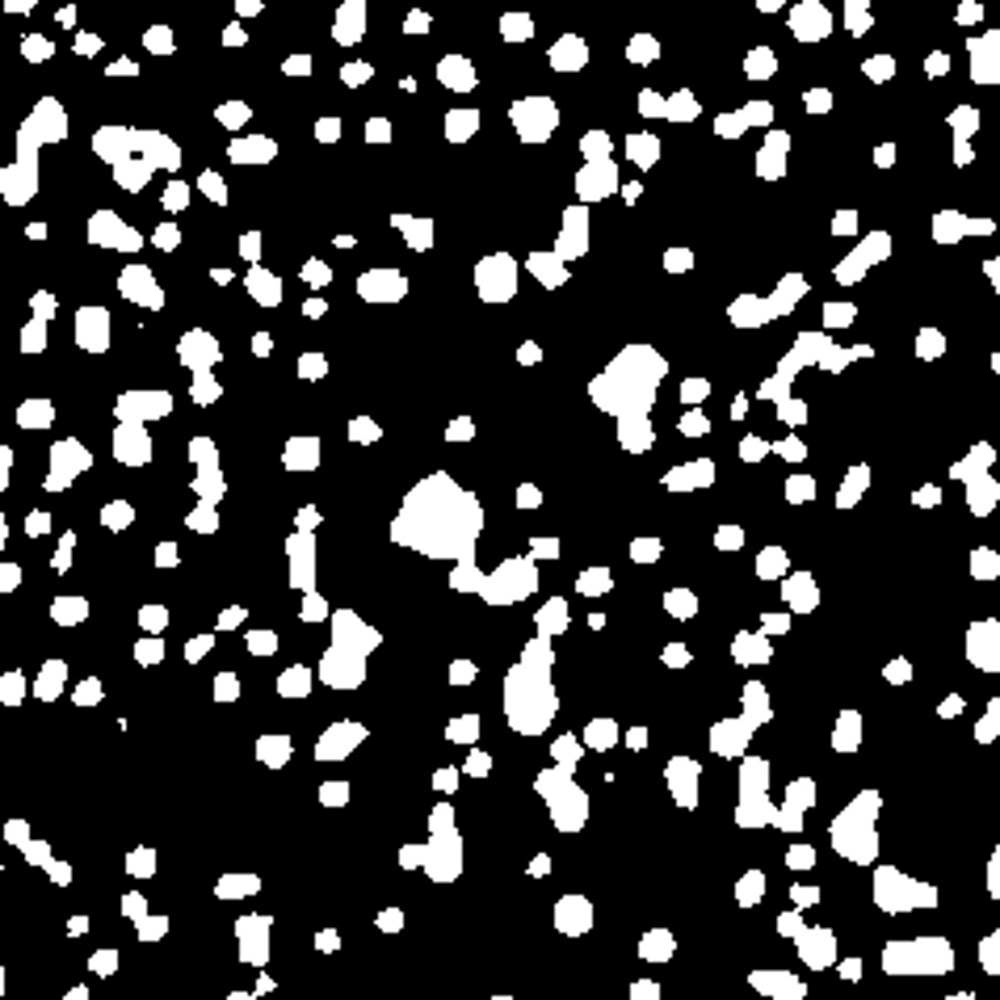

Supplement: Supplementary file 3 — Source Data [file 41467_2023_38178_MOESM3_ESM.zip › Source Data/Fig 3/58.jpg]

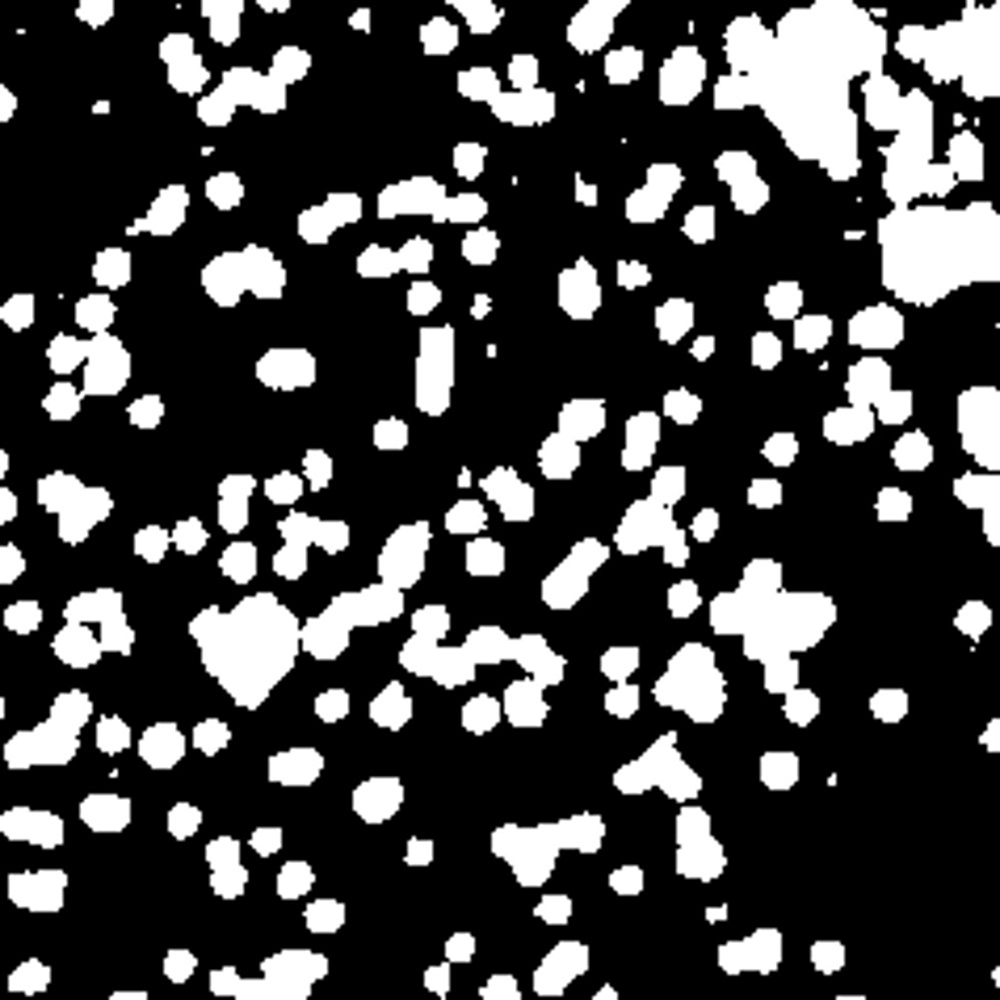

Supplement: Supplementary file 3 — Source Data [file 41467_2023_38178_MOESM3_ESM.zip › Source Data/Fig 3/59.jpg]

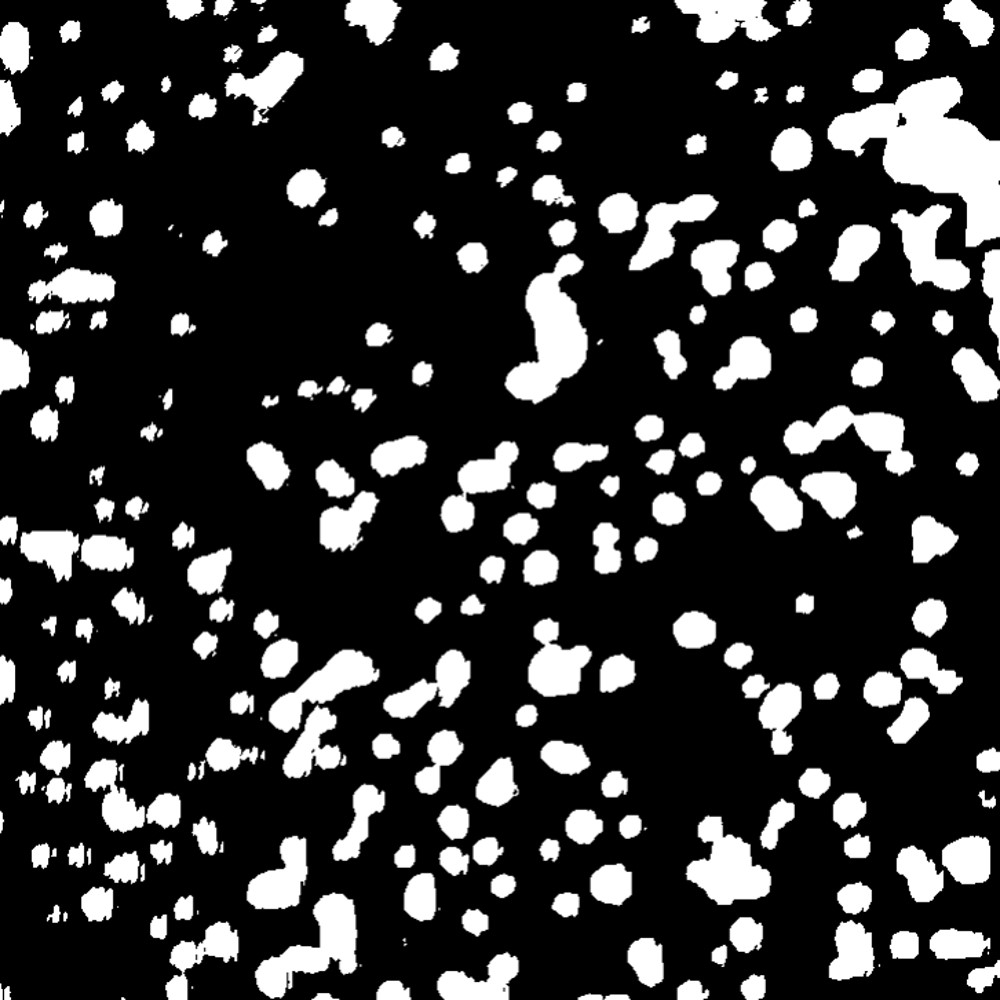

Supplement: Supplementary file 3 — Source Data [file 41467_2023_38178_MOESM3_ESM.zip › Source Data/Fig 3/6.jpg]

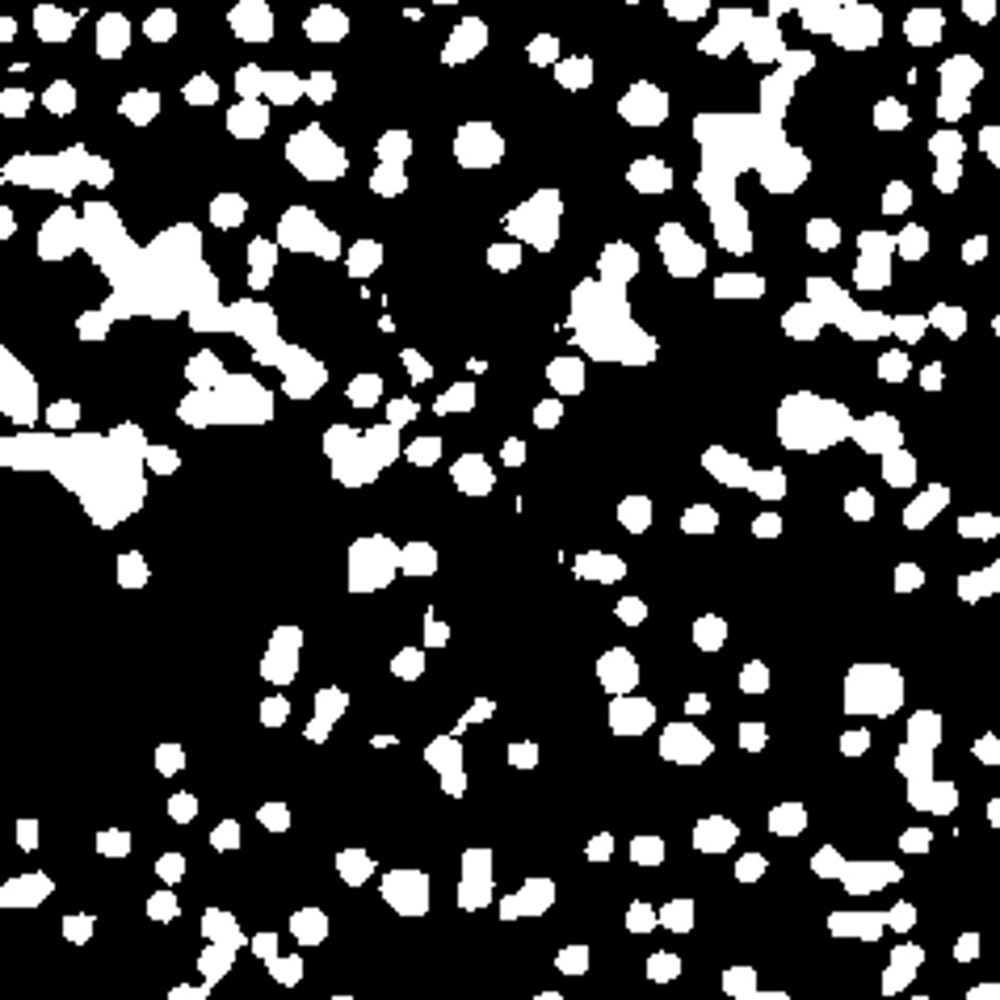

Supplement: Supplementary file 3 — Source Data [file 41467_2023_38178_MOESM3_ESM.zip › Source Data/Fig 3/60.jpg]

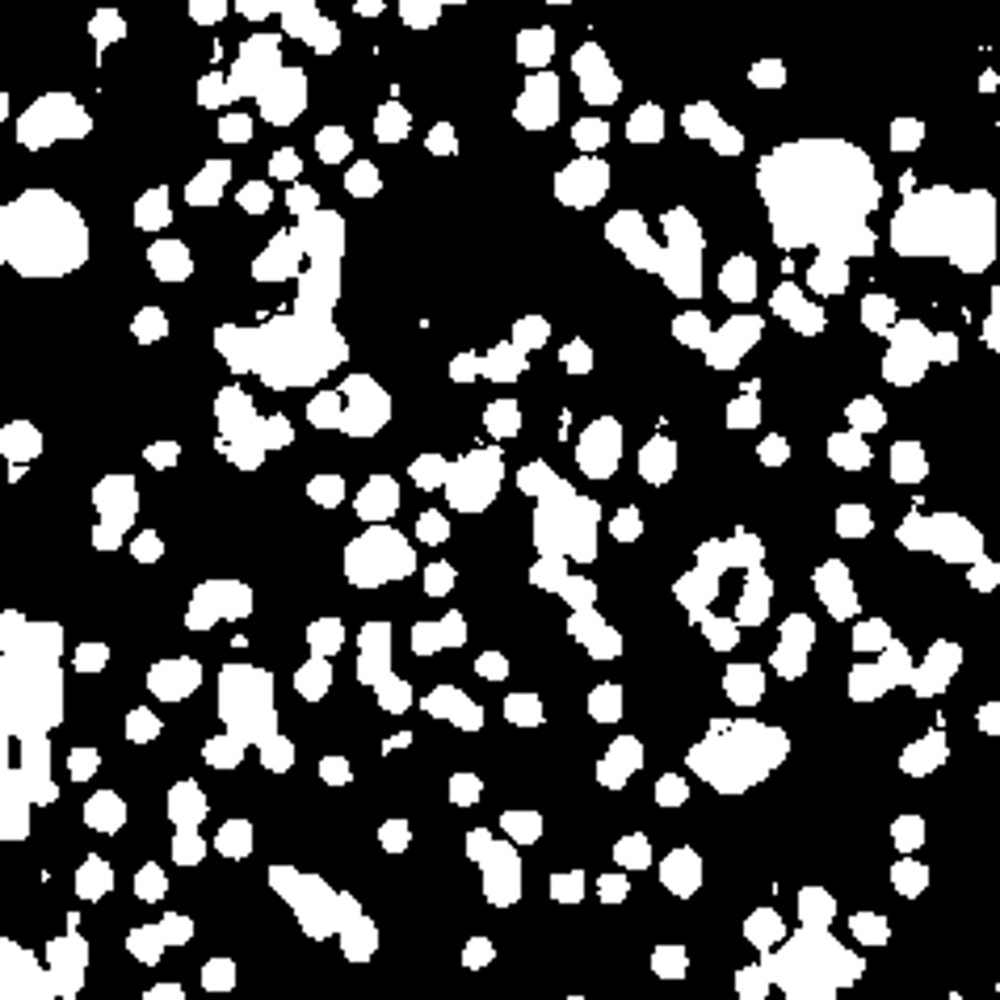

Supplement: Supplementary file 3 — Source Data [file 41467_2023_38178_MOESM3_ESM.zip › Source Data/Fig 3/61.jpg]

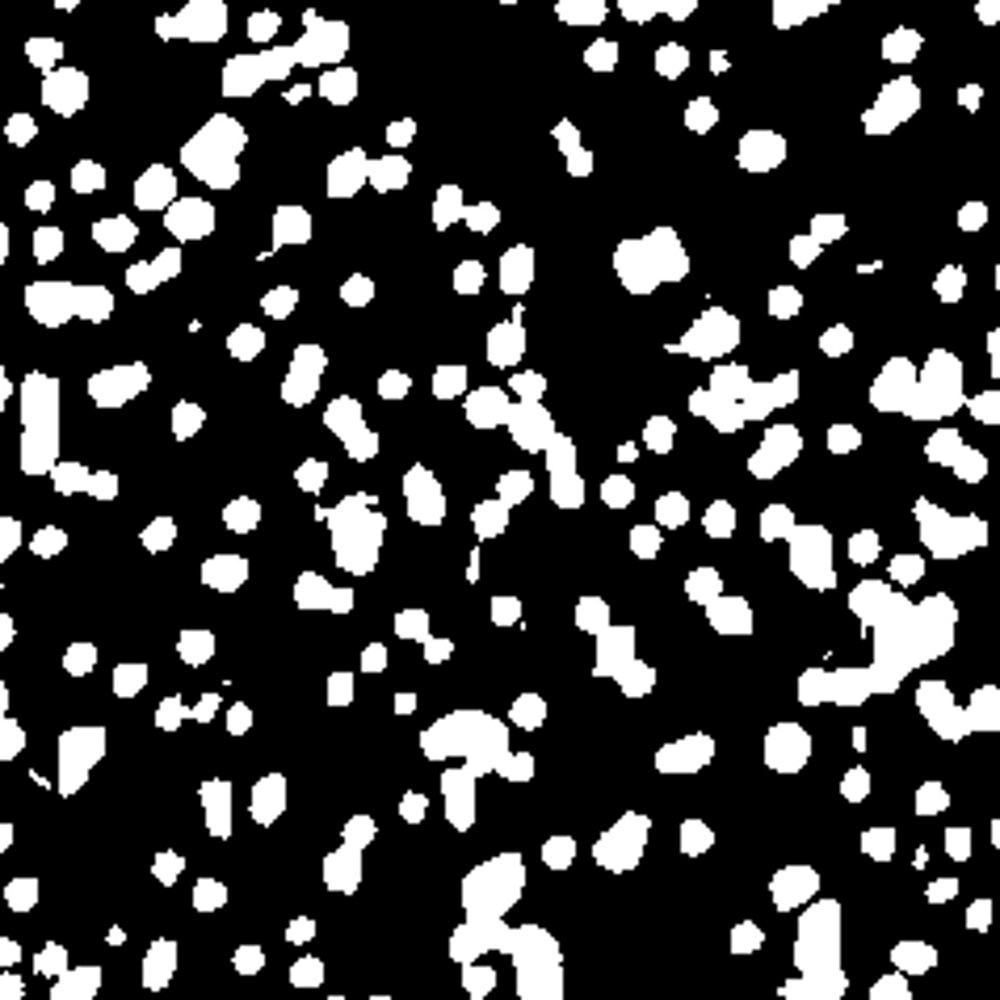

Supplement: Supplementary file 3 — Source Data [file 41467_2023_38178_MOESM3_ESM.zip › Source Data/Fig 3/62.jpg]

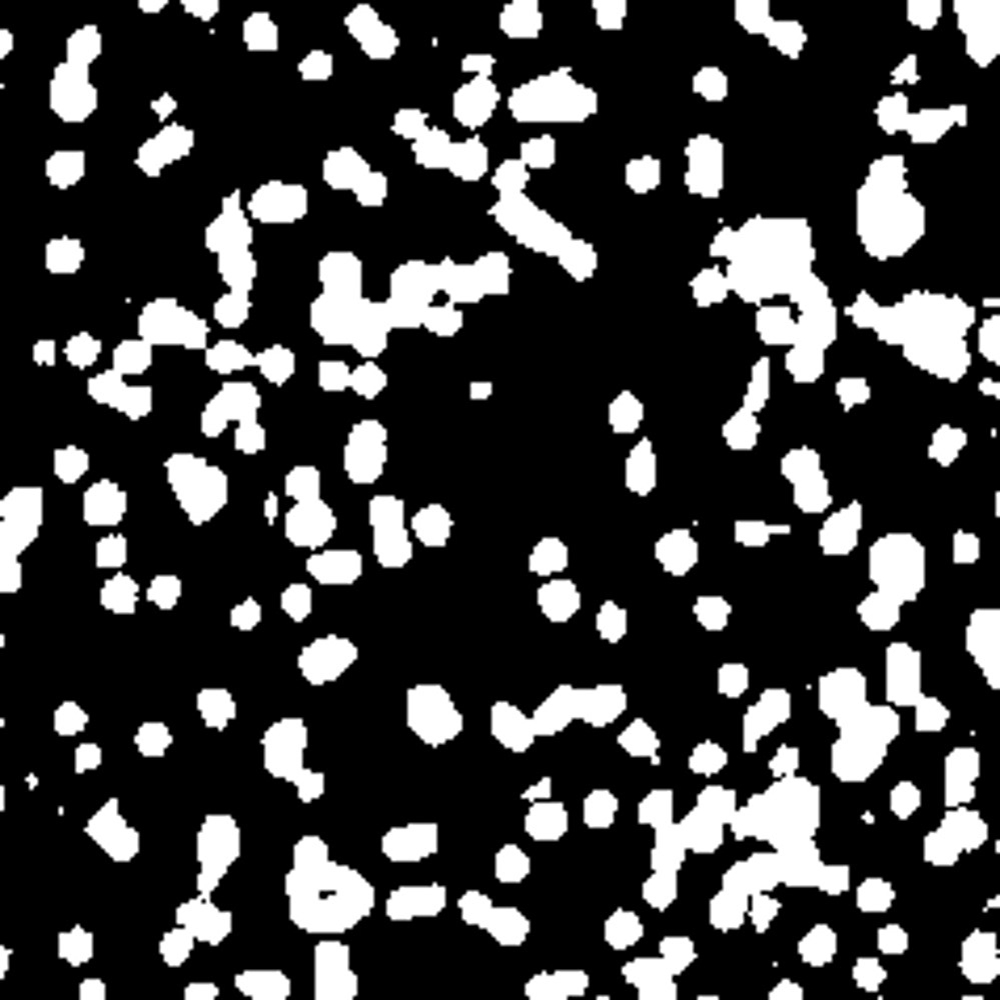

Supplement: Supplementary file 3 — Source Data [file 41467_2023_38178_MOESM3_ESM.zip › Source Data/Fig 3/63.jpg]

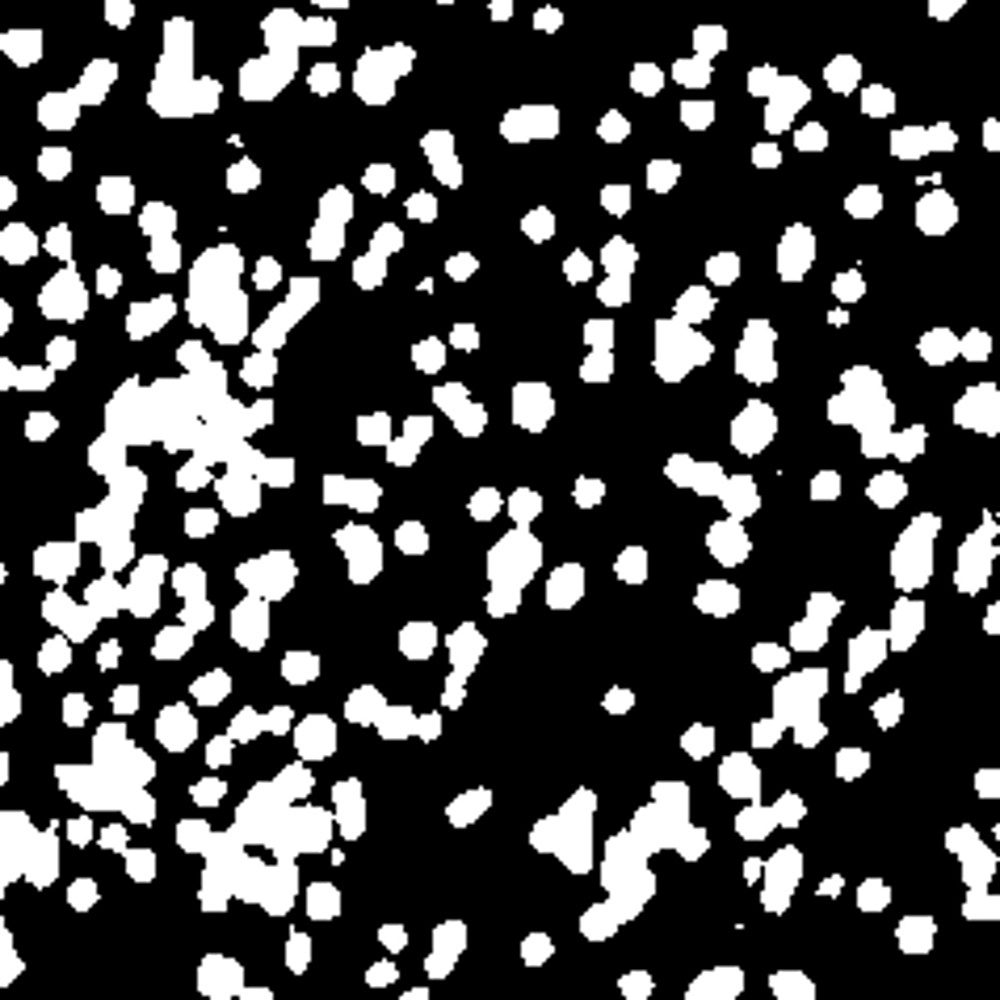

Supplement: Supplementary file 3 — Source Data [file 41467_2023_38178_MOESM3_ESM.zip › Source Data/Fig 3/64.jpg]

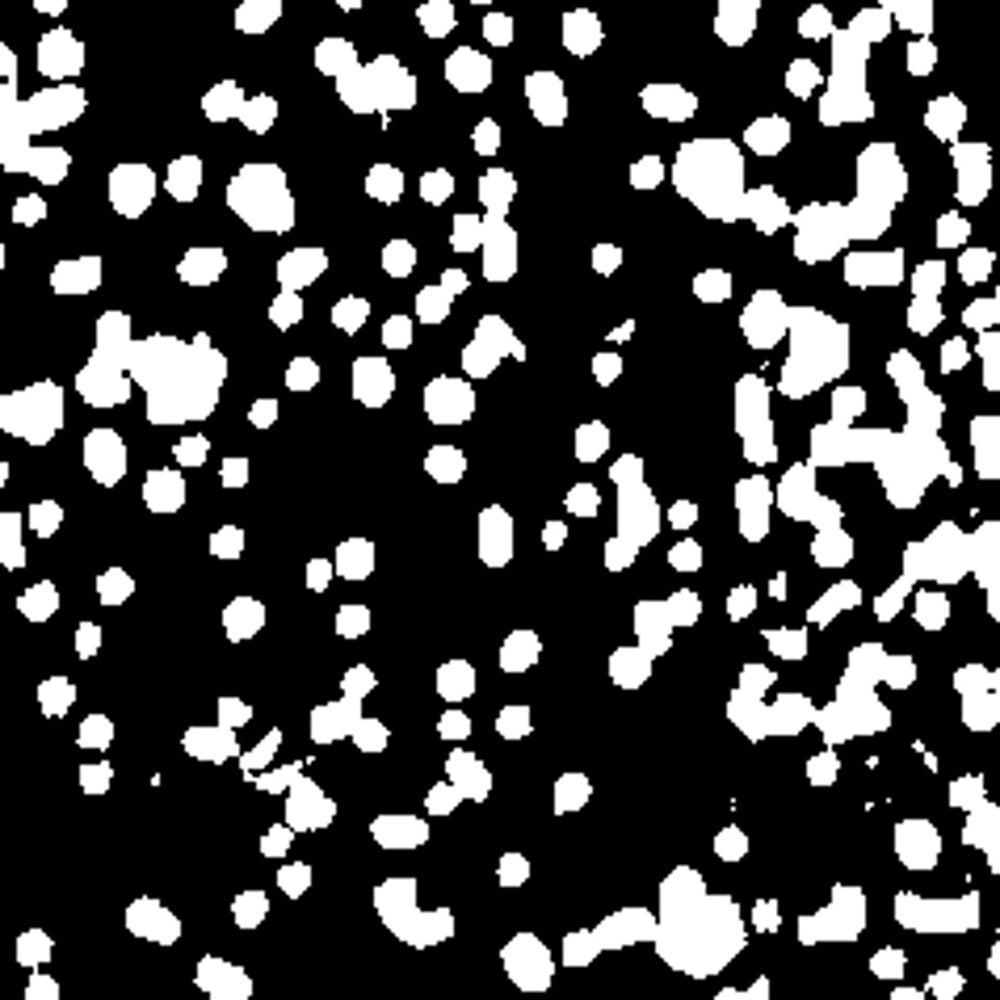

Supplement: Supplementary file 3 — Source Data [file 41467_2023_38178_MOESM3_ESM.zip › Source Data/Fig 3/65.jpg]

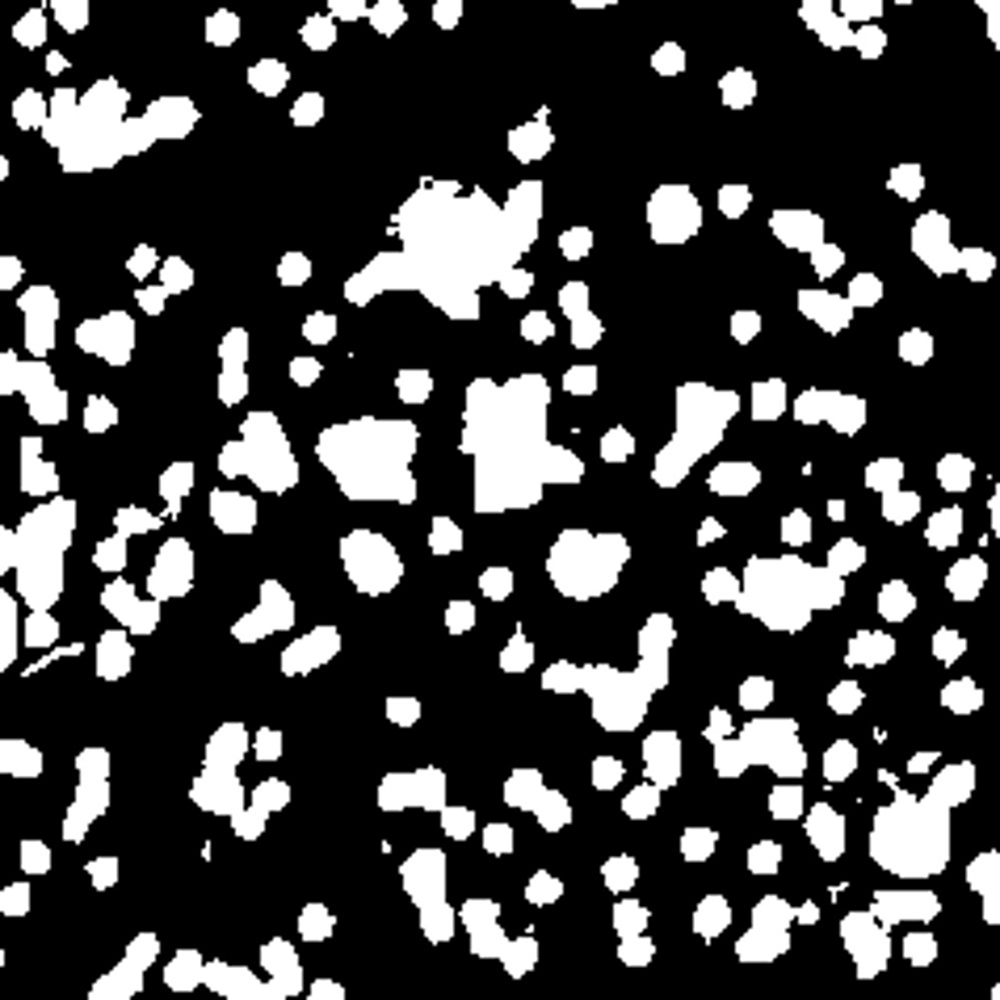

Supplement: Supplementary file 3 — Source Data [file 41467_2023_38178_MOESM3_ESM.zip › Source Data/Fig 3/66.jpg]

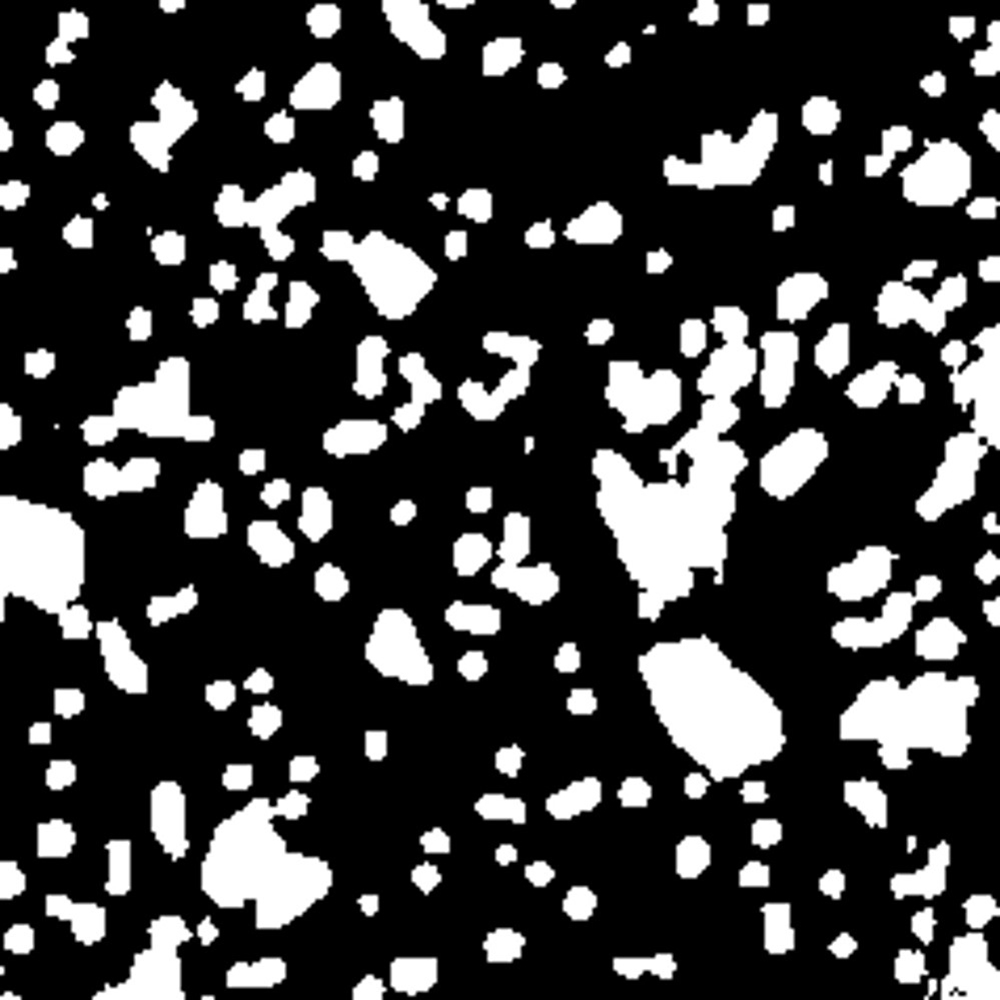

Supplement: Supplementary file 3 — Source Data [file 41467_2023_38178_MOESM3_ESM.zip › Source Data/Fig 3/67.jpg]

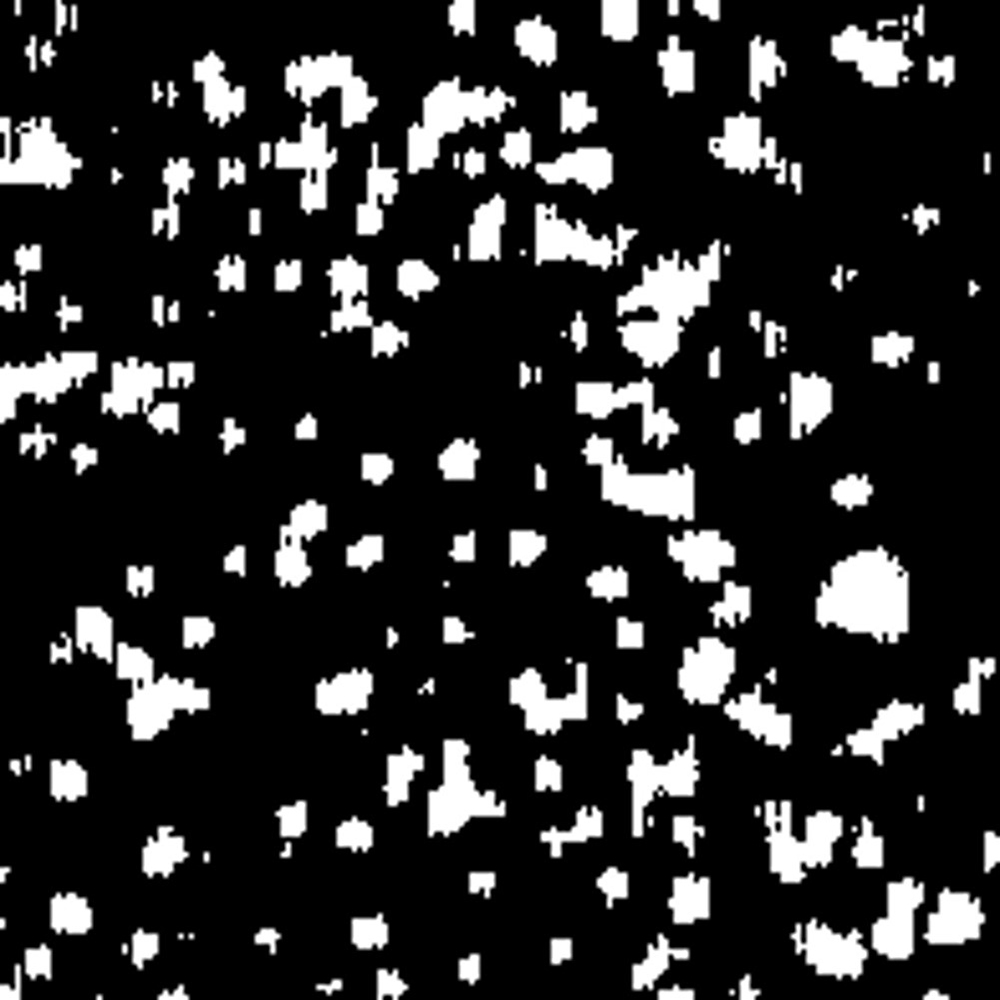

Supplement: Supplementary file 3 — Source Data [file 41467_2023_38178_MOESM3_ESM.zip › Source Data/Fig 3/68.jpg]

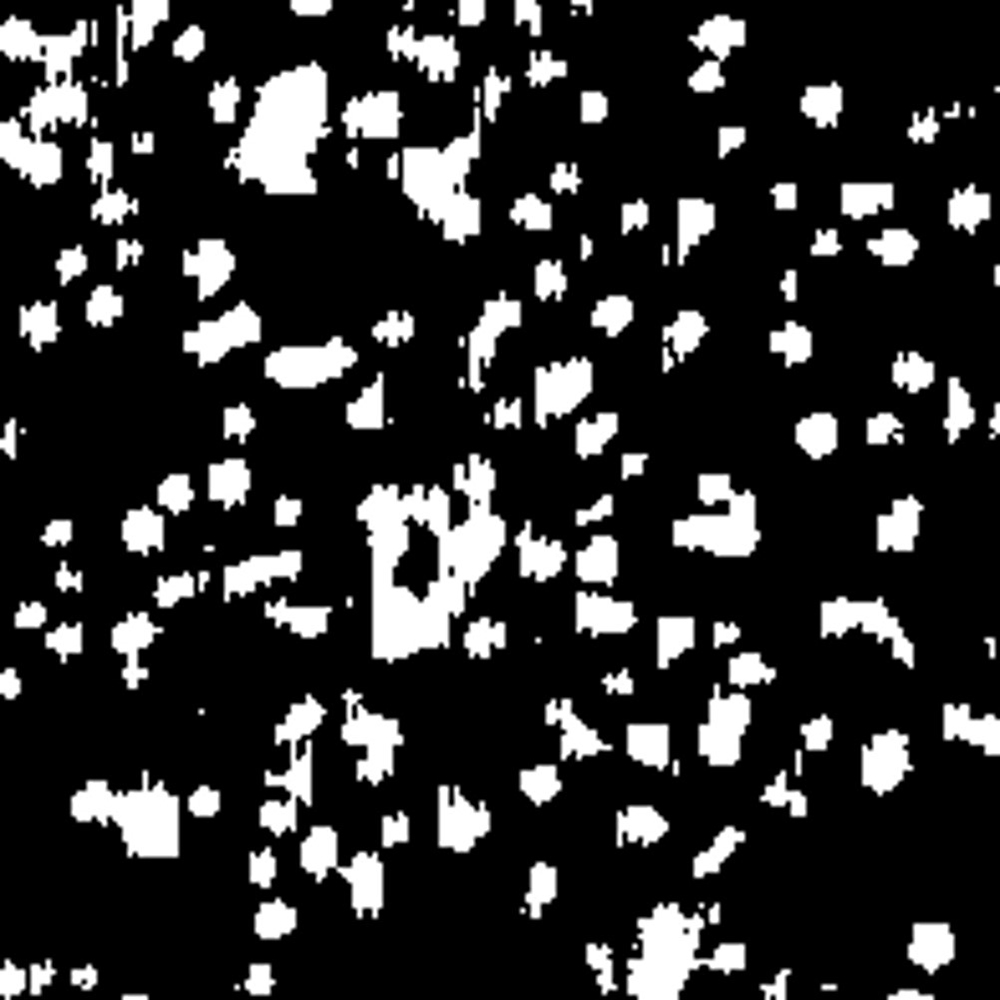

Supplement: Supplementary file 3 — Source Data [file 41467_2023_38178_MOESM3_ESM.zip › Source Data/Fig 3/69.jpg]

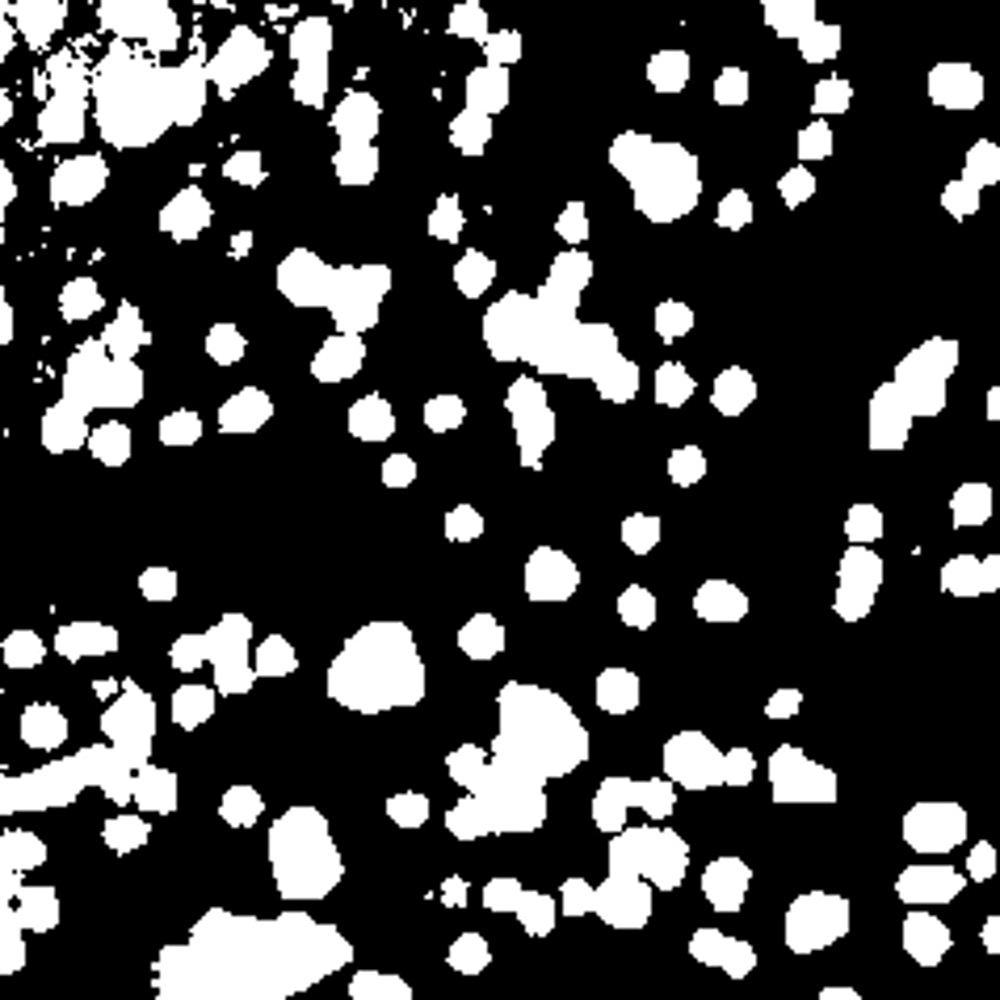

Supplement: Supplementary file 3 — Source Data [file 41467_2023_38178_MOESM3_ESM.zip › Source Data/Fig 3/7.jpg]

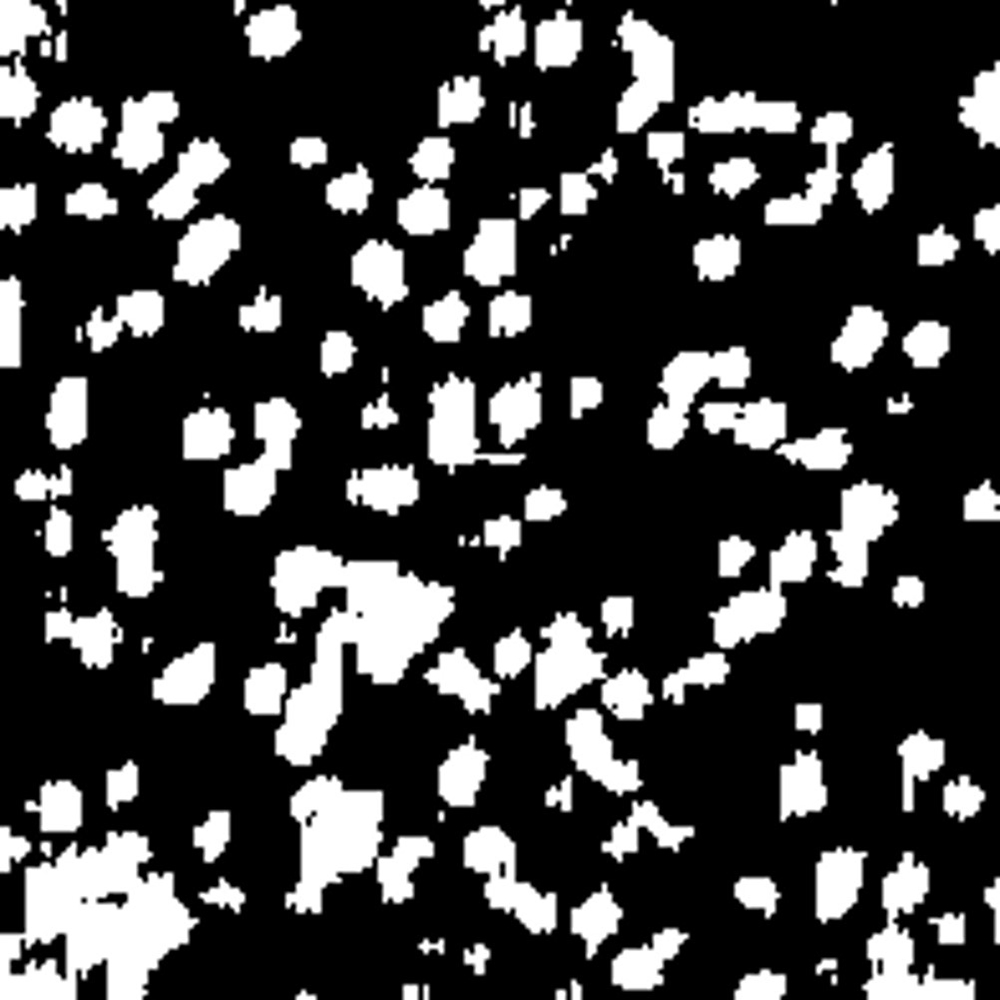

Supplement: Supplementary file 3 — Source Data [file 41467_2023_38178_MOESM3_ESM.zip › Source Data/Fig 3/70.jpg]

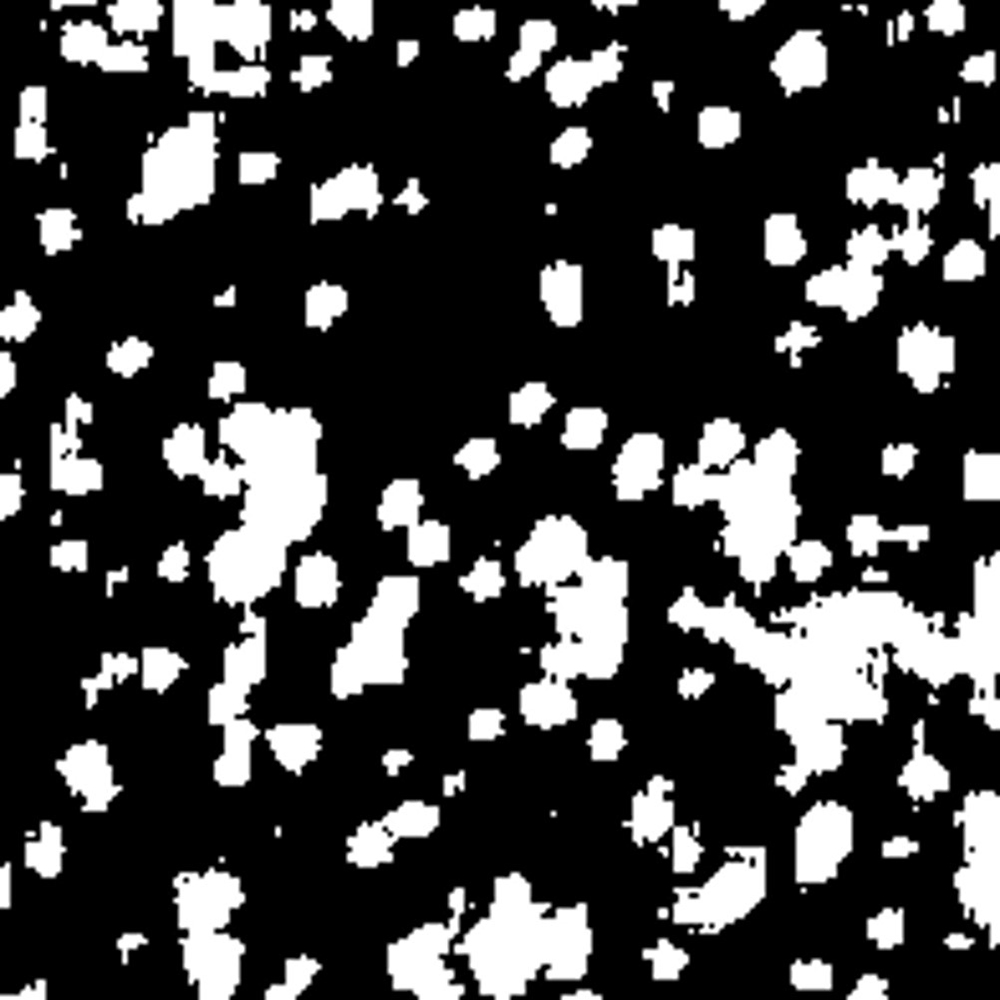

Supplement: Supplementary file 3 — Source Data [file 41467_2023_38178_MOESM3_ESM.zip › Source Data/Fig 3/71.jpg]

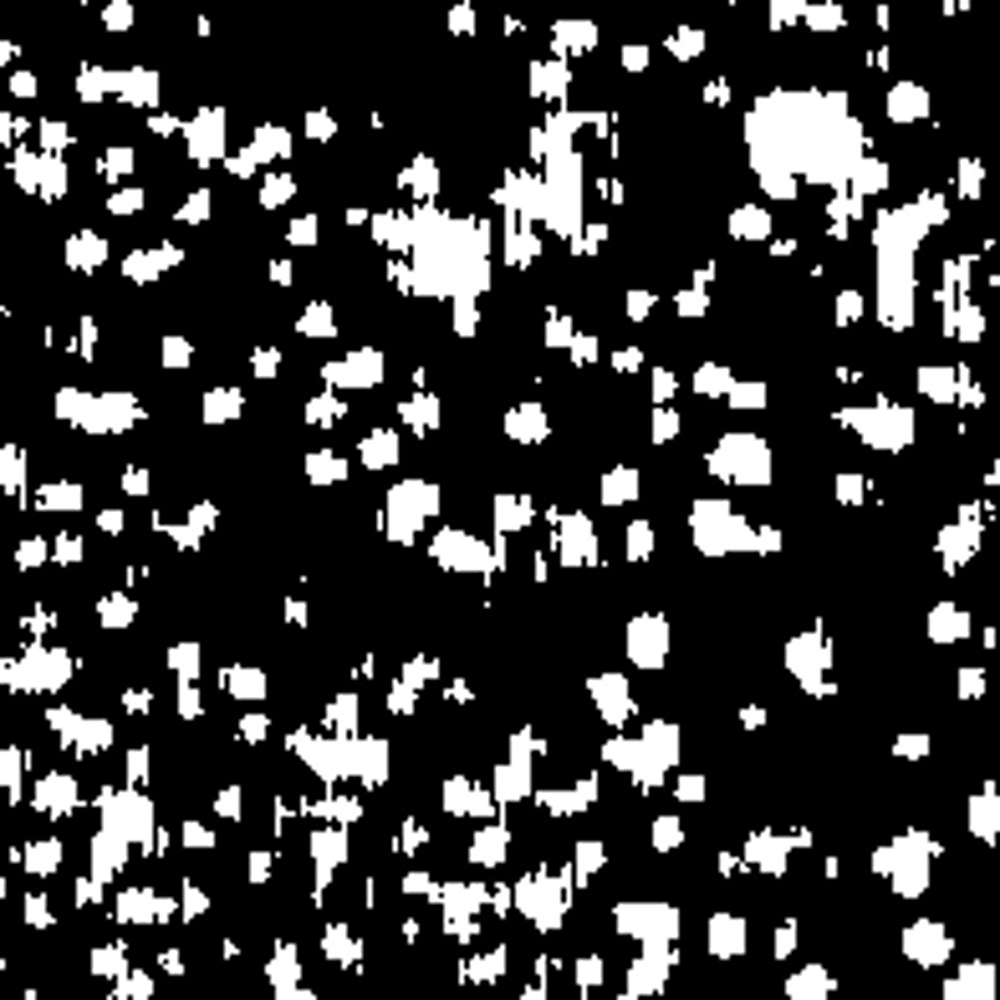

Supplement: Supplementary file 3 — Source Data [file 41467_2023_38178_MOESM3_ESM.zip › Source Data/Fig 3/72.jpg]

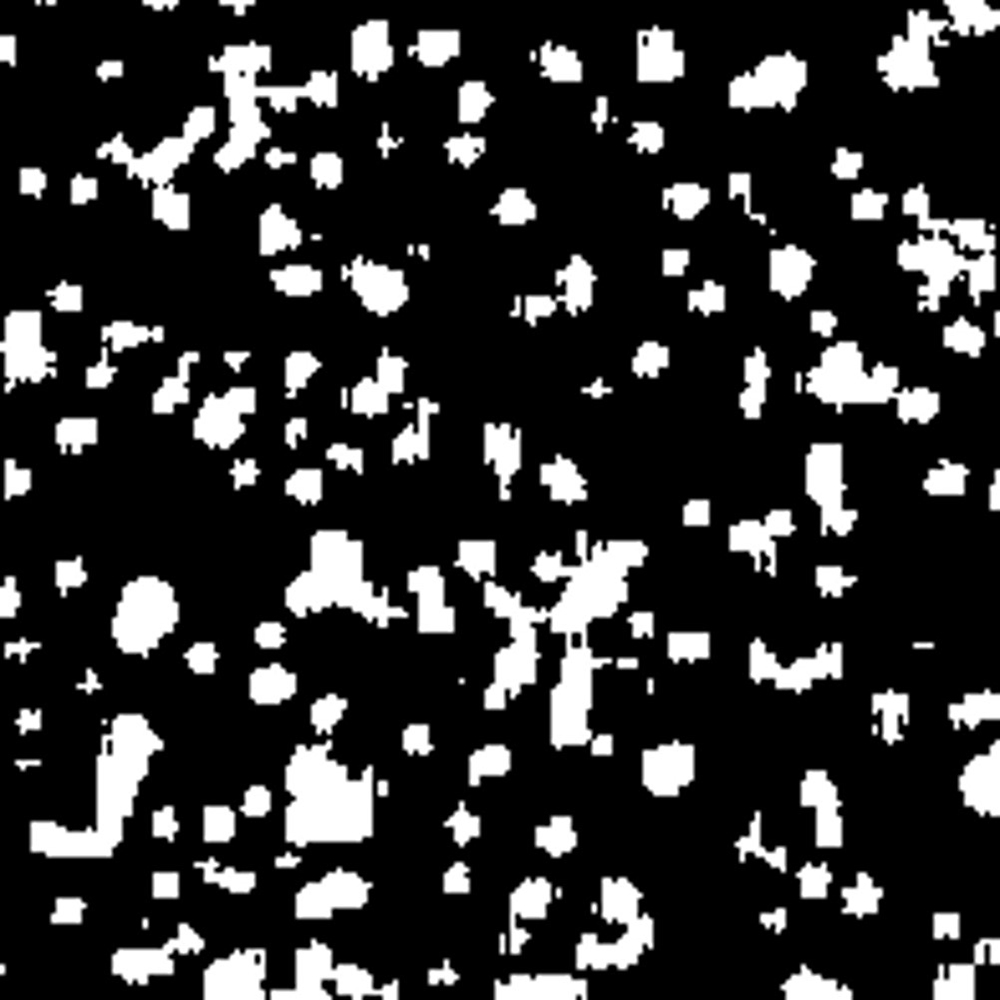

Supplement: Supplementary file 3 — Source Data [file 41467_2023_38178_MOESM3_ESM.zip › Source Data/Fig 3/73.jpg]

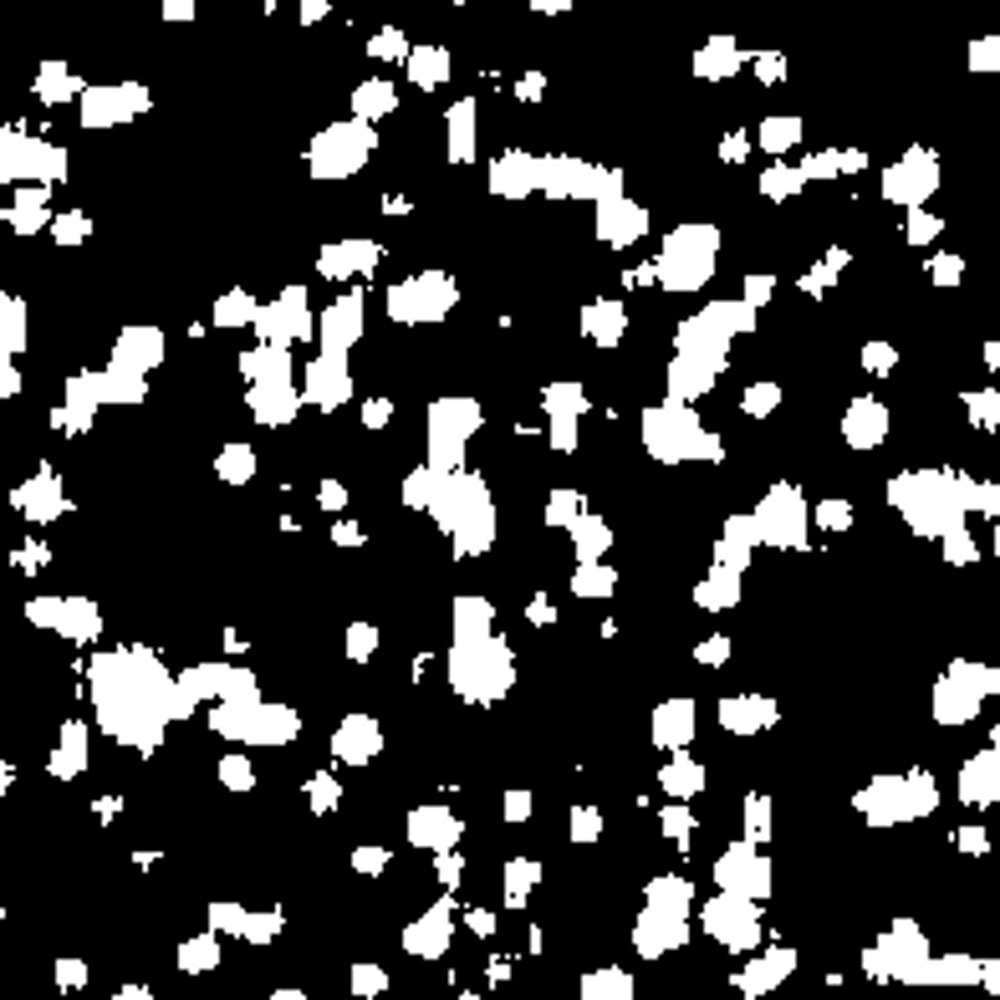

Supplement: Supplementary file 3 — Source Data [file 41467_2023_38178_MOESM3_ESM.zip › Source Data/Fig 3/74.jpg]

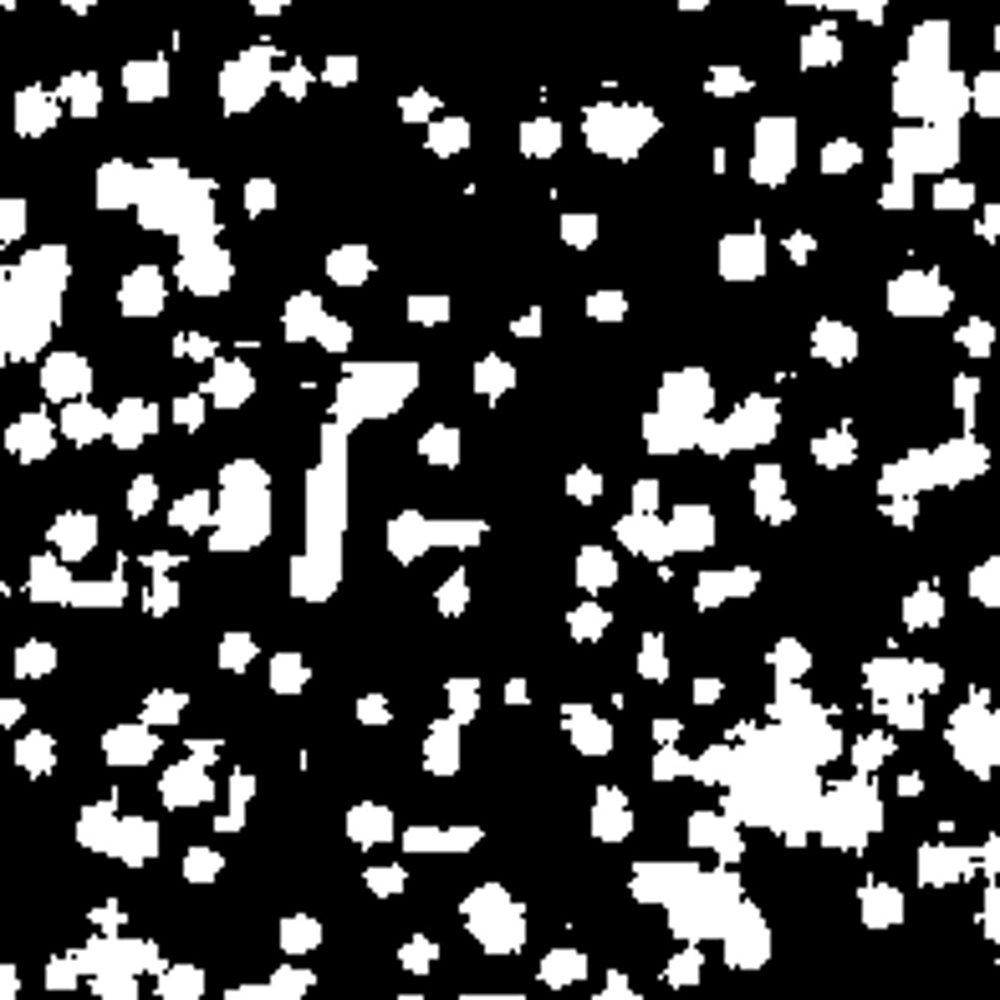

Supplement: Supplementary file 3 — Source Data [file 41467_2023_38178_MOESM3_ESM.zip › Source Data/Fig 3/75.jpg]

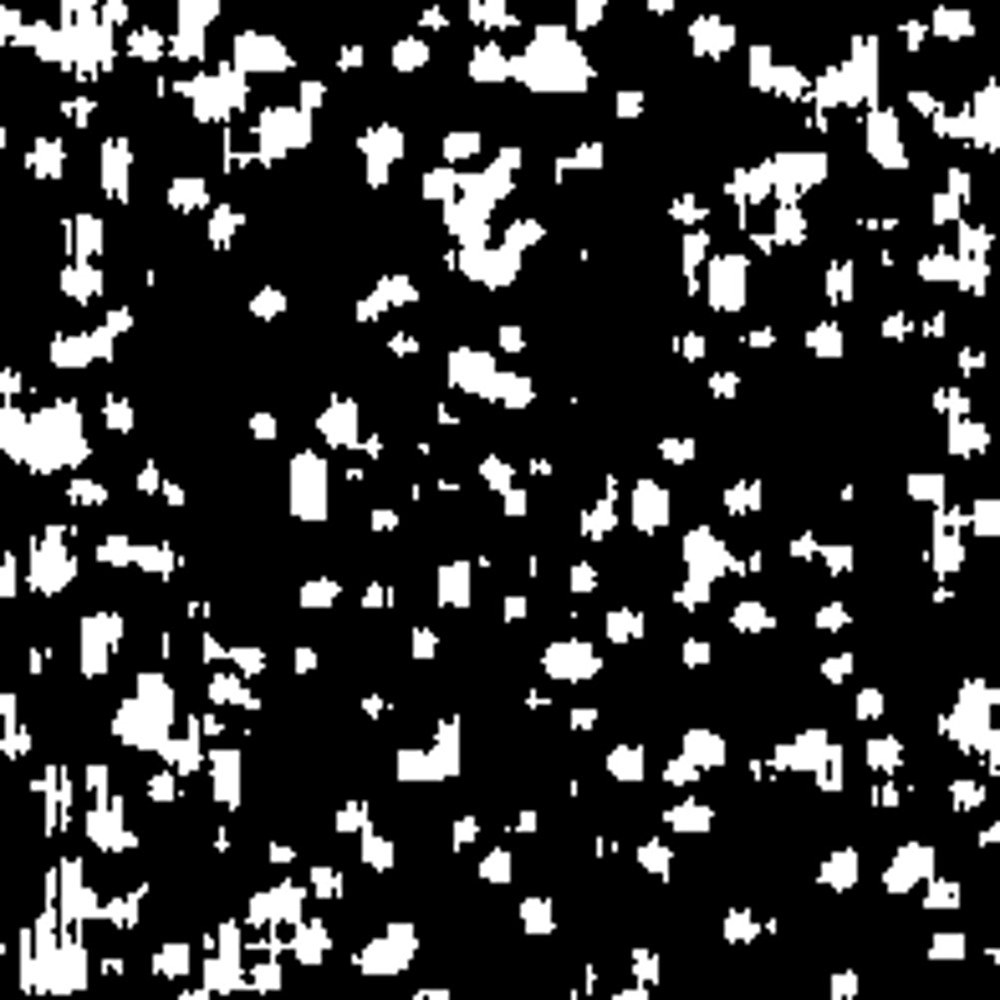

Supplement: Supplementary file 3 — Source Data [file 41467_2023_38178_MOESM3_ESM.zip › Source Data/Fig 3/76.jpg]

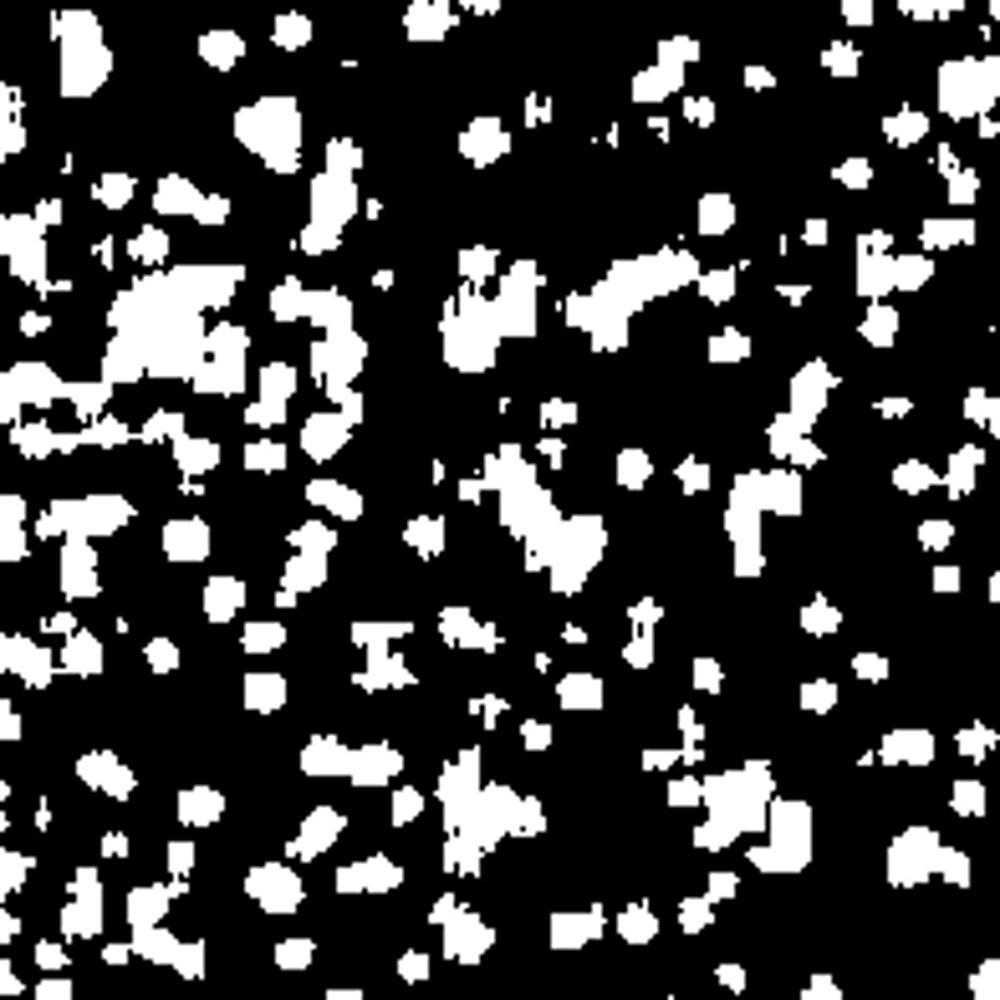

Supplement: Supplementary file 3 — Source Data [file 41467_2023_38178_MOESM3_ESM.zip › Source Data/Fig 3/77.jpg]

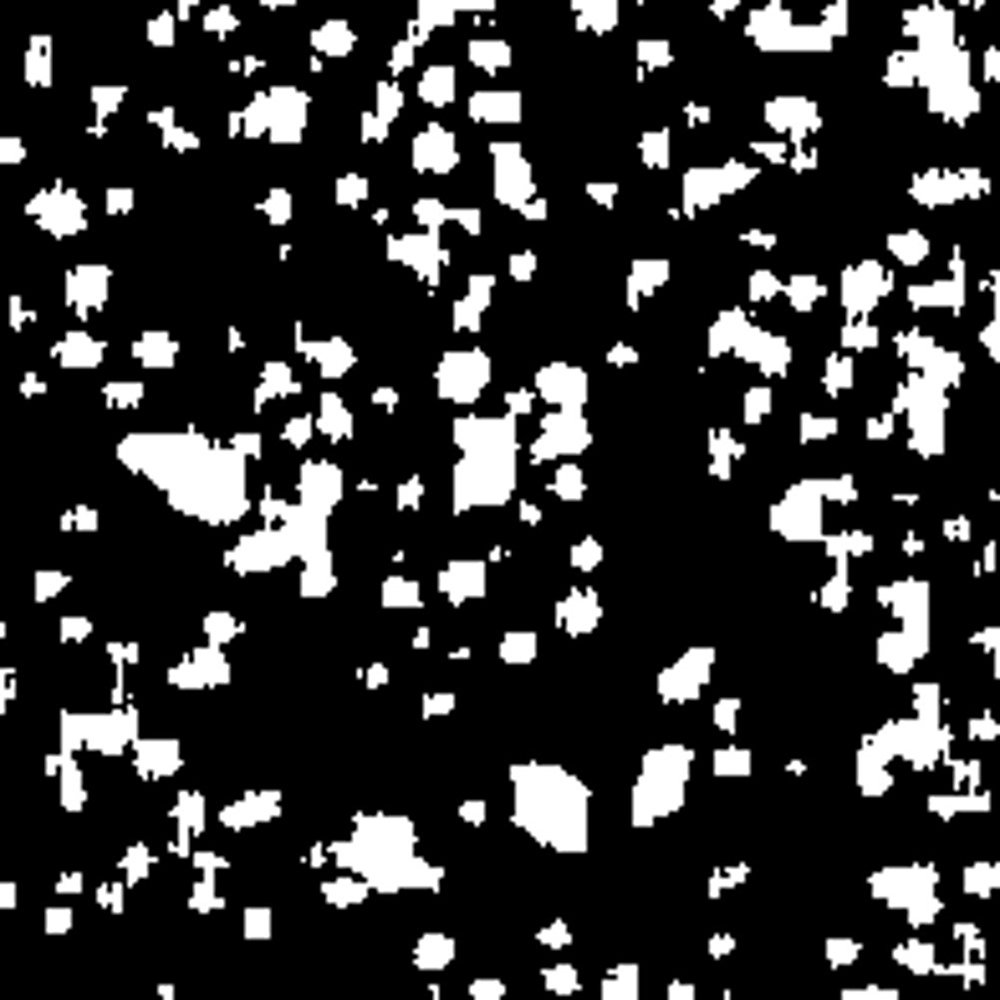

Supplement: Supplementary file 3 — Source Data [file 41467_2023_38178_MOESM3_ESM.zip › Source Data/Fig 3/78.jpg]

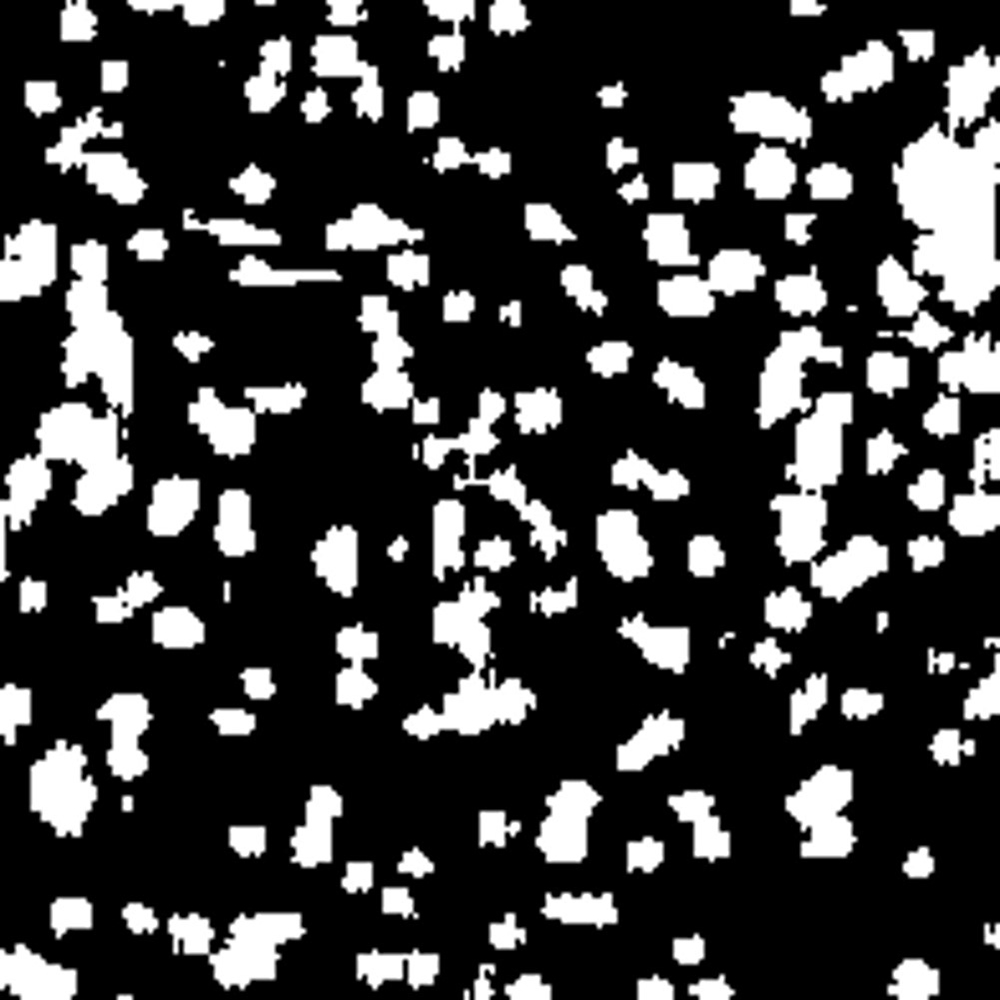

Supplement: Supplementary file 3 — Source Data [file 41467_2023_38178_MOESM3_ESM.zip › Source Data/Fig 3/79.jpg]

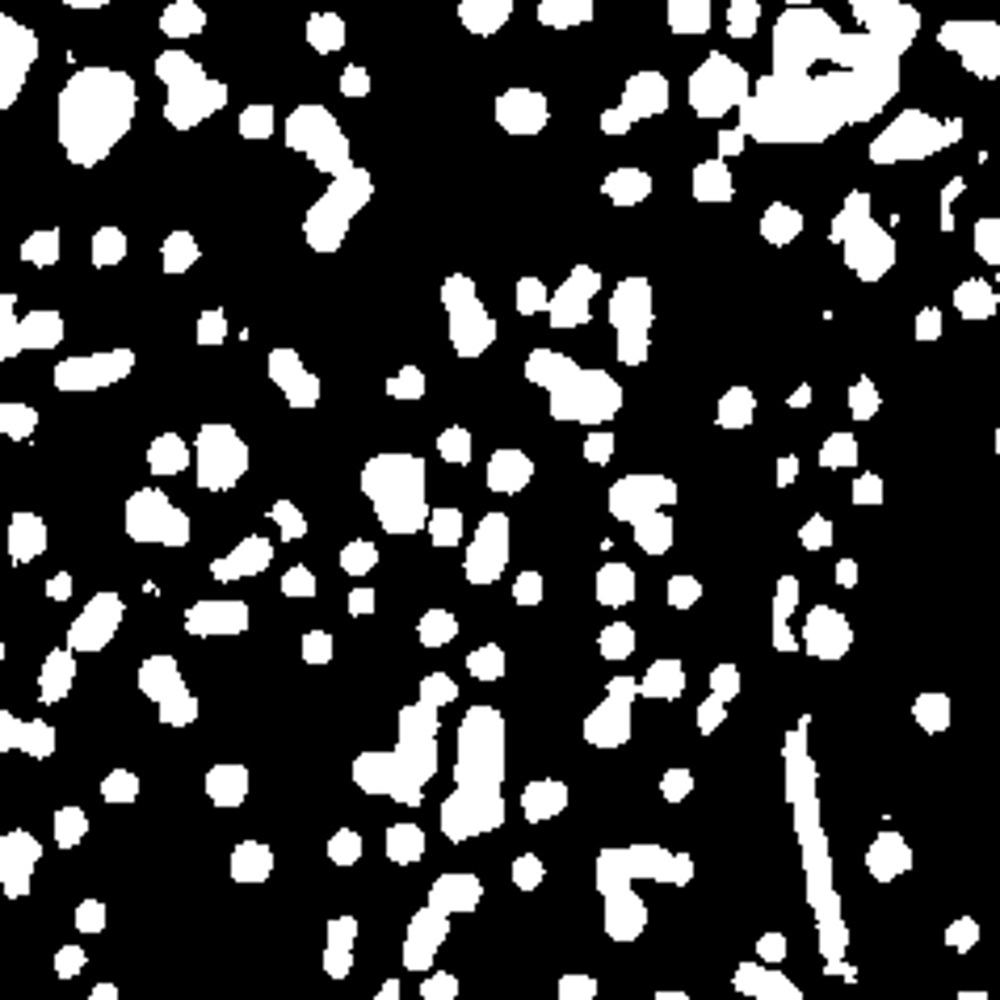

Supplement: Supplementary file 3 — Source Data [file 41467_2023_38178_MOESM3_ESM.zip › Source Data/Fig 3/8.jpg]

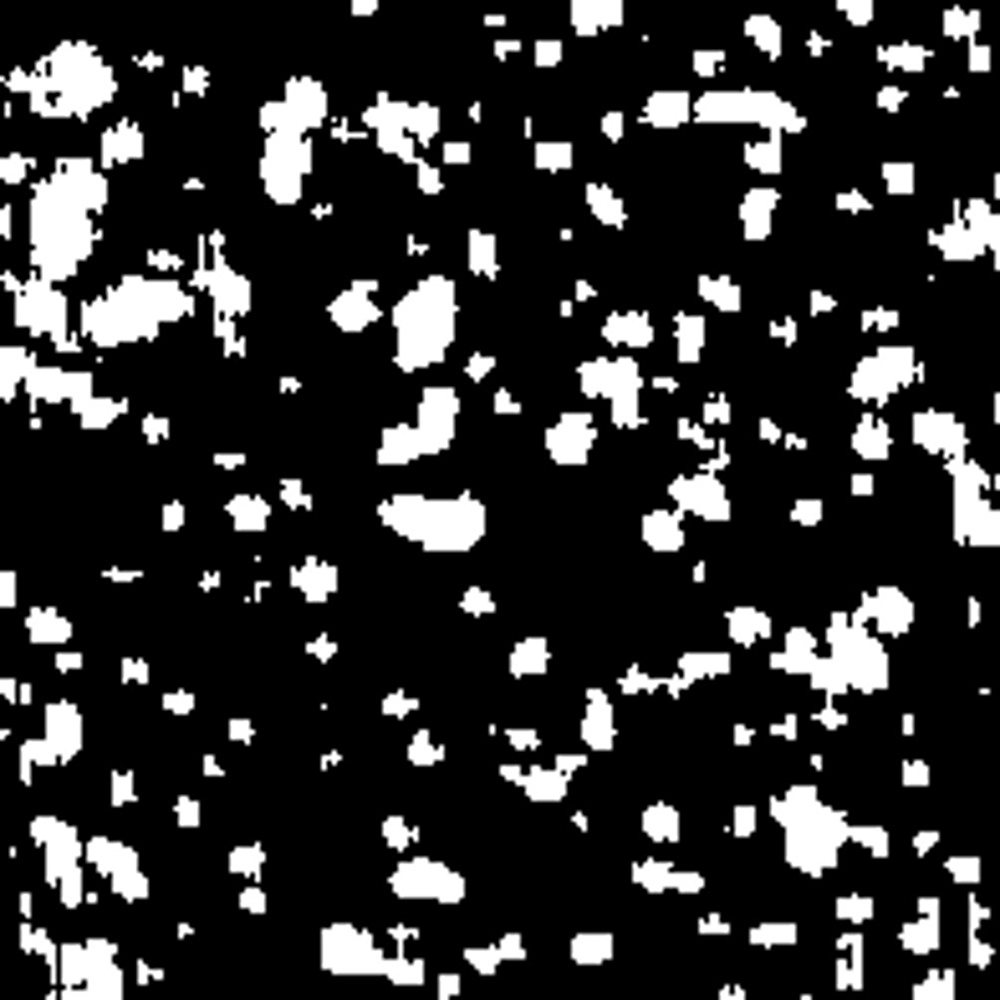

Supplement: Supplementary file 3 — Source Data [file 41467_2023_38178_MOESM3_ESM.zip › Source Data/Fig 3/80.jpg]

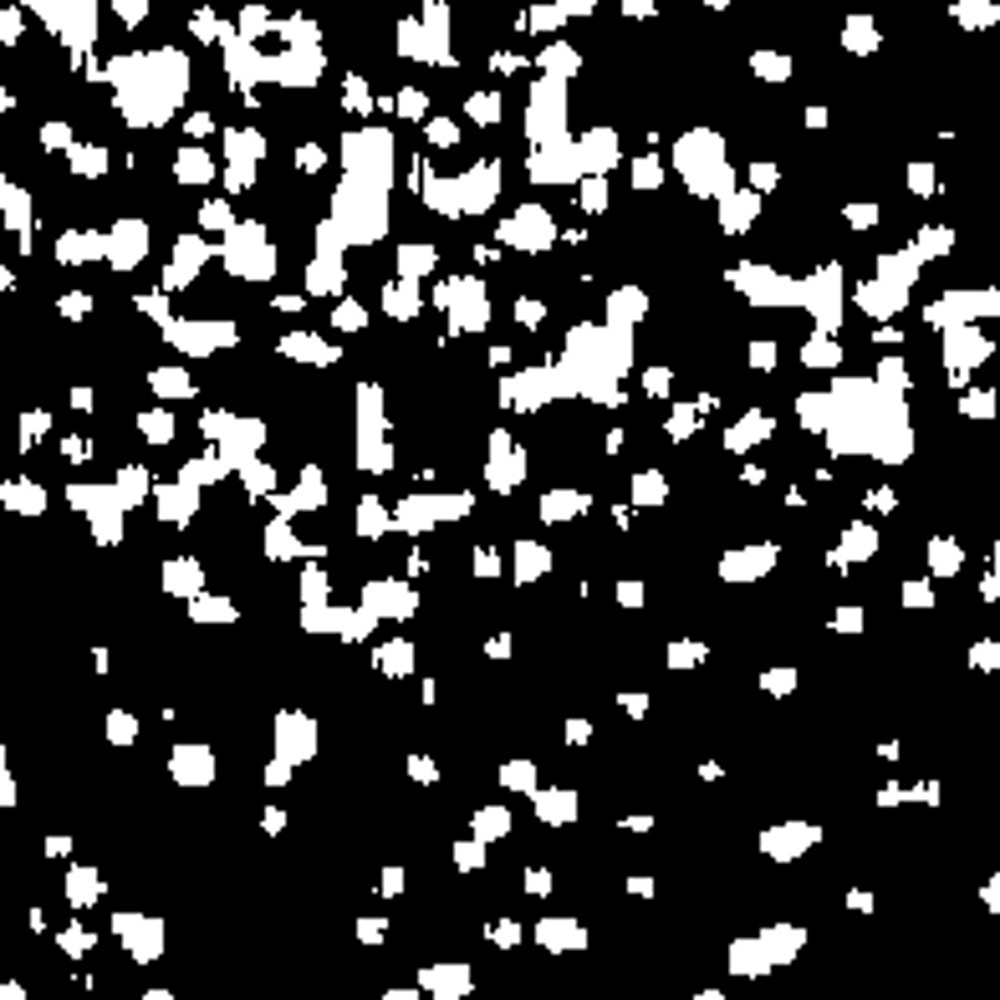

Supplement: Supplementary file 3 — Source Data [file 41467_2023_38178_MOESM3_ESM.zip › Source Data/Fig 3/81.jpg]

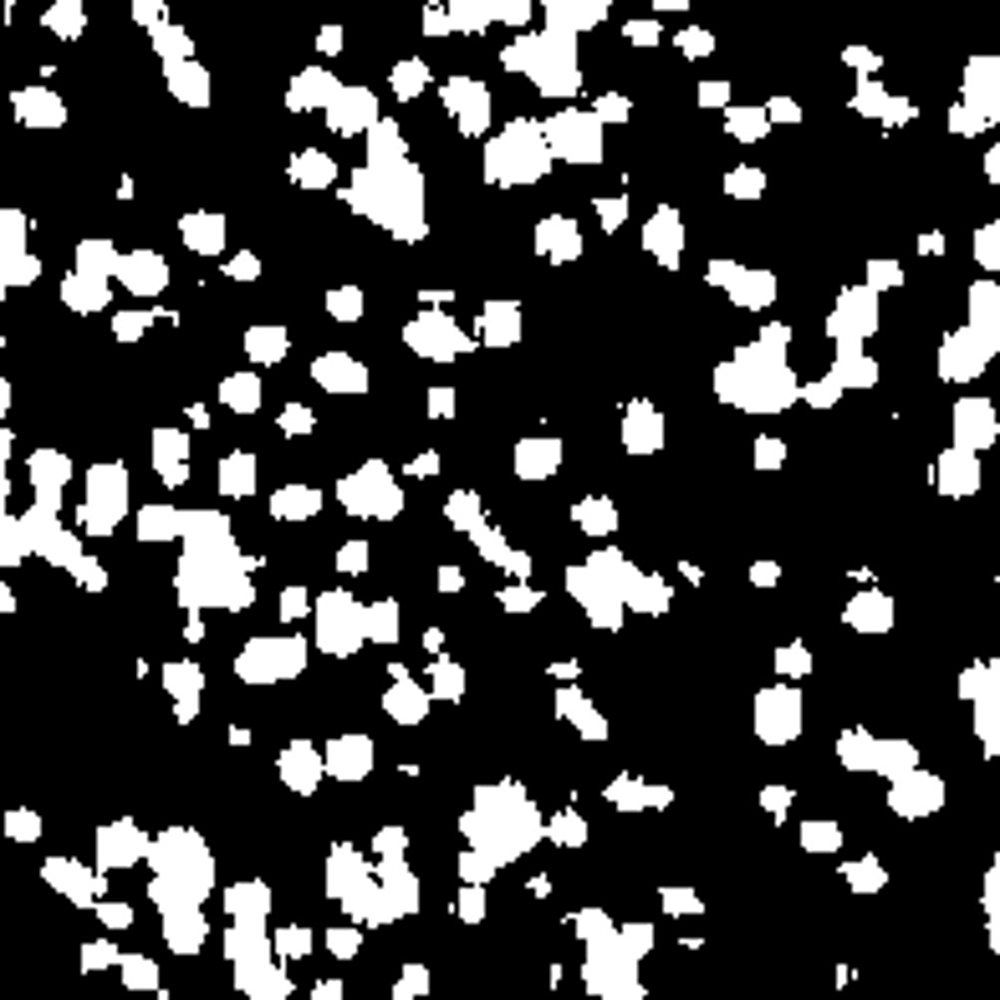

Supplement: Supplementary file 3 — Source Data [file 41467_2023_38178_MOESM3_ESM.zip › Source Data/Fig 3/82.jpg]

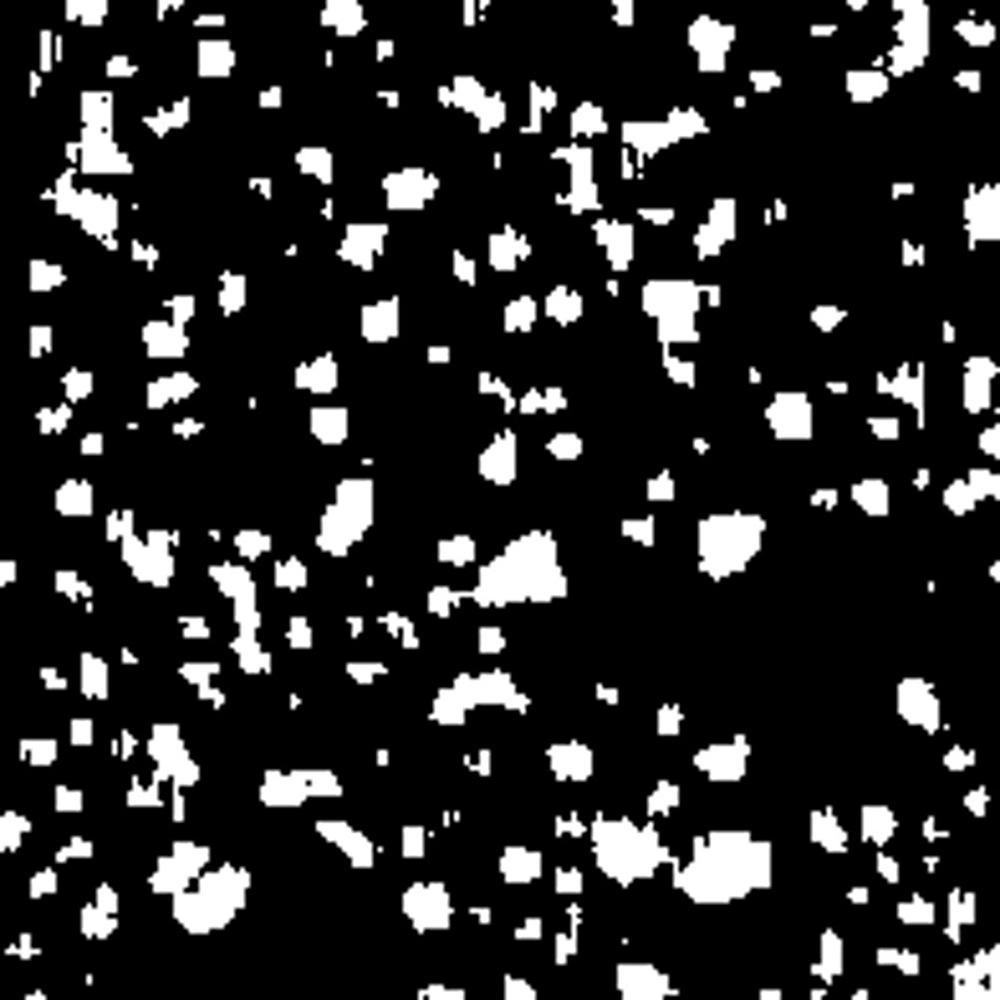

Supplement: Supplementary file 3 — Source Data [file 41467_2023_38178_MOESM3_ESM.zip › Source Data/Fig 3/83.jpg]

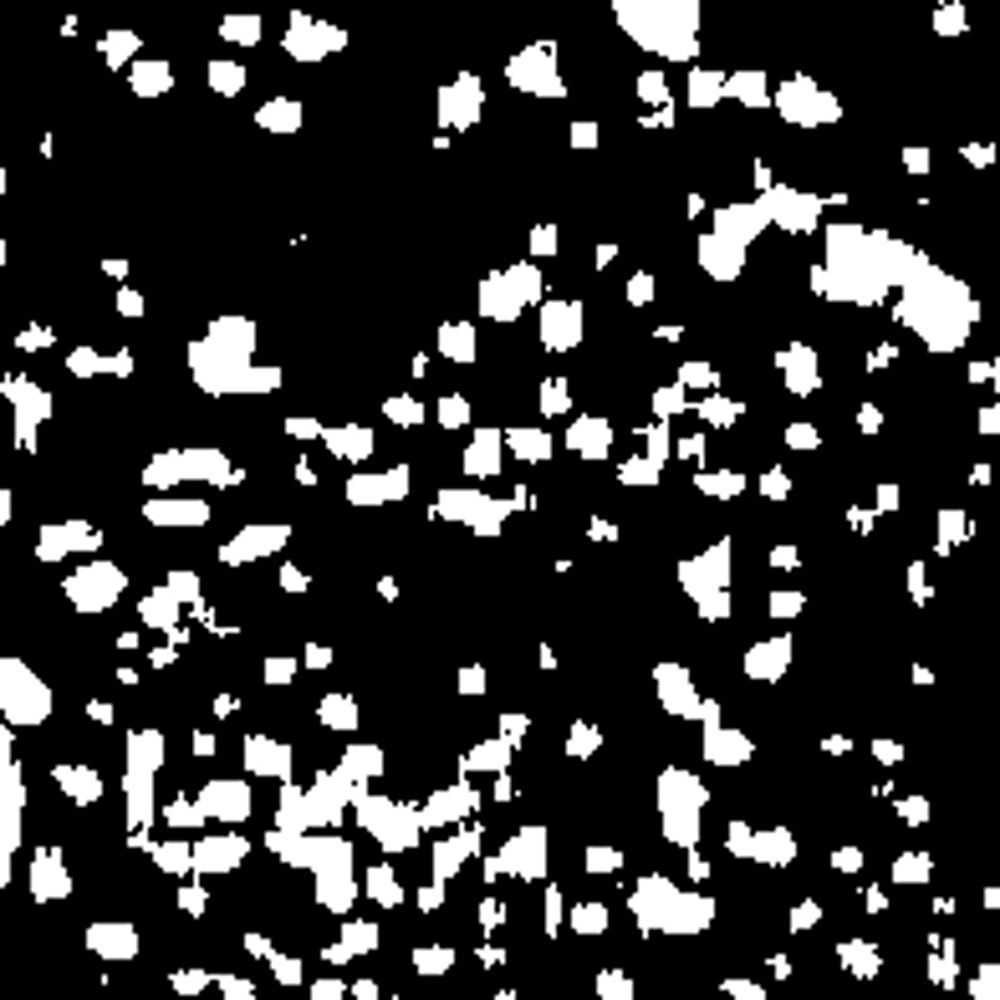

Supplement: Supplementary file 3 — Source Data [file 41467_2023_38178_MOESM3_ESM.zip › Source Data/Fig 3/84.jpg]

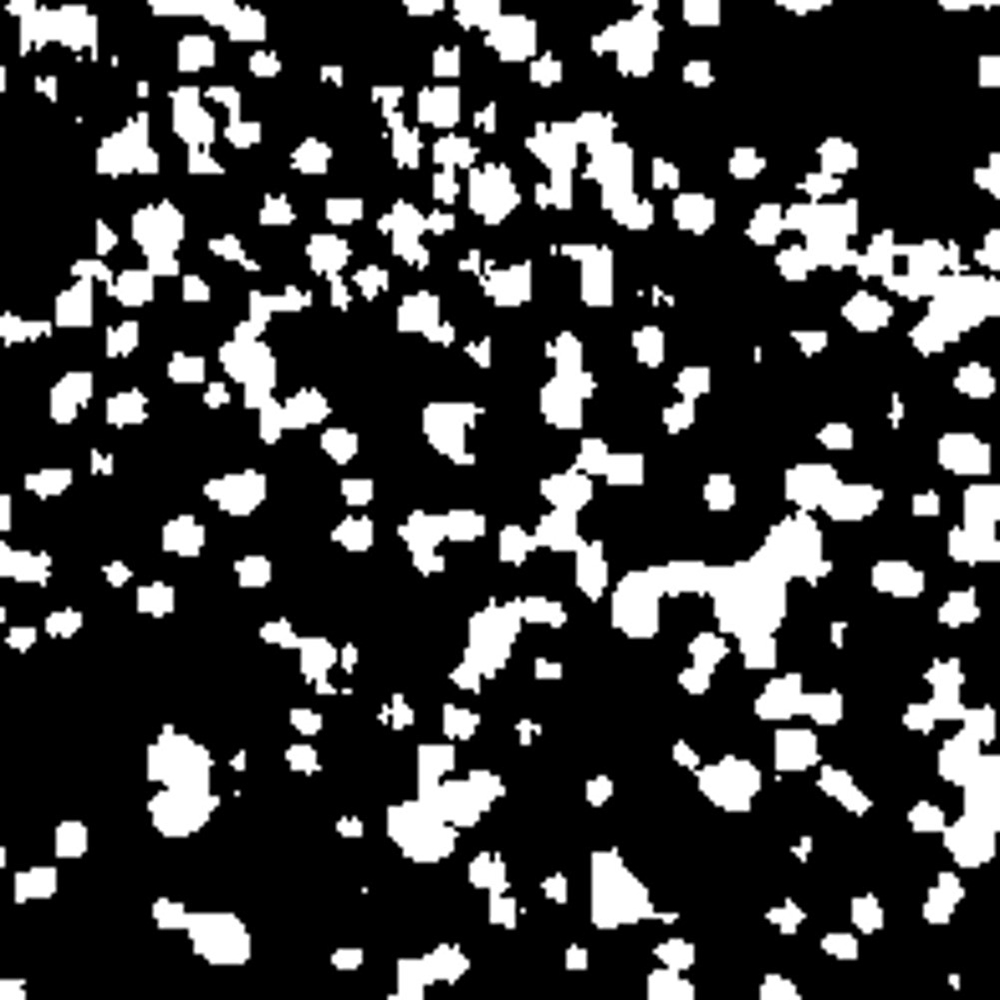

Supplement: Supplementary file 3 — Source Data [file 41467_2023_38178_MOESM3_ESM.zip › Source Data/Fig 3/85.jpg]

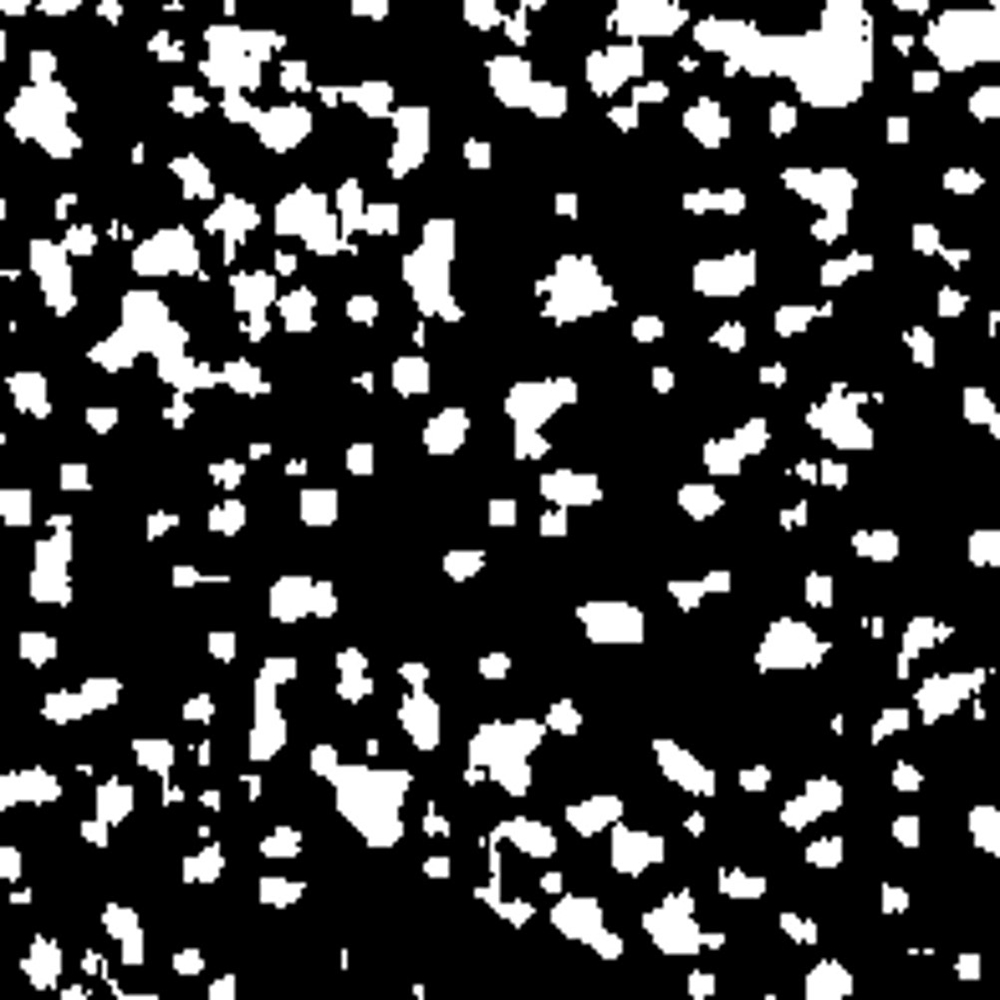

Supplement: Supplementary file 3 — Source Data [file 41467_2023_38178_MOESM3_ESM.zip › Source Data/Fig 3/86.jpg]

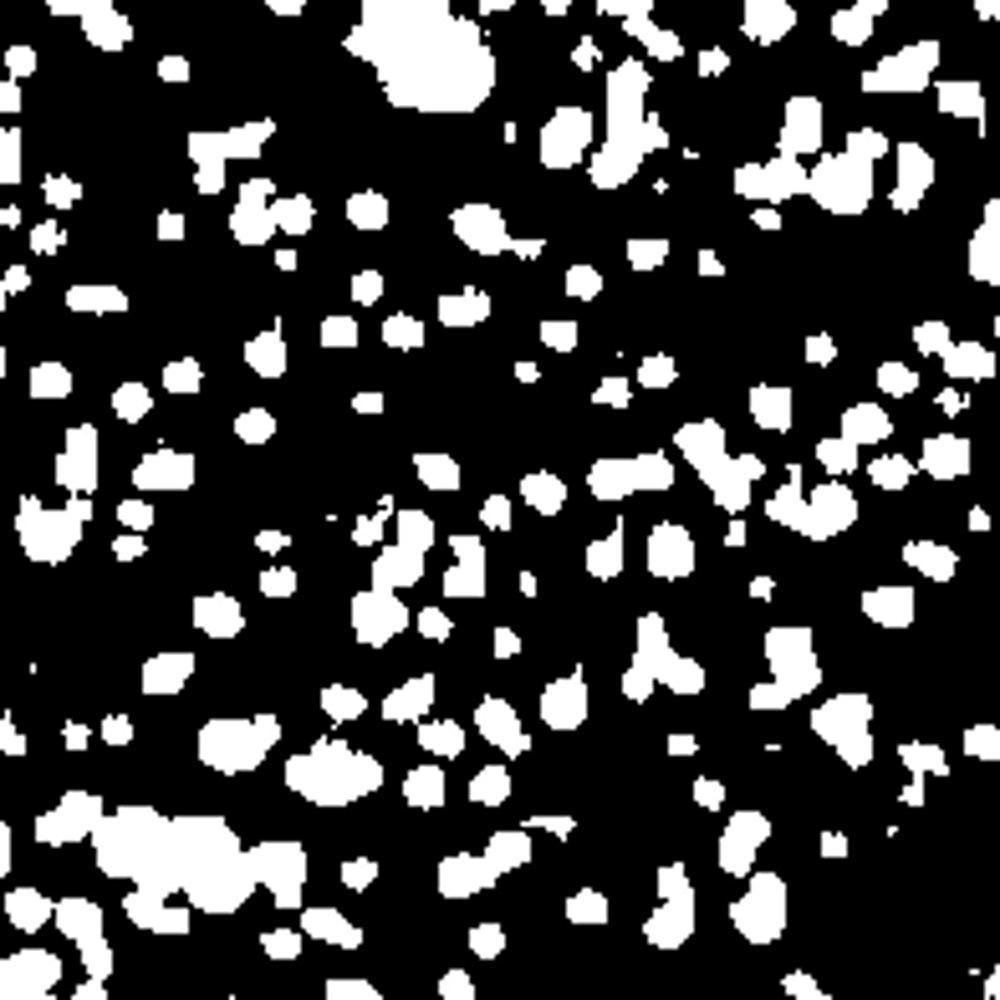

Supplement: Supplementary file 3 — Source Data [file 41467_2023_38178_MOESM3_ESM.zip › Source Data/Fig 3/87.jpg]

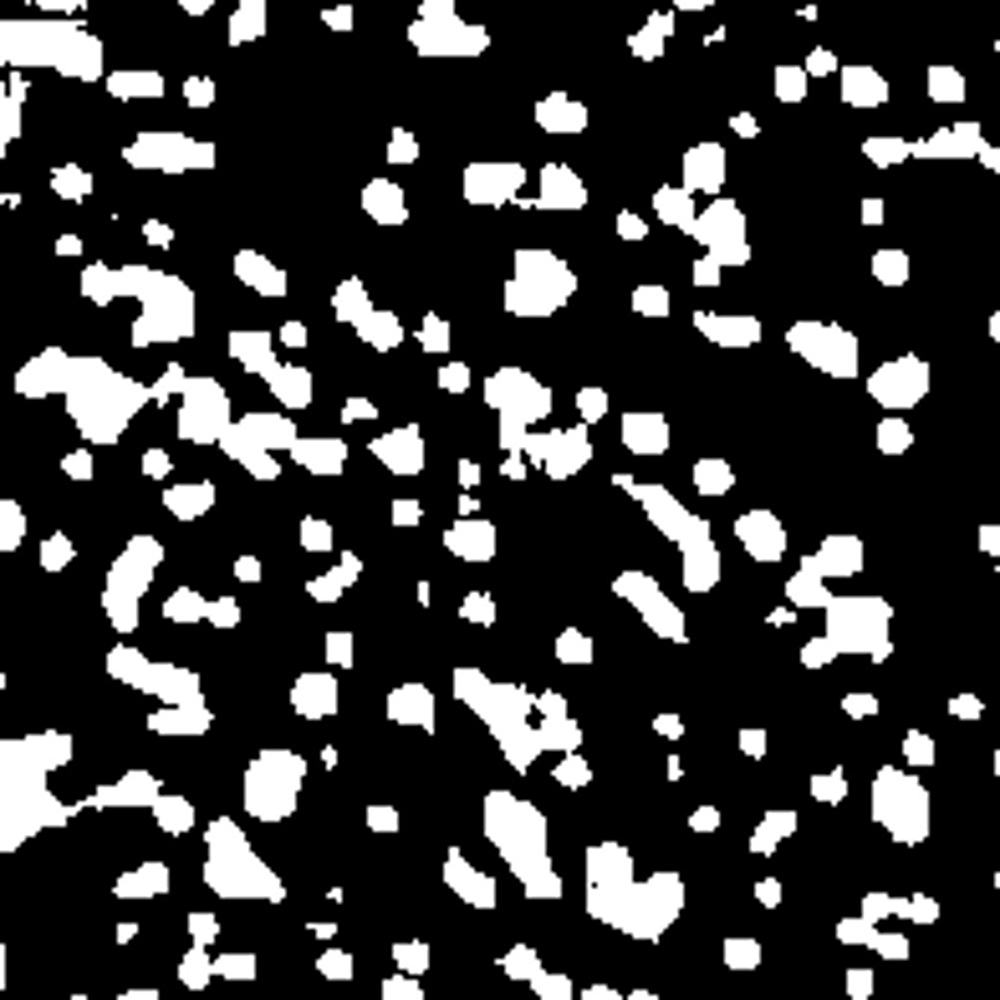

Supplement: Supplementary file 3 — Source Data [file 41467_2023_38178_MOESM3_ESM.zip › Source Data/Fig 3/88.jpg]

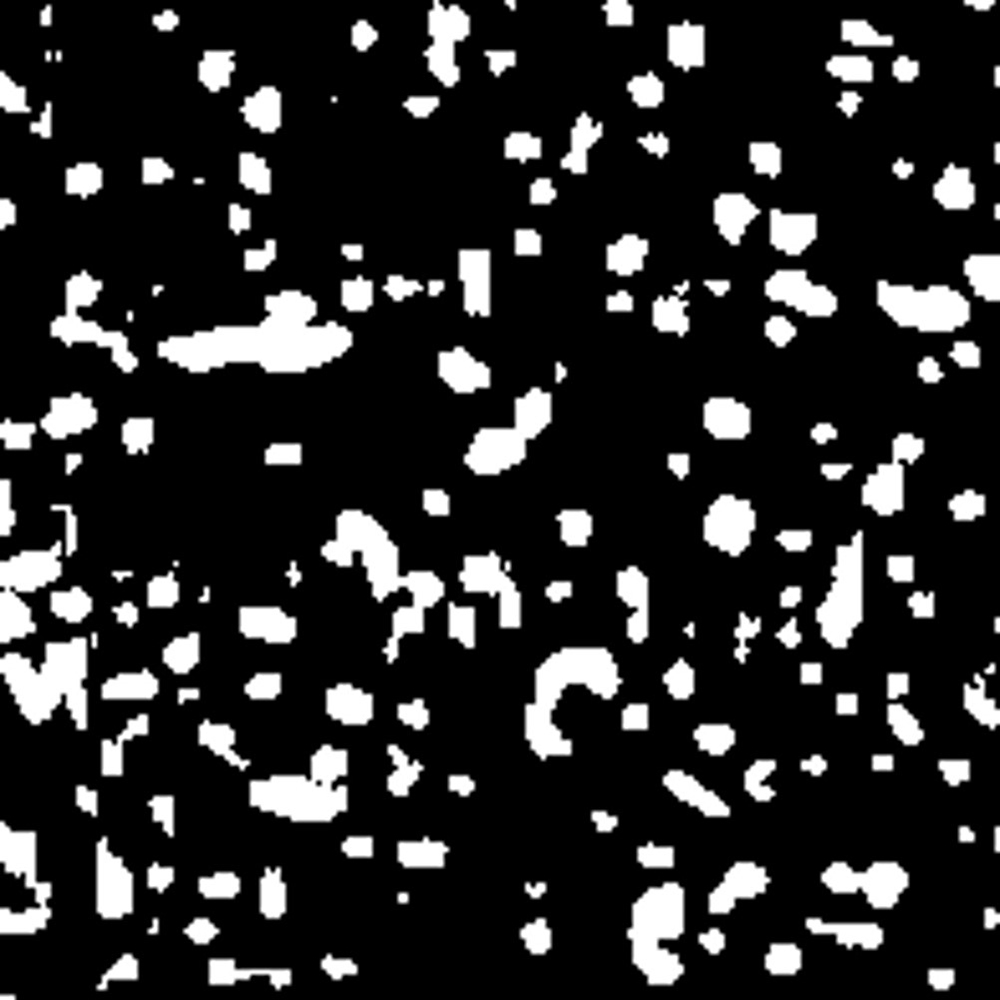

Supplement: Supplementary file 3 — Source Data [file 41467_2023_38178_MOESM3_ESM.zip › Source Data/Fig 3/89.jpg]

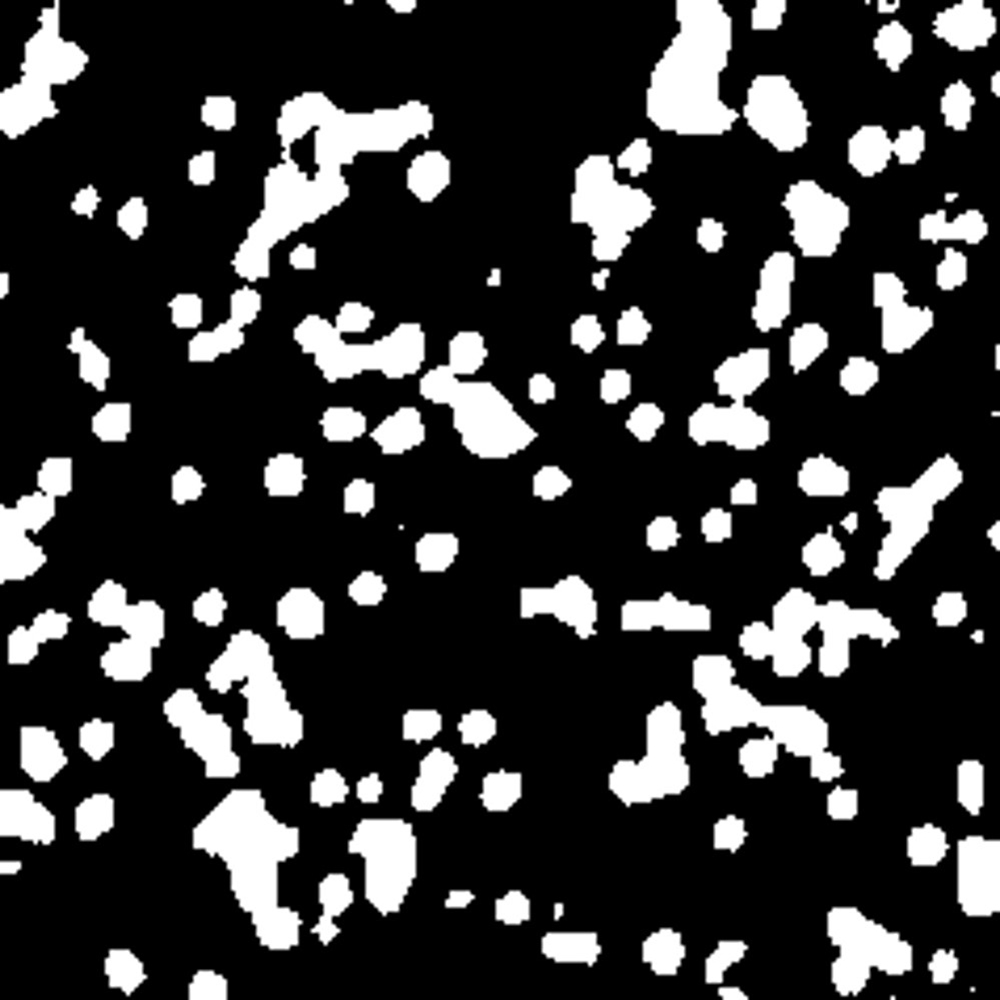

Supplement: Supplementary file 3 — Source Data [file 41467_2023_38178_MOESM3_ESM.zip › Source Data/Fig 3/9.jpg]

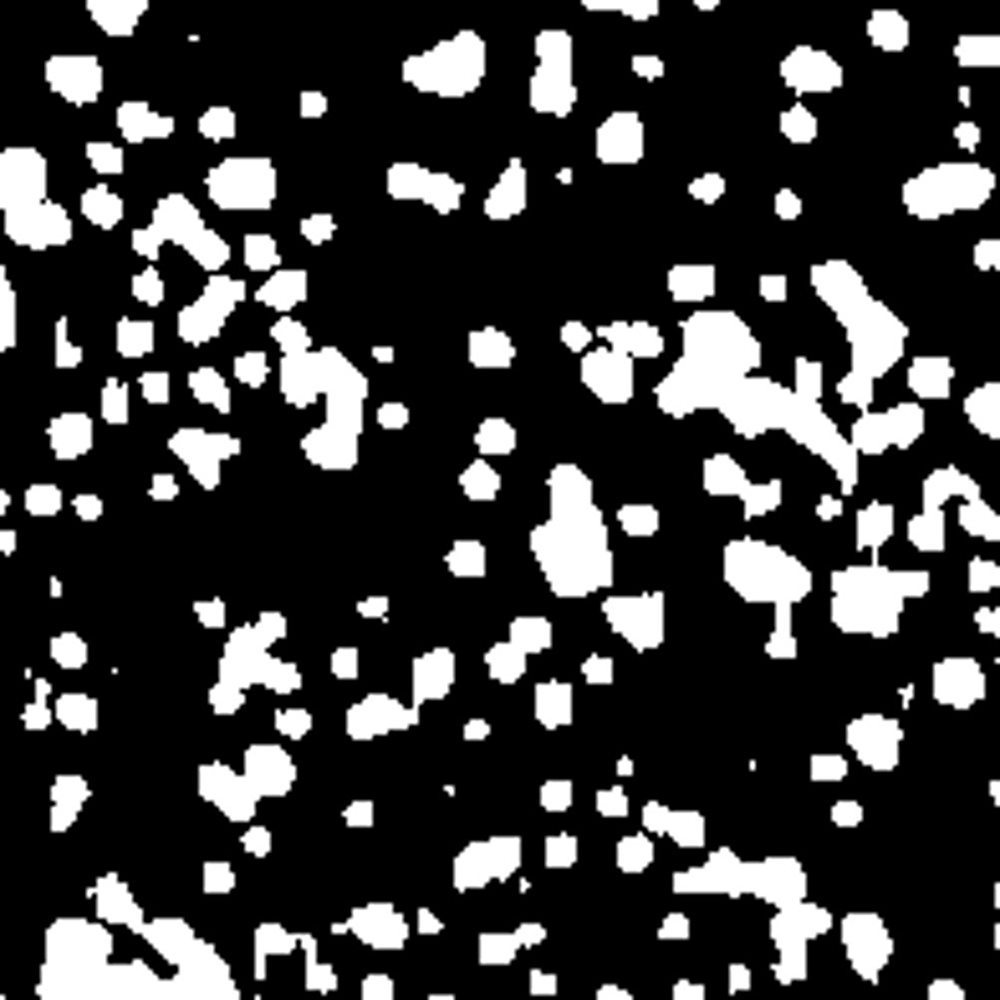

Supplement: Supplementary file 3 — Source Data [file 41467_2023_38178_MOESM3_ESM.zip › Source Data/Fig 3/90.jpg]

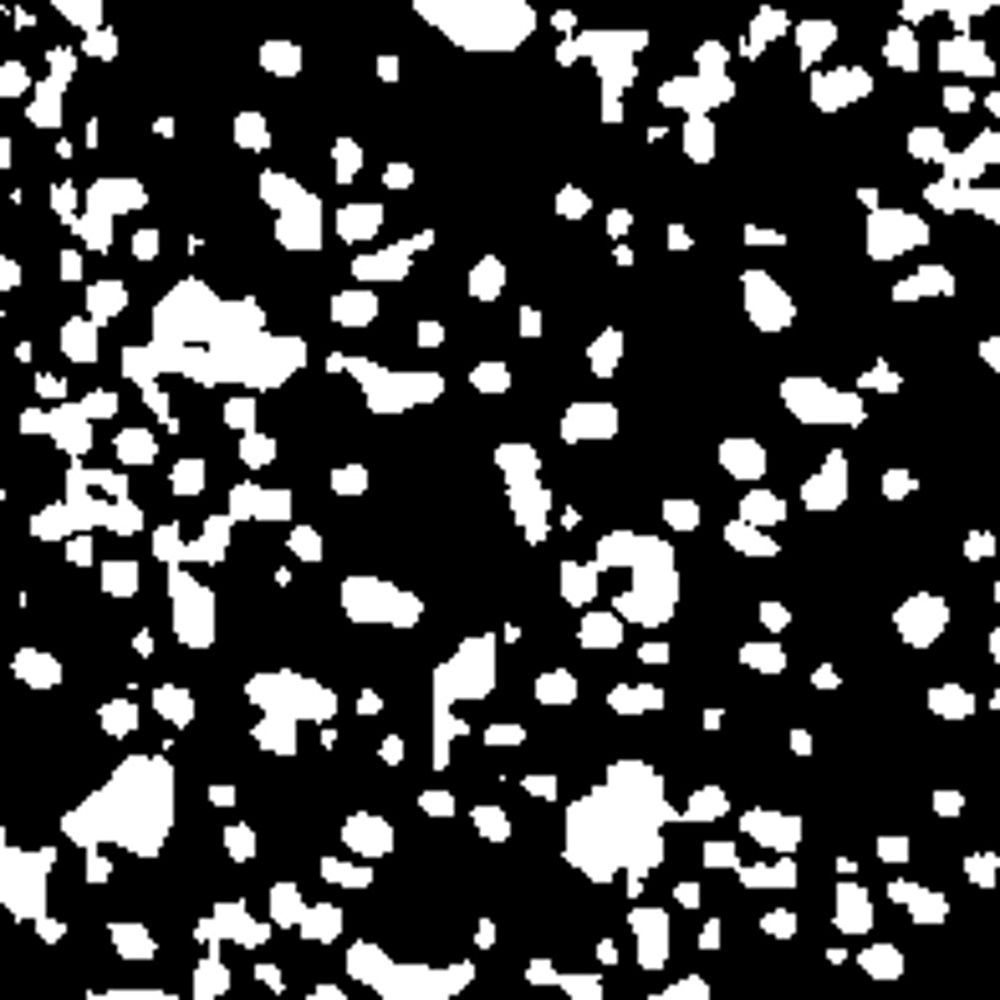

Supplement: Supplementary file 3 — Source Data [file 41467_2023_38178_MOESM3_ESM.zip › Source Data/Fig 3/91.jpg]

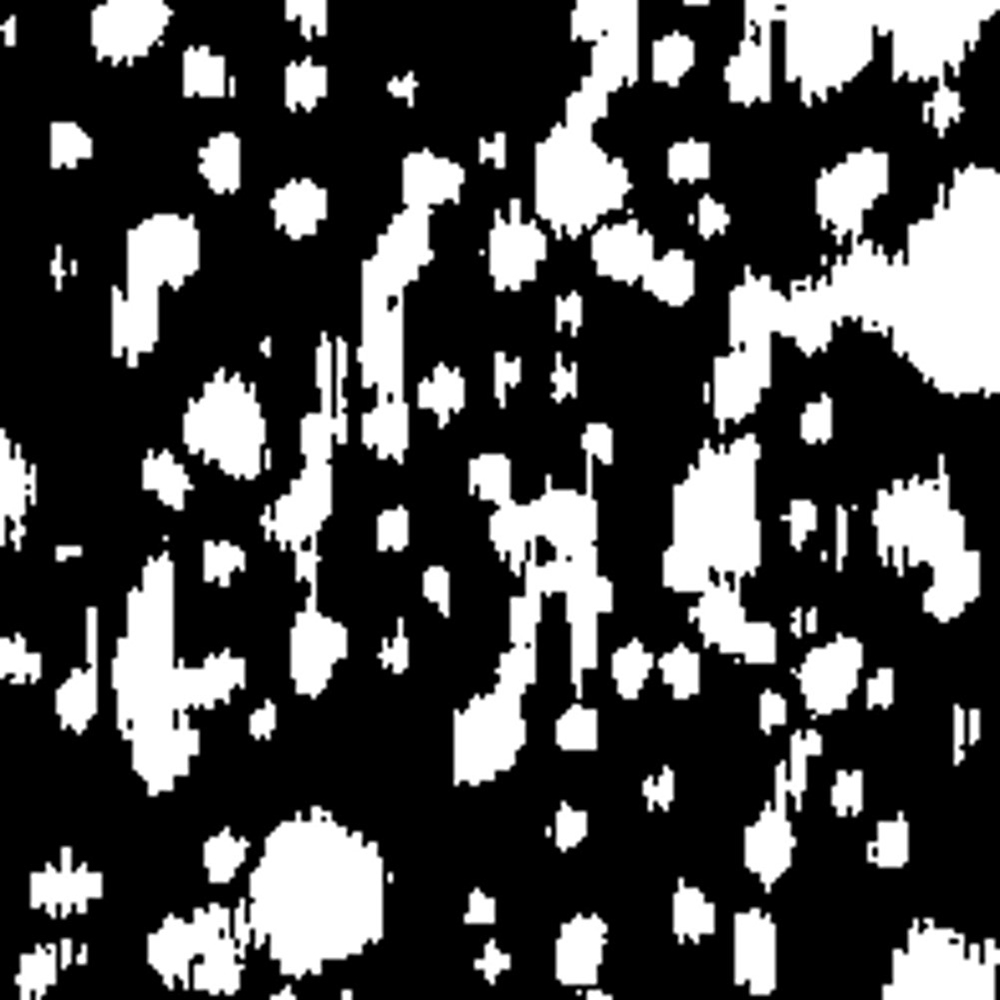

Supplement: Supplementary file 3 — Source Data [file 41467_2023_38178_MOESM3_ESM.zip › Source Data/Fig 3/92.jpg]

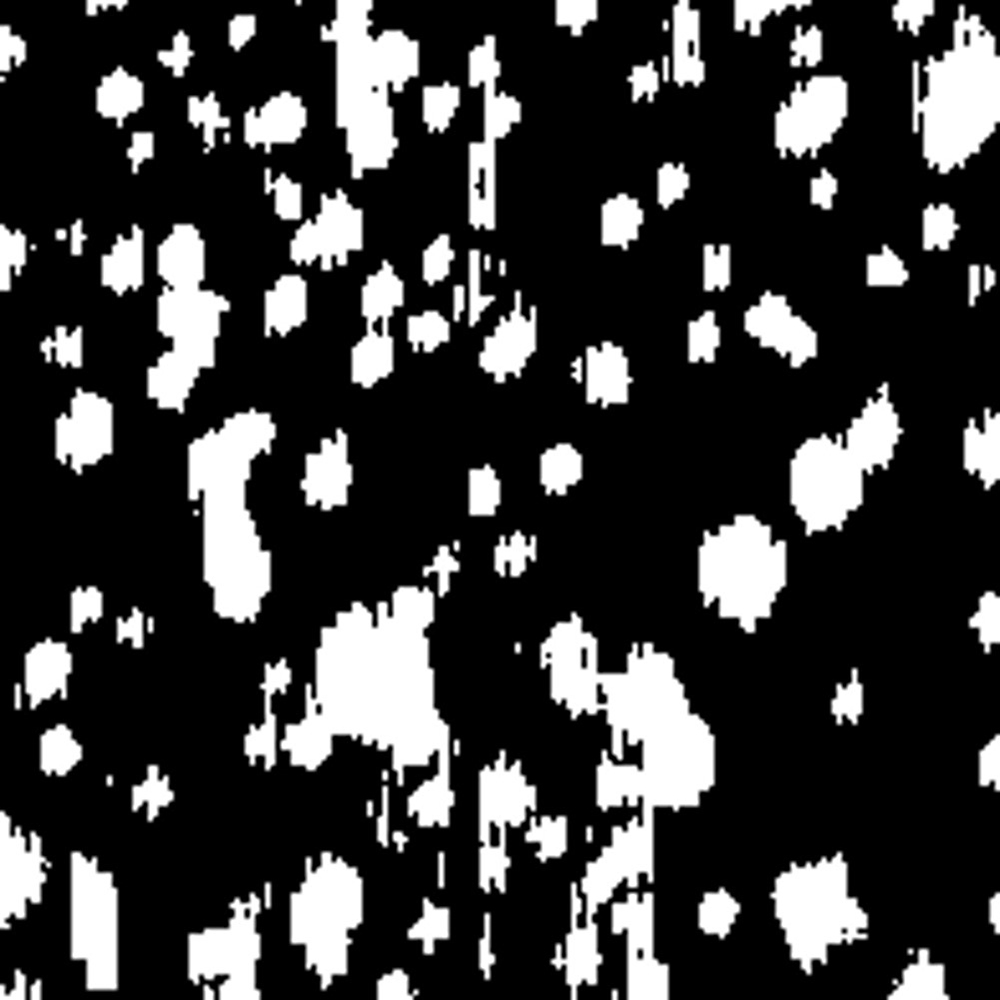

Supplement: Supplementary file 3 — Source Data [file 41467_2023_38178_MOESM3_ESM.zip › Source Data/Fig 3/93.jpg]

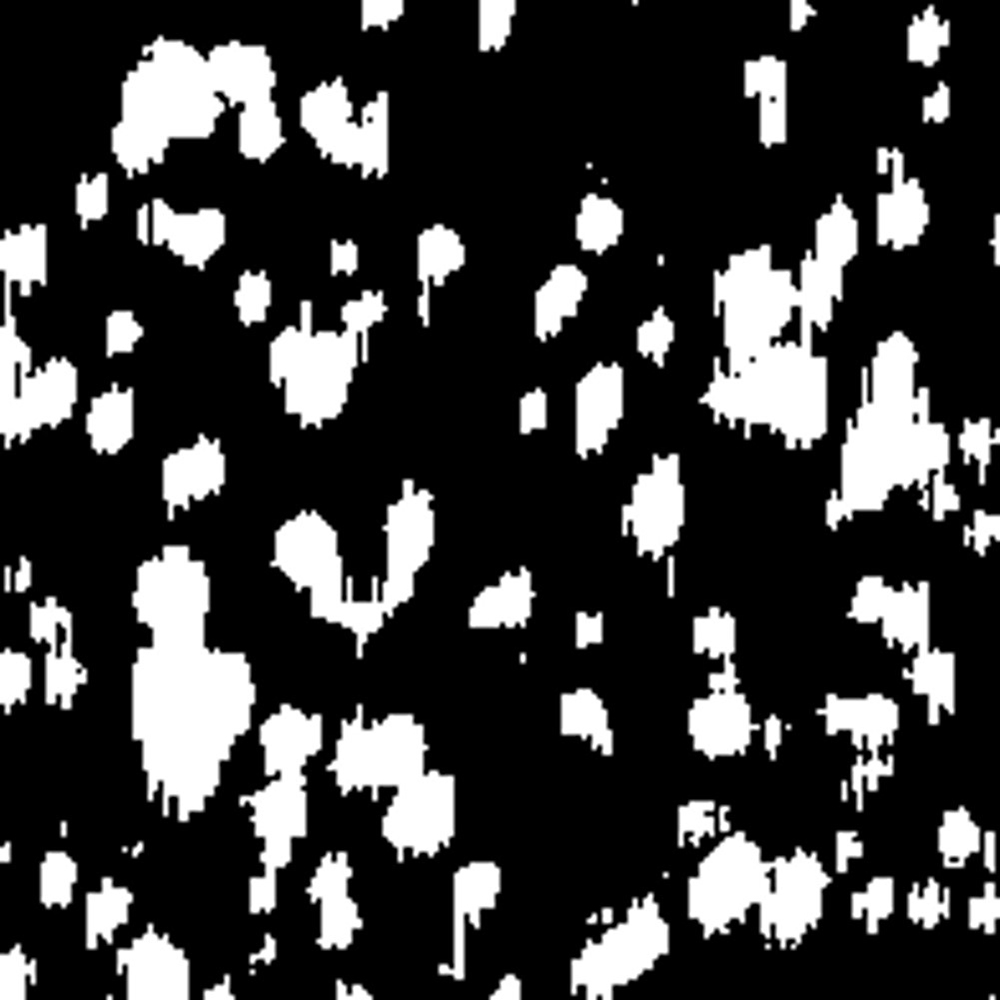

Supplement: Supplementary file 3 — Source Data [file 41467_2023_38178_MOESM3_ESM.zip › Source Data/Fig 3/94.jpg]

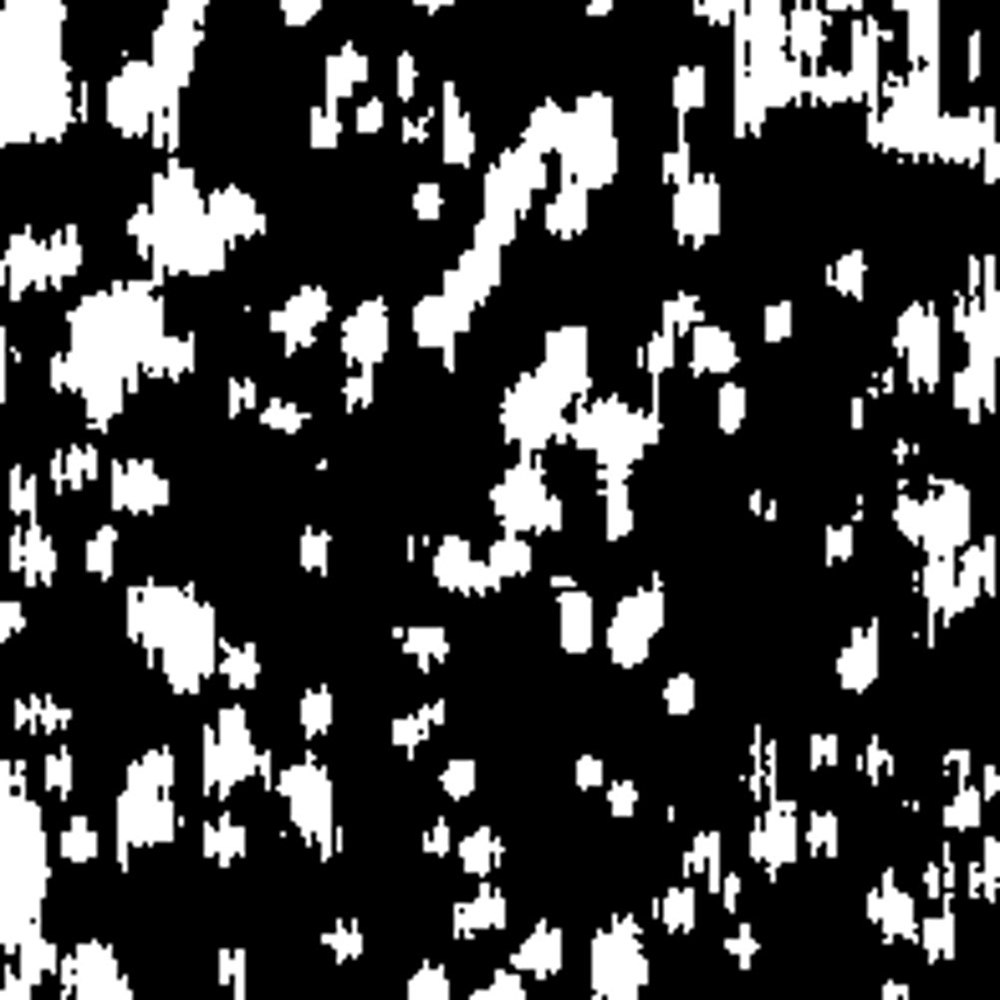

Supplement: Supplementary file 3 — Source Data [file 41467_2023_38178_MOESM3_ESM.zip › Source Data/Fig 3/95.jpg]

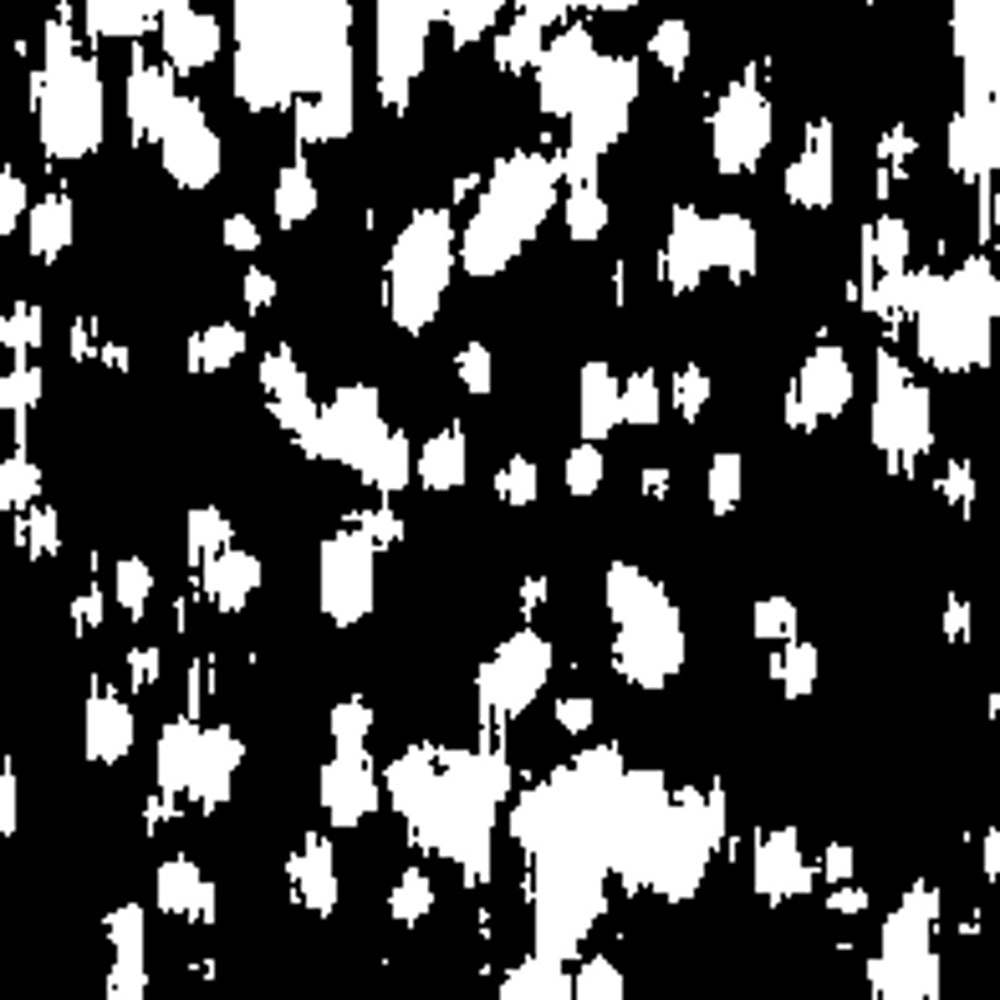

Supplement: Supplementary file 3 — Source Data [file 41467_2023_38178_MOESM3_ESM.zip › Source Data/Fig 3/96.jpg]

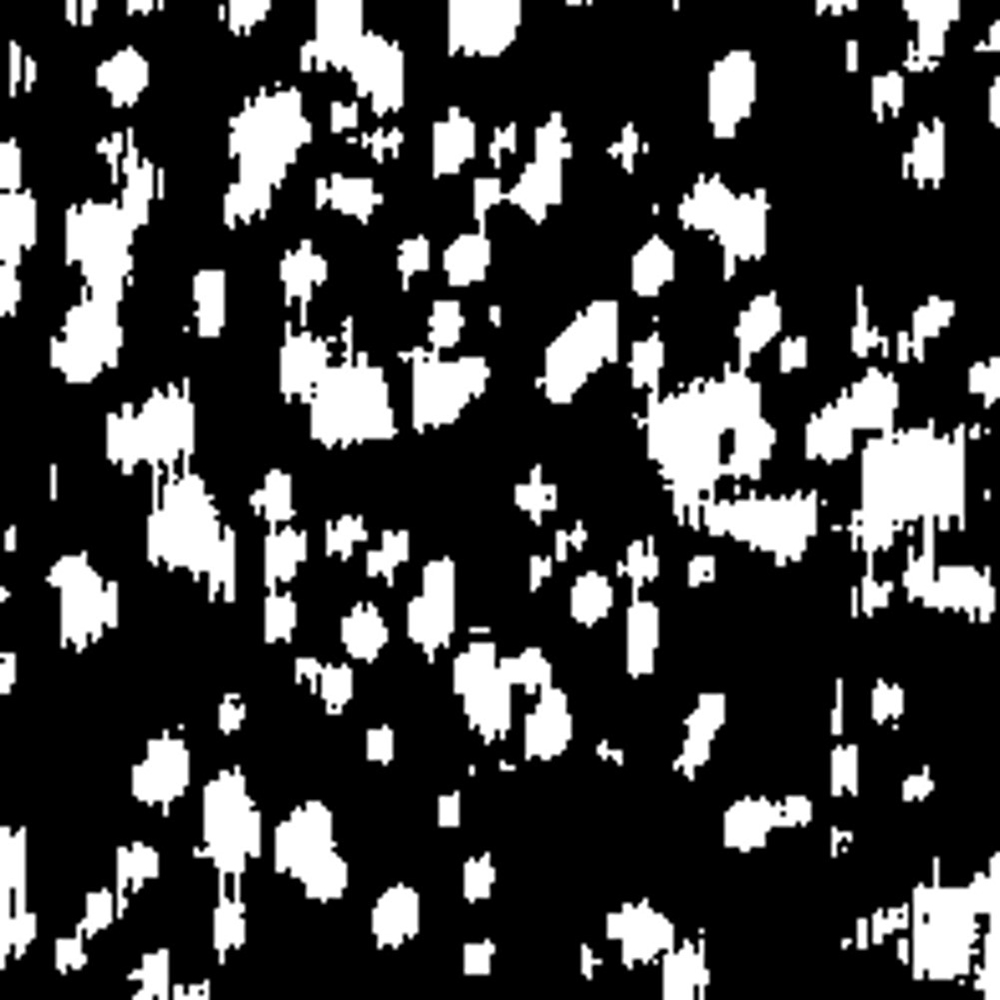

Supplement: Supplementary file 3 — Source Data [file 41467_2023_38178_MOESM3_ESM.zip › Source Data/Fig 3/97.jpg]

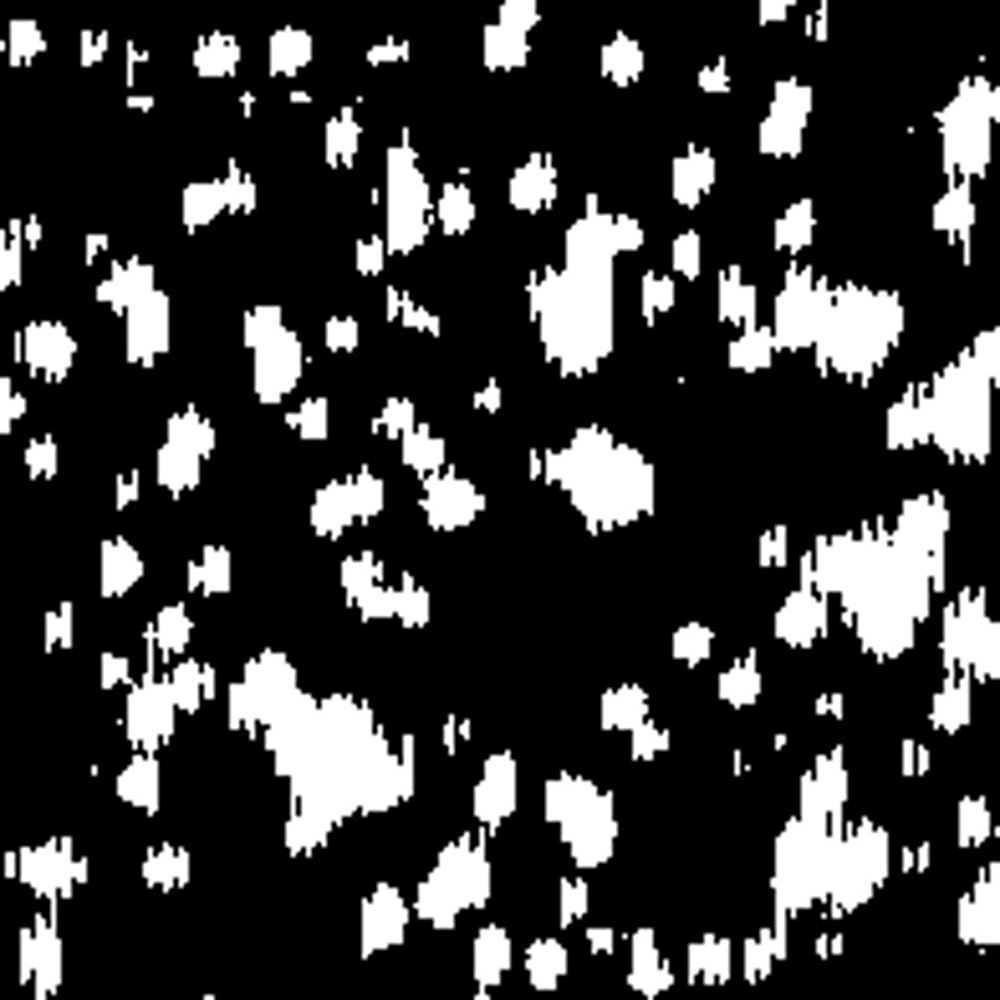

Supplement: Supplementary file 3 — Source Data [file 41467_2023_38178_MOESM3_ESM.zip › Source Data/Fig 3/98.jpg]

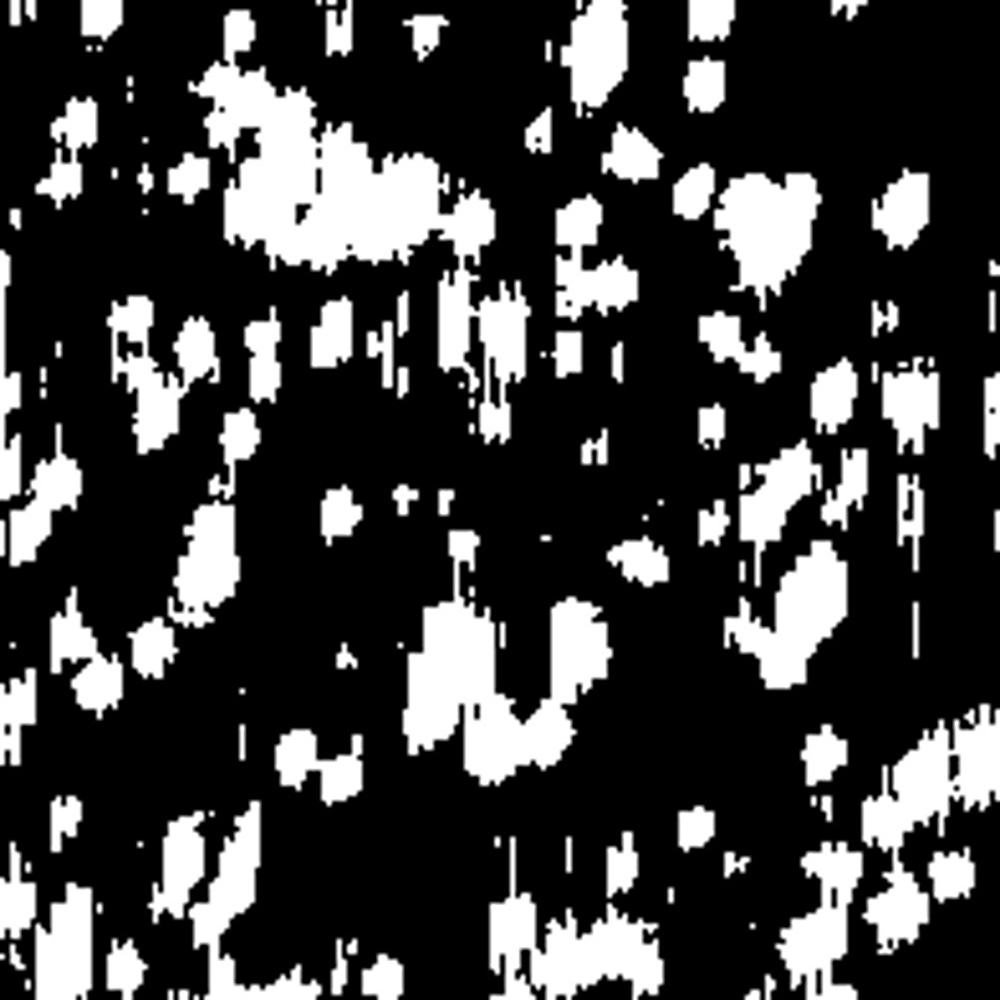

Supplement: Supplementary file 3 — Source Data [file 41467_2023_38178_MOESM3_ESM.zip › Source Data/Fig 3/99.jpg]
